# Fast‐Charging Phosphorus Anodes Enabled by Fluorinated Weakly Solvated Electrolytes for Stable and High‐Rate Lithium Storage

**DOI:** 10.1002/adma.202504248

**Published:** 2025-05-07

**Authors:** Huixian Xie, Lingwen Liu, Hongyi Chen, Kwan San Hui, Zhuoheng Kuang, Guangmin Zhou, Yuanmiao Sun, Hui‐Ming Cheng, Kwun Nam Hui

**Affiliations:** ^1^ Joint Key Laboratory of the Ministry of Education Institute of Applied Physics and Materials Engineering University of Macau Avenida da Universidade Taipa Macau SAR 999078 P. R. China; ^2^ Department of Mechanical Engineering College of Engineering Prince Mohammad Bin Fahd University P.O. Box 1664 Al Khobar 31952 Kingdom of Saudi Arabia; ^3^ Tsinghua Shenzhen International Graduate School Tsinghua University Shenzhen 518055 P. R. China; ^4^ Shenzhen Key Laboratory of Energy Materials for Carbon Neutrality Institute of Technology for Carbon Neutrality Shenzhen Institutes of Advanced Technology Chinese Academy of Sciences Shenzhen 518055 P. R. China; ^5^ Faculty of Materials Science and Energy Engineering Shenzhen University of Advanced Technology Shenzhen 518055 P. R. China; ^6^ Shenyang National Laboratory for Materials Science Institute of Metal Research Chinese Academy of Sciences Shenyang 110016 P. R. China

**Keywords:** fast charging, interfacial stability, phosphorus‐based anode, weakly solvated electrolytes

## Abstract

Phosphorus‐based anodes hold promise for energy storage due to their high theoretical capacity and favorable lithiation potential. However, their practical application is hindered by sluggish reaction kinetics and irreversible capacity loss, primarily attributed to multiphase lithiation/delithiation reactions and the dissolution of lithium polyphosphide intermediates. Herein, a universal design principle of weakly solvated electrolytes (WSEs) tailored for phosphorus‐based anodes is proposed. Combined with a high dielectric constant, and significant dipole moment, a fluorinated cosolvent is incorporated into a WSE to effectively suppress the dissolutions of lithium polyphosphides, enhance interfacial stability, and accelerate reaction kinetics. With this electrolyte, a phosphorus‐based anode achieves a remarkable capacity of 2615.2 mAh g⁻¹ at 1C, maintaining 91.7% capacity retention over 1000 cycles. Even at a high rate of 4 C, it delivers 2210.7 mAh g⁻¹ with an exceptional retention of 96.7% after 1500 cycles. Furthermore, at 0 °C, the anode sustains a capacity of 2016.7 mAh g⁻¹, with 97% retention after 300 cycles at 1C. This study provides a novel electrolyte design strategy to regulate the solvation sheath, paving the way for high‐rate, long‐cycle phosphorus‐based anodes suitable for fast‐charging applications.

## Graphical abstract

A universal weakly‐solvated electrolyte design principle is established for phosphorus‐based anodes through systematic evaluation. Based on this criterion, the fluorinated solvent of FEC emerges as the optimal co‐solvent, effectively suppressing the dissolution of lithium polyphosphides while enhancing desolvation/charge‐transfer kinetics and simultaneously fostering a stable inorganic‐rich SEI layer. This rational strategy addresses critical interfacial challenges in high‐performance phosphorus‐based battery systems.

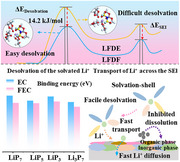

## 1 Introduction

Increasing the power density of lithium‐ion batteries (LIBs) is crucial for advancing the performance of portable electronics and electric vehicles. While commercial graphite remains the dominant anode material, its low capacity, poor rate capability, and susceptibility to lithium plating present significant limitations. Black phosphorus (BP) has emerged as a promising alternative due to its high electrical conductivity of 300 S m⁻¹,^[^ ^1^ ^]^ ultra‐low Li^+^ diffusion barrier (0.08 eV), and exceptional gravimetric capacity of 2596 mAh g⁻¹.^[^ ^2^ ^]^ These attributes help mitigate BP's relatively high voltage loss (versus Li/Li⁺), enabling a high gravimetric energy density, particularly for fast‐charging applications. However, the Li alloying–dealloying process in BP induces severe volume expansion, leading to continuous rupture and reformation of the solid electrolyte interphase (SEI).^[^ ^3^ ^]^ Furthermore, its reaction pathway involves complex phase transitions, progressing from LiP_7_ to LiP_5_, Li_3_P_7_, LiP, and finally Li_3_P,^[^ ^4^ ^]^ generating soluble intermediates such as LiP_7_, P_5_⁻, LiP_6_⁻, LiP_3_, P_3_⁻, and P⁻, along with solvated species like [P_7_·2EC]⁻, [P_7_·EC]⁻, and [2LiP_3_·EC]⁻.^[^ ^5^ ^]^ The dissolution of these intermediates contributes to large polarization, sluggish reaction kinetics, and active material loss, severely impacting electrochemical stability.

To address these challenges, strategies involving physical confinement using carbon nanotubes (CNTs),^[^ ^6^ ^]^ and chemical adsorption via lithium fluoride (LiF)^[^ ^5^ ^]^ or ionic covalent organic frameworks have been explored.^[^ ^7^ ^]^ Beyond structural modifications, electrolyte engineering offers an effective route to stabilizing the electrode–electrolyte interface and enhancing reaction kinetics. For instance, fluoroethylene carbonate (FEC) is widely used as an electrolyte additive to promote SEI stability.^[^ ^8^ ^]^ Additionally, the incorporation of triethyl phosphate (TEP), FEC, and lithium bis(fluorosulfonyl)imide (LiFSI) into the solvation sheath further improves interfacial stability.^[^ ^9^ ^]^ Moreover, the use of 1,1,2,2‐tetrafluoroethyl‐2,2,3,3‐tetrafluoropropylether (TTE) in a LiFSI/TEP‐based electrolyte enables the formation of an anion‐rich solvation sheath, facilitating the development of a fluorine‐rich inorganic SEI.^[^ ^10^ ^]^ Despite these advancements, existing electrolytes have yet to fully unlock the fast‐charging potential of phosphorus‐based anodes, and a universal design strategy for achieving this remains undefined. Therefore, the development of a comprehensive electrolyte design principle tailored for phosphorus‐based anodes is urgently needed to enable their practical implementation in high‐power LIBs. Herein, we report an inclusive design principle of weakly solvated electrolytes (WSEs) based on various cosolvents with distinct dielectric properties—including 1,3‐dioxolane (DOL), TEP, FEC, and ethylene carbonate (EC). Three synergistic factors of high dielectric constant, strong dipole moment, and strategic fluorination were identified as critical for electrolyte optimization. A high dielectric constant is required to enhance the overall solvation ability, a significant dipole moment is necessary to further facilitate the coordination dynamics and the presence of fluorination is beneficial for the introduction of FSI^−^ anions.

Among the cosolvents examined, FEC was identified as the most effective, significantly mitigating the dissolution of lithium polyphosphides (LiPPs), enhancing reaction kinetics, and stabilizing phosphorus‐based anodes. The optimized WSE formulation enables exceptional electrochemical performance, with the anode delivering a high capacity of 2615.2 mAh g⁻¹ at 1C, exhibiting minimal capacity degradation. Even under high‐rate conditions (4C), the anode retains a substantial capacity of 2210.7 mAh g⁻¹ and demonstrates remarkable cycling stability, with 96.7% capacity retention over 1500 cycles. Notably, at 0 °C, the anode maintains a capacity of 2016.7 mAh g⁻¹ with 97% retention after 300 cycles at 1C, underscoring the electrolyte's robust performance across a wide temperature range. This designed WSE simultaneously enhances the rate capability, cycle stability, and low‐temperature performance of phosphorus‐based anodes, representing a significant advancement in electrolyte engineering for next‐generation high‐energy‐density batteries.

## 2 Results and Discussion

### 2.1 Design Principles for Optimizing WSEs

**Figure 1:**
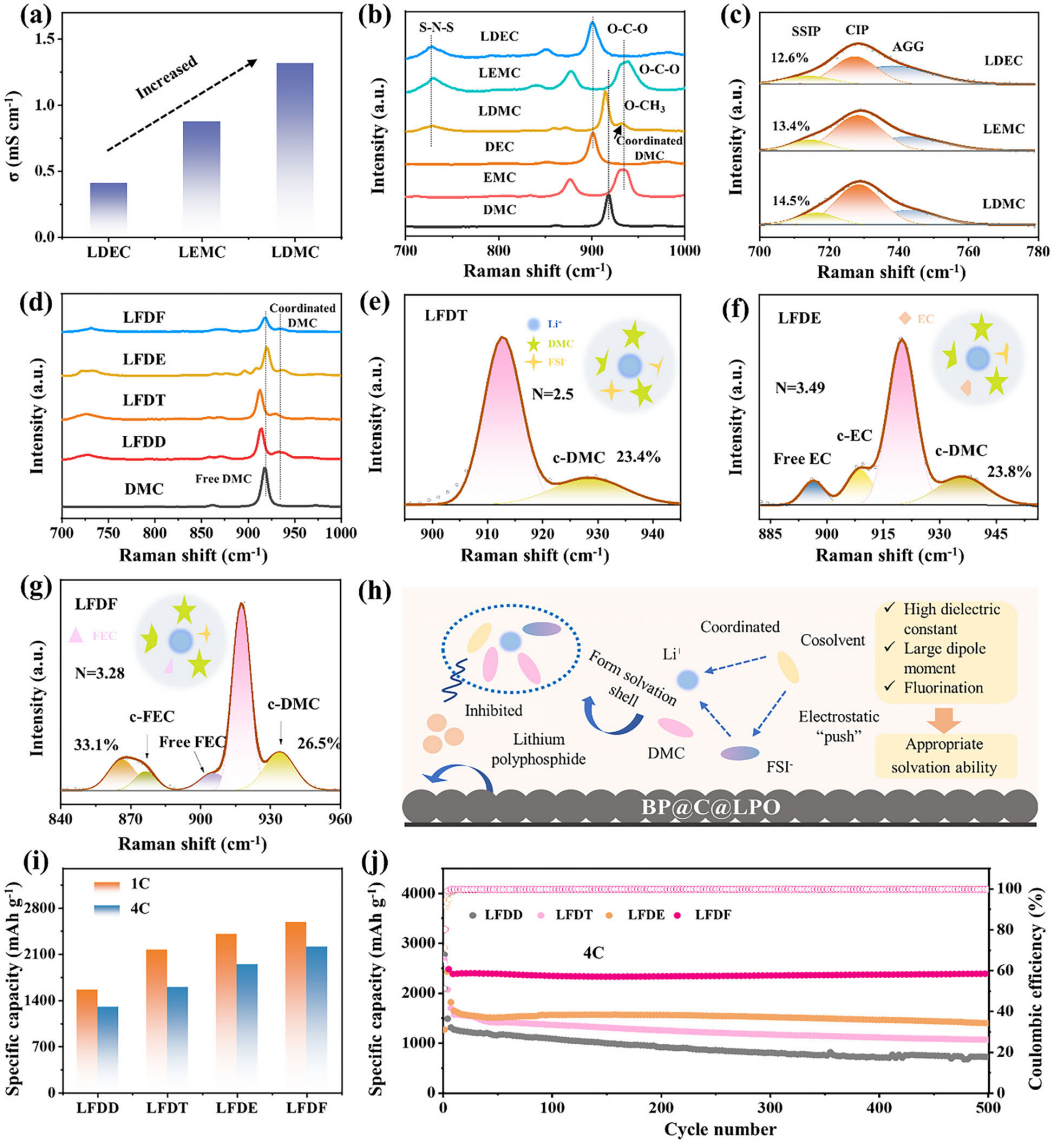
Characterization and performance of WSEs. (a) Ionic conductivity of LDEC, LEMC, and LDMC electrolytes; (b) Raman spectra of the corresponding solvents and electrolytes; (c) Deconvoluted Raman spectra of LDMC, LEMC, and LDEC in the range of 700–780 cm⁻¹; (d) Comparative Raman spectra of LFDD, LFDT, LFDE, and LFDF; (e–g) Deconvoluted Raman spectra of LFDT, LFDE, and LFDF, highlighting coordinated solvent molecules (c‐DMC and c‐FEC); (h) Schematic of the design principles for WSEs; (i) Specific capacity and (j) cycling stability of a BP@C@LPO electrode in LFDD, LFDT, LFDE, and LFDF electrolytes.

In this study, we designed and synthesized a phosphorus‐based anode, denoted as BP@C@LPO, comprising 40 wt.% BP, 50 wt.% conductive carbon (C), and 10 wt.% lithium phosphate (LPO). This composite was prepared via ball milling, with detailed structural and morphological characterizations provided in the Supplementary Information (Figures S1–S7, Supporting Information). To optimize electrolyte performance, LiFSI was selected due to its high ionic conductivity, thermal stability, and hydrolytic resistance. Three WSEs with 1 м LiFSI were made using linear carbonates with a low dielectric constant and poor solvated power: DMC, EMC (ethyl methyl carbonate), and DEC (diethyl carbonate), named LDMC, LEMC, and LDEC. Among these, LDMC exhibited the highest ionic conductivity (**Figure** **1**a) and demonstrated superior high‐rate performance (Figure S8, Supporting Information). In contrast, LDEC displayed inadequate ionic conductivity, rendering it ineffective for stable battery operation. To elucidate solvent‐dependent Li⁺ solvation structures, Raman spectroscopy was employed to probe molecular interactions within the electrolytes. The Raman spectra revealed distinct vibrational modes corresponding to the O─CH_3_ stretching in DMC (919.8 cm⁻¹),^[^ ^11^ ^]^ O─C─O bending in EMC (934.3 cm⁻¹)^[^ ^12^ ^]^ and O─C─O bending in DEC (901.8 cm⁻¹)^[^ ^13^ ^]^ (Figure 1b). Notably, only LDMC exhibited a pronounced coordination peak with a significant spectral shift, indicative of its superior solvation ability. The redshift of the S─N─S bending mode of FSI⁻ from 771 to 728 cm⁻¹ demonstrated the dissociation of Li⁺–FSI⁻ interactions.^[^ ^9^, ^14^ ^]^ Deconvolution of the solvation environment revealed characteristic peaks at 716, 729, and 746 cm⁻¹, corresponding to solvent‐separated ion pairs (SSIPs), contact ion pairs (CIPs), and ion aggregates (AGGs), respectively (Figure 1c). Among the electrolytes studied, LDMC exhibited the highest proportion of SSIPs (14.5%), indicating the most effective LiFSI dissociation and enhanced ionic transport.^[^ ^15^ ^]^ These findings highlight the critical role of solvent selection in tailoring electrolyte solvation structures for improved electrochemical performance in phosphorus‐based anodes.

Raman spectral fitting analysis revealed that the LDMC electrolyte contained 17% coordinated DMC, a higher fraction than the coordinated EMC and DEC in the LEMC and LDEC electrolytes, respectively (Figure S9, Supporting Information). The calculated coordination numbers for DMC, EMC, and DEC were 2.15, 1.46, and 0.69, respectively, correlating with their dielectric constants of 3.1, 3.0, and 2.8 (Table S1, Supporting Information).^[^ ^16^ ^]^ These findings indicate that solvents with higher dielectric constants facilitate increased ionic conductivity, enhance solvent coordination numbers, and promote the formation of well‐defined solvation structures, thereby improving the overall solvation capability of the electrolyte. As a result, the weakly solvated LDMC electrolyte, distinguished by its superior solvation properties and high ionic conductivity, outperforms the other tested WSEs.

To enhance the dissociation ability of the LDMC electrolyte, cosolvents with higher dielectric constants than DMC were selected, including DOL (*ɛ* = 7.1), TEP (*ɛ* = 13.1), FEC (*ɛ* = 78.4) and EC (*ɛ* = 89.8) (Table S1, Supporting Information).^[^ ^16^, ^17^ ^]^ Raman spectroscopy was used to elucidate the interactions between DMC and these cosolvents. The introduction of DOL as a cosolvent induced a slight redshift in the O─CH_3_ stretching mode of DMC (Figure S10a, Supporting Information). Similar spectral shifts were observed in mixtures of DMC with TEP, EC, and FEC (Figure S10b–d, Supporting Information), suggesting intermolecular interactions between DMC and the cosolvents.^[^ ^18^ ^]^ Additionally, distinctive shifts in the Raman spectra of DMC–EC and DMC–FEC mixtures were attributed to the ring‐breathing mode of EC and the C─F stretching vibrations of FEC, respectively, indicating strong solvent–cosolvent interactions.^[^ ^19^ ^]^ To further evaluate the solvation capabilities, single‐cosolvent electrolytes containing 1 м LiFSI were prepared using DOL, TEP, EC, and FEC, designated as LFD, LFT, LFE, and LFF, respectively. The Raman results of single‐cosolvent electrolytes in Figures S11 and S12, (Supporting Information) establish a strong correlation between solvation ability and the dielectric constants of the cosolvent, as previously discussed, further validating their role in optimizing electrolyte performance.

To elucidate the influence of different cosolvents on electrolyte performance, four WSEs were formulated using DOL, TEP, EC, and FEC as cosolvents (1 м LiFSI DMC: cosolvent = 9:1 by volume), denoted as LFDD, LFDT, LFDE, and LFDF, respectively. Raman spectroscopy revealed a pronounced redshift in the free DMC signal for LFDD and LFDT, indicative of enhanced solvent–solute interactions (Figure 1d). Given the superior solvation abilities of EC and FEC, the solvent coordination numbers in LFDE and LFDF were calculated to be 3.49 and 3.28, respectively, higher than 2.36 and 2.5 in LFDD and LFDT (Figure 1e–g and Figure S13, Supporting Information). This higher coordination in LFDE and LFDF suggests enhanced salt dissociation and improved ionic conductivity (Figure S14, Supporting Information), thereby facilitating high‐rate electrochemical performance. Notably, the coordination number of FSI⁻ anions in LFDF was 0.72, exceeding the 0.51 observed in LFDE, which is attributed to FEC's higher dipole moment than that of EC (Table S1, Supporting Information). The incorporation of FEC into LFDF significantly enhances FSI⁻ coordination, thereby promoting the formation of an inorganic‐rich SEI composed of lithium nitride (Li_3_N) and LiF.

As summarized in Figure 1h, the ideal cosolvent in WSEs should effectively coordinate with Li^+^, generate an electrostatic “push” with the FSI⁻ ion, and inhibit LiPPs upon solvation shell formation. These insights establish three key principles for designing high‐performance WSEs: 1) a high dielectric constant to enhance solvation ability, 2) a significant dipole moment to speed coordination dynamics, and 3) fluorination to facilitate FSI⁻ anion incorporation. Collectively, these factors suppress the dissolution of LiPPs while improving charge‐transfer kinetics. To validate these principles, the electrochemical stability of the four WSEs was evaluated using a BP@C@LPO anode. The LFDF electrolyte exhibited the highest capacity, delivering 2586.6 mAh g⁻¹ at 1C and 2210.7 mAh g⁻¹ at 4C (Figure S15, Supporting Information; Figure 1i), while maintaining nearly 100% capacity retention after 500 cycles at 4C. In contrast, the other electrolytes exhibited lower capacities and inferior retention (Figure 1j). This performance directly aligns with the proposed design criteria. Further supporting the rule, the influence of different FEC concentrations (10, 30, and 50 vol.%) on the electrolyte solvation structure and electrochemical performance was systematically investigated, which suggests that an optimal coordination number is critical for superior electrochemical performance (Figures S16 and S17 and Table S2, Supporting Information). Taken together, these results validate the proposed design principles, establishing a foundational strategy for developing next‐generation electrolytes optimized for fast‐charging phosphorus‐based anodes.

### 2.2 Electronic Structure and Interface Interaction

**Figure 2:**
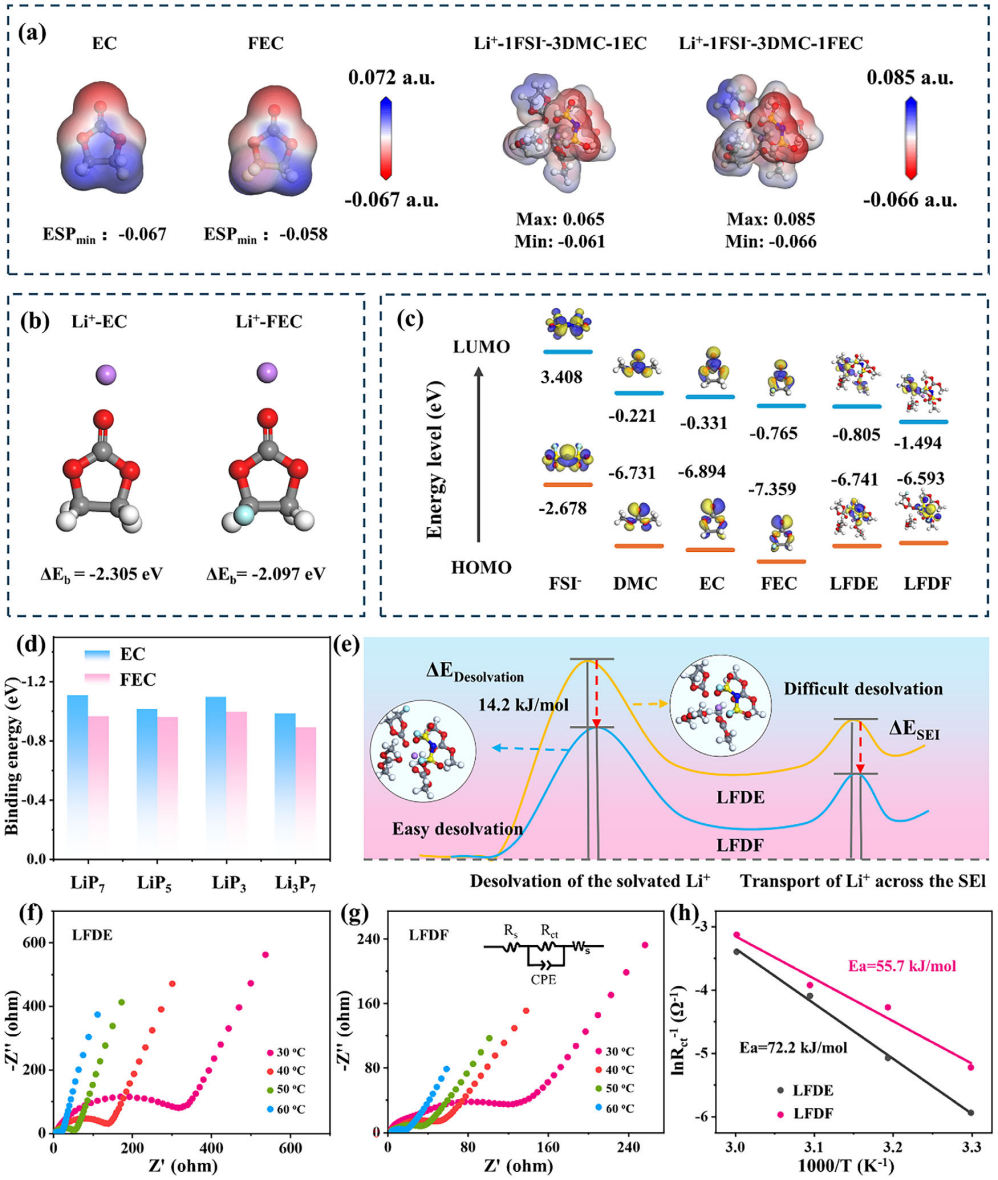
Theoretical calculation and measurement of the desolvation activation energy of LFDE and LFDF. a) Electrostatic potential mappings of EC, FEC, Li^+^‐1FSI^−^‐3DMC‐1EC and Li^+^‐1FSI^−^‐3DMC‐1FEC based on the electron density; (b) Binding energy of Li^+^‐EC and Li^+^‐FEC; (c) Schematic of the LUMO/HOMO energy level of anion, solvents and electrolyte; (d) Adsorption energy of EC and FEC with LiP_7_/LiP_5_/LiP_3_/Li_3_P_7_; (e) Schematic of the enhanced kinetics enabled by LFDF; (f, g) Electrochemical impedance spectroscopy (EIS) curves of BP@C@LPO at various temperatures after one activation cycle in LFDE and LFDF; (h) Desolvation activation energy derived from the Arrhenius equation.

Compared to DOL and TEP, EC and FEC as cosolvents exhibit superior electrochemical performance, necessitating further mechanistic investigations to elucidate the effect of fluorination. To assess solvation ability, electrostatic potential (ESP) calculations were performed. As shown in **Figure** **2**a, EC exhibits a lower ESP_min_ value, indicating stronger solvation ability. In contrast, the fluorine substitution in FEC exerts an electron‐withdrawing effect, reducing the negative charge on carbonate oxygen to −0.451 e and increasing the ESP_min_ value (Figures S18–S21, Supporting Information). This weaker solvation ability favors the formation of an SEI layer enriched with inorganic components. Further insights were gained by comparing the electron distribution of Li⁺‐1FSI⁻‐3DMC‐1EC and Li⁺‐1FSI⁻‐3DMC‐1FEC complexes. The ESP_max_ of the latter is significantly higher, suggesting larger polarization and stronger coulombic interactions with the positive electrode (Figure 2a). This enhanced interaction reduces resistance during ion migration, thereby improving ionic mobility.^[^ ^17^, ^20^ ^]^ Additionally, Li⁺‐FEC (−2.097 eV) exhibits a weaker binding energy than Li⁺‐EC (−2.305 eV) (Figure 2b), corroborating its reduced solvation capability. The binding energy of Li⁺‐DMC is −2.069 eV, whereas that of Li⁺‐FSI⁻ is −6.210 eV (Figure S22, Supporting Information), indicating that a higher fraction of anions is incorporated into the solvation sheath, which promotes the formation of a WSE. To evaluate redox stability, the highest occupied molecular orbital (HOMO) and lowest unoccupied molecular orbital (LUMO) energies were calculated for EC‐ and FEC‐based electrolytes. As depicted in Figure 2c, LFDF exhibits the lowest LUMO energy level, favoring electrolyte reduction and the formation of a stable SEI.^[^ ^21^ ^]^ Linear sweep voltammetry further validated these findings (Figure S23, Supporting Information). These results collectively demonstrate that fluorination facilitates the formation of an anion‐dominated solvation structure, enhancing SEI robustness and electrolyte stability.

Furthermore, the WSE with FEC as a cosolvent effectively mitigates the dissolution of LiPPs. As shown in Figure 2d, Figures S24 and S25, (Supporting Information), the adsorption energy of LiPPs in FEC is significantly lower than in EC, indicating that FEC more effectively suppresses the dissolution of LiPPs. The reduced LiPPs dissolution enhances lithium‐ion (Li^+^) transport, thereby improving the conversion kinetics among various phosphorus species. In fast‐charging LIBs, Li^+^ desolvation is often the rate‐limiting step.^[^ ^22^ ^]^ As presented in Figure 2e and Figure S26, (Supporting Information), LFDF exhibits a lower solvation energy by 14.2 kJ mol⁻¹ than LFDE, along with a reduced desolvation energy, as shown in Figure S27, (Supporting Information). These findings suggest that WSE with a fluorinated cosolvent facilitates Li^+^ desolvation.^[^ ^23^ ^]^ Furthermore, activation energy derived from the Arrhenius equation (Figure 2f–h) reveals that the BP@C@LPO anode with LFDF electrolyte exhibits a significantly lower reaction activation energy of 55.7 kJ mol⁻¹ than 72.2 kJ mol⁻¹ in LFDE. This substantial reduction in activation energy confirms that fluorination effectively accelerates the reaction kinetics of the BP@C@LPO anode.

### 2.3 Reaction Kinetics and Suppression of LiPPs Dissolution

**Figure 3:**
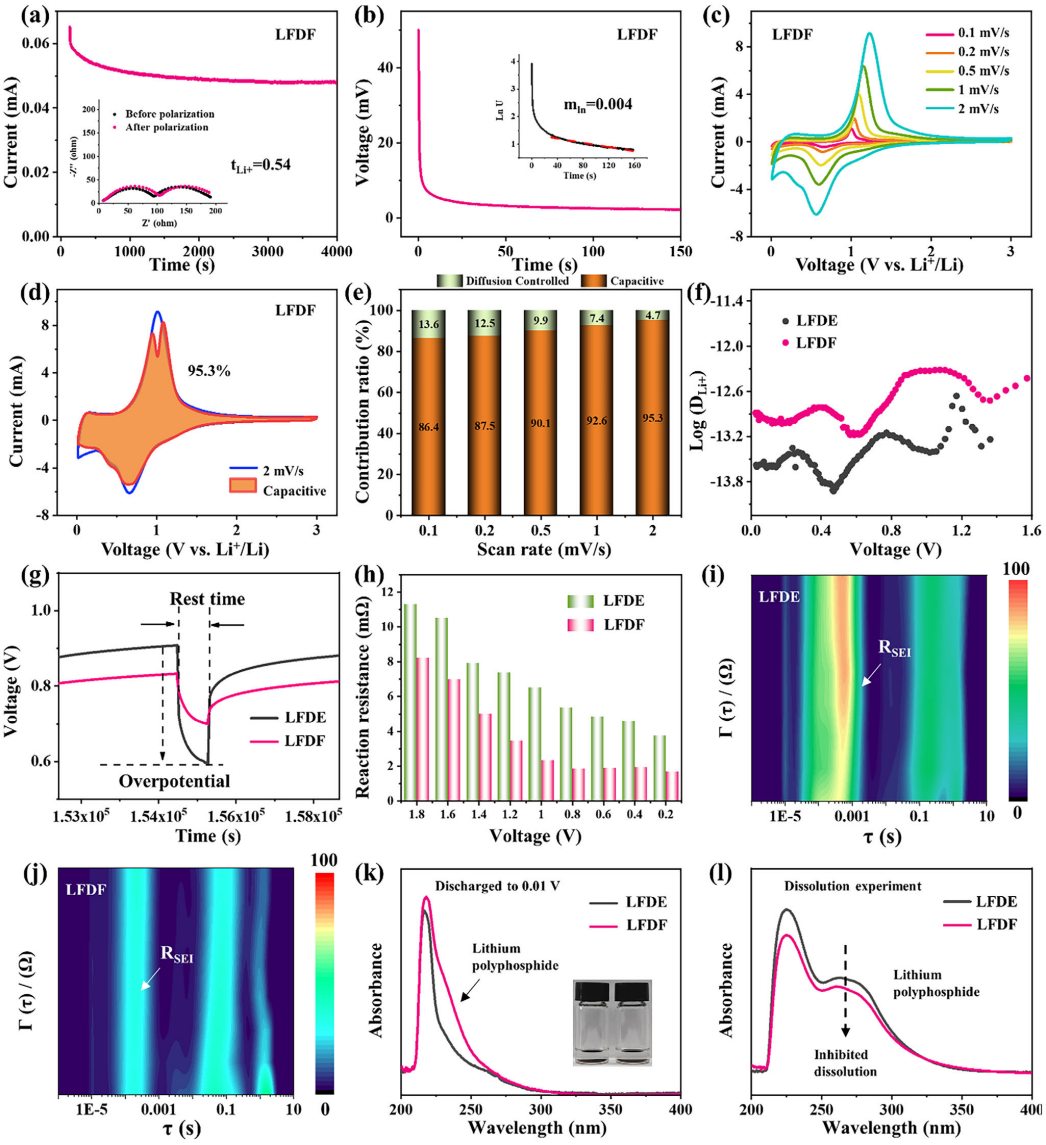
Reaction kinetics and suppression of dissolution of lithium polyphosphides in LFDE and LFDF. a) Chronoamperometry profile of LFDF at 10 mV polarization. Inset: EIS results; (b) Galvanostatic pulse polarization of LFDF; (c) Cyclic voltammetry (CV) curves of the BP@C@LPO in LFDF at various scan rates; (d) Percentage of pseudocapacitive contribution in the BP@C@LPO at 2 mV s^−1^ in LFDF. e) Contribution ratios of diffusion‐controlled and pseudocapacitive currents of BP@C@LPO in LFDF; (f) Li⁺ diffusion coefficients of BP@C@LPO in LFDE and LFDF; (g) Enlarged potential response curves; (h) Reaction resistances from GITT measurements; (i, j) DRT analysis of BP@C@LPO in (i) LFDE and (j) LFDF; (k) Ultraviolet–visible spectra (UV–visible spectra) and optical images of the BP electrode discharged to 0.01 V in LFDE and LFDF, with DEC as a soaking solvent; (l) UV–vis spectra of lithium polyphosphides dissolved in LFDE and LFDF.

To further investigate the influence of fluorinated cosolvents in WSEs on Li⁺ transport capabilities, a comparative analysis was conducted. Specifically, Sand's time (*T*_sand_) for LFDE and LFDF electrolytes was calculated using a well‐established classical equation.^[^ ^24^ ^]^ As shown in Figure 3a and Figure S28a, (Supporting Information), the Li^+^ transfer number of LFDF electrolytes is 0.54, higher than the 0.41 observed for LFDE, highlighting the stronger Li^+^ migration ability of LFDF.^[^ ^25^ ^]^ Based on the parameter m_ln_, derived from the slope of the Napierian logarithm of relaxation potential versus relaxation time (Figure 3b and Figure S28b, Supporting Information), the Li⁺ diffusion coefficients ($D_{Li^{+}}$) for LFDE and LFDF electrolytes were calculated to be 1.19×10^−7^ and 1.53×10^−7^ cm^2^ s^−1^, respectively. The larger value of $D_{Li^{+}}$ in LFDF confirms a faster diffusion rate, ensuring improved charge‐discharge performance. Moreover, the T_sand_ value for LFDF is 3214.7 s, significantly longer than the 2514.3 s observed for LFDE, further substantiating the enhanced Li⁺ transport capability imparted by WSEs with a fluorinated cosolvent.^[^ ^26^ ^]^

Reaction kinetics analysis highlights the benefits of fluorination and electrolyte‐electrode compatibility. Pseudocapacitance contributions were slightly higher in the BP@C@LPO with LFDF than with LFDE (Figure 3c–e, Figure S29, Supporting Information), indicating superior fast‐charging capability facilitated by the WSE with FEC. Galvanostatic intermittent titration technique measurements (Figure S30, Supporting Information, **Figure** **3**f) revealed higher Li⁺ diffusion coefficients and lower overpotentials in the BP@C@LPO with LFDF during lithiation, demonstrating improved interfacial ion transport (Figure 3g).^[^ ^27^ ^]^ Reaction resistance was consistently lower in the BP@C@LPO with LFDF across charge‐discharge cycles (Figure 3h), confirming reduced redox overpotential.^[^ ^28^ ^]^ In situ EIS analysis (Figure S31, Supporting Information and Figure 3i,j) further confirms the rapid reaction kinetics of WSE using FEC as a cosolvent. In the distribution of relaxation times (DRT) plot, the peak observed at a frequency of approximately 10^3^ Hz, (*τ*_1_), associated with the resistance of solid‐electrolyte interphase (*R*_SEI_), confirms SEI formation, while the peak at around 10 Hz (τ_2_) corresponds to the resistance of charge transfer resistance (*R*_ct_).^[^ ^29^ ^]^ As shown in Figure 3i,j, both *R_SEI_* and *R*_ct_ exhibit lower intensities in LFDF, highlighting the crucial role of fluorination in facilitating charge transfer and forming a robust, high‐conductivity SEI layer.^[^ ^30^ ^]^

The WSE with a fluorinated cosolvent not only enhances Li⁺ transport and reaction kinetics but also suppresses the dissolution of LiPPs while promoting their conversion. The BP electrode was first discharged to 0.01 V in both LFDE and LFDF electrolytes and then immersed in DEC overnight for full dissolution of discharged products. The UV–vis spectroscopy of the resultant solution (Figure 3k) reveals higher LiPPs concentrations in LFDF, including Li_3_P, indicating higher phosphorus conversion. To evaluate dissolution, LiPPs were added to the electrolytes, showing lower peak intensities in LFDF compared with LFDE (Figure 3l), confirming reduced solubility of fluorinated WSE. Solubility tests in EC and FEC (Figure S32, Supporting Information) further demonstrated FEC's minimal dissolution, aligning with electrolyte performance trends. These results highlight the dual role of fluorination in mitigating dissolution and facilitating efficient phosphorus conversion.

### 2.4 Electrochemical Performances of BP@C@LPO in LFDE and LFDF

**Figure 4:**
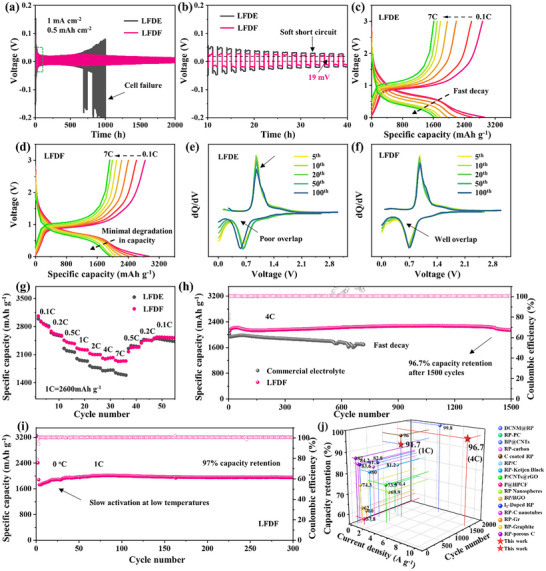
Electrochemical performance of BP@C@LPO in LFDE and LFDF. (a) Cycling stability of Li||Li symmetric cells at 1 mA cm⁻^2^ (0.5 mAh cm⁻^2^); (b) Enlarged view of the voltage profiles in (a). c,d) Charge/discharge curves of the BP@C@LPO at 0.1C to 7C in (c) LFDE and (d) LFDF; (e, f) *dQ/dV* profiles of the BP@C@LPO at 1C in (e) LFDE and (f) LFDF electrolytes. g) Rate capability comparison; (h) High‐rate cycling performance of BP@C@LPO at 4C in LFDF and a commercial electrolyte (1 м LiPF_6_ EC: DEC = 1:1 10 wt.% FEC+1 wt.% VC); (i) Cycling performance of BP@C@LPO at 1C under 0 °C in LFDF; (j) Electrochemical performance comparison with previously reported phosphorus‐based anodes.

Li||Li symmetric cell tests revealed LFDF's superior interfacial stability, with minimal polarization (19 mV) and stable operation for 2000 h, while LFDE showed higher polarization, voltage fluctuations, and eventual soft short circuits (**Figure** **4**a,b). This confirms that LFDF promotes uniform Li⁺ flux and lithium deposition, enhancing cycle life.^[^ ^31^ ^]^ Subsequently, the electrochemical performance of LFDE and LFDF electrolytes was evaluated in cells with BP@C@LPO electrodes. LFDF and LFDE performed similarly at low rates (Figure S33, Supporting Information), but LFDF maintained stable capacity retention at high rates, while LFDE suffered significant capacity loss (Figure 4c,d). Consistent differential capacity curve (*dQ/dV*) profiles over 100 cycles (Figure 4e,f) and CV analysis (Figure S34, Supporting Information) further validated LFDF's stability. These results demonstrate that the WSE with a fluorinated cosolvent ensures excellent interfacial stability and highly efficient Li⁺ transport, making it well‐suited for phosphorus‐based anodes.

A comprehensive comparison of the fast‐charging capabilities of LFDE and LFDF electrolytes is presented in Figure 4g. Within the 0.5C to 7C range, significant differences in capacity retention are observed. Notably, at 7C, the LFDF‐based cell retains 69% of its initial capacity, whereas the one with LFDE maintains only 56%, highlighting the superior rate performance of LFDF. When tested at 1C, the LFDF‐based cell achieves an impressive specific capacity of 2615.2 mAh g⁻¹, coupled with a high capacity retention of 91.7% after 500 cycles, significantly outperforming the LFDE‐based one (Figure S35, Supporting Information). Furthermore, at 4C, LFDF‐based cell maintains a high capacity with an exceptional retention rate of 96.7% after 1500 cycles (Figure 4h). Conversely, the LFDE‐based cell delivers a lower capacity (Figure 1j), and the commercial electrolyte counterpart undergoes rapid degradation after 720 cycles. When increasing the areal capacity to 3 mAh cm^−2^, the LFDF‐based cell still delivers a capacity of 2555.1 mAh g^−1^ at 0.5C, significantly outperforming the 2183.5 mAh g^−1^ achieved with commercial electrolyte (Figure S36, Supporting Information). These results clearly demonstrate that the LFDF electrolyte enables excellent high‐rate capability while supporting high mass loading, making it particularly suitable for practical high‐energy‐density battery applications. Additionally, the LFDF electrolyte demonstrates outstanding low‐temperature performance, achieving 97% capacity retention after 300 cycles at 0 °C at 1C, with a specific capacity of 1956.2 mAh g⁻¹ (Figure 4i). When compared to previously reported phosphorus‐based anodes and electrolytes, the BP@C@LPO electrode with LFDF exhibits superior electrochemical performance (Figure 4j and Tables S3 and S4, Supporting Information). Beyond its promising half‐cell performance, the LFP//BP@C@LPO full cell, assembled with a lithium iron phosphate (LiFePO_4_) cathode and LFDF electrolyte, delivers a remarkable specific capacity of 154.5 mAh g⁻¹, maintaining 92.4% capacity retention after 100 cycles (Figures S37 and S38, Supporting Information). Even under 4C, the LFP//BP@C@LPO full cell employing the LFDF electrolyte demonstrated a capacity of 78.9 mAh g^−1^ and enabled 87.6% capacity retention after 300 cycles (Figure S39, Supporting Information). The compatibility of the electrolytes with the NCM811 cathodes of higher voltages indicates that this electrolyte system is suitable for low‐voltage fast‐charging systems (Figures S40 and S41, Supporting Information). These results underscore the effectiveness of the WSE with a fluorinated cosolvent in enhancing reaction kinetics, suppressing the dissolution of LiPPs, and stabilizing the electrolyte, positioning it as an outstanding candidate for fast‐charging applications in low‐voltage systems.

### 2.5 Analysis of Structural and Surface Compositions After Discharged to 0.01 V

To further investigate the electrode‐electrolyte interactions and stability, post‐cycling characterizations were performed. Ex situ X‐ray diffraction (XRD) analysis was conducted to evaluate the reversibility of the conversion reaction. As shown in Figure S42 (Supporting Information), after the initial charge‐discharge cycles, peaks corresponding to LiP_5_ and Li_3_P_7_ are detected in the BP@C@LPO electrode with LFDE electrolyte. In contrast, no additional crystalline phases are observed in the electrode cycled with LFDF electrolyte, suggesting that the WSE with a fluorinated cosolvent facilitates the efficient and reversible conversion of phosphorus species within the electrode.

**Figure 5:**
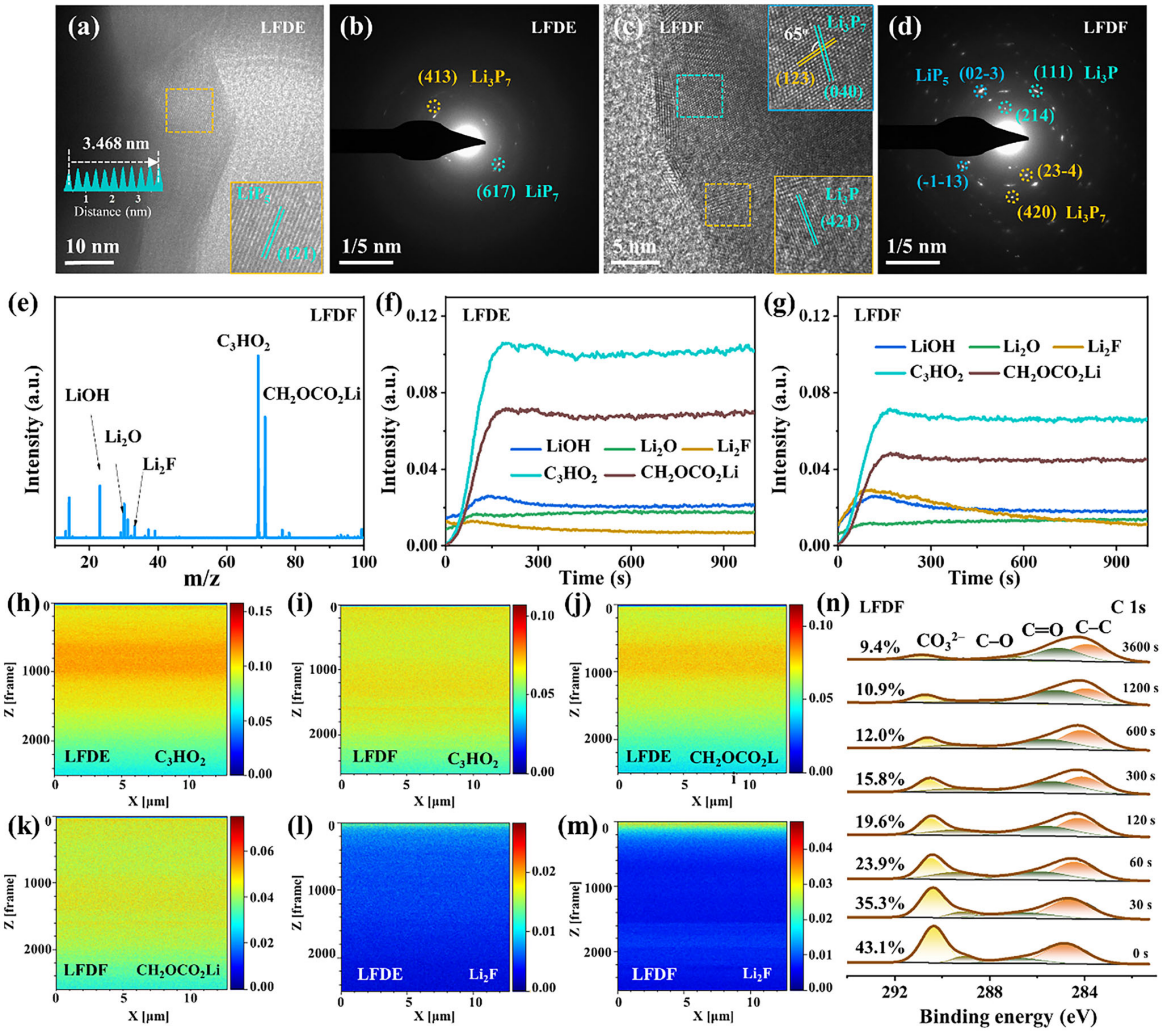
Structural evolution and interfacial chemistry in BP@C@LPO electrodes after discharged to 0.01 V. TEM images and corresponding SAED patterns of a BP@C@LPO in (a, b) LFDE and (c, d) LFDF; (e) Mass spectrum of a BP@C@LPO in LFDF collected in a positive ion mode. (f, g) FIB‐SIMS depth profiling curves of a BP@C@LPO obtained in a positive ion mode in LFDE and LFDF; (h–m) Mapping distributions of discharged fragments (0.01 V) of a BP@C@LPO electrode in LFDE and LFDF; (n) C 1s XPS spectra of a BP@C@LPO electrode in LFDF at different etching times.

Transmission electron microscopy (TEM) was employed to investigate the reaction products of the BP@C@LPO electrode after being discharged to 0.01 V in different electrolytes, as shown in Figure S43, (Supporting Information) and **Figure** **5**a–d. The BP@C@LPO electrode in the LFDE electrolyte shows LiP_5_ and Li_3_P_7_/LiP_7_ phases, with no detectable Li_3_P (Figure 5a,b). In contrast, when the LFDF electrolyte was used, the BP@C@LPO electrode exhibited a complete conversion product of Li_3_P (Figure 5c,d and Figure S43c,d, Supporting Information), aligning well with XRD analysis. These findings confirm that the WSE with a fluorinated cosolvent promotes the complete conversion reaction of phosphorus, thereby accelerating reaction kinetics.

To analyze the SEI composition, focused ion beam‐secondary ion mass spectrometry (FIB‐SIMS) was performed on the BP@C@LPO electrode discharged to 0.01 V in different electrolytes. As shown in Figure S44 (Supporting Information), the sputtering depth was about 4 µm. The mass spectrum in a positive ion mode (Figure 5e and Figure S45, Supporting Information) shows key SEI components, including LiOH, Li_2_O, Li_2_F, C_3_HO_2_, and CH_2_OCO_2_Li. Depth profiles in a positive ion mode (Figure 5f,g) reveal differences in SEI composition between LFDF and LFDE. Specifically, the organic components of C_3_HO_2_ and CH_2_OCO_2_Li give weaker signals in LFDF, while Li_2_F intensity is stronger. This suggests that the WSE with FEC as a cosolvent promotes a LiF‐rich SEI, enhancing interfacial stability. Mapping images (Figure 5h–m) show that the BP@C@LPO electrode with LFDE has more organic components on the surface and less lithium fluoride. In contrast, the BP@C@LPO electrode with LFDF had organic components mainly in the outer SEI layer and inorganic Li_2_F in the inner layer. These findings highlight the role of the fluorinated cosolvent in forming a LiF‐rich SEI, which improves the stability and performance of phosphorus‐based anodes.

X‐ray photoelectron spectroscopy (XPS) depth profiling of the SEI layer (Figure 5n, Figures S46–S48, Supporting Information) revealed distinct compositional trends. Peaks for C─C (284.8 eV), C─O (286.6 eV), C═O (288.0 eV), and CO_3_^2^⁻ (290.1 eV) were observed,^[^ ^32^ ^]^ but CO_3_^2^⁻ become diminishing with etching depth, confirming its surface dominance. Compared to LFDE, BP@C@LPO in LFDF exhibited a higher Li_2_CO_3_ content and elevated elemental phosphorus but reduced phosphate ions, indicating that side reactions were suppressed (Figure S46a, Supporting Information). Persistent S‐F/FSI signals in LFDF after etching for 1200 s (Figure S46b, Supporting Information) suggest a dense, protective SEI that effectively protects the electrode surface. Additionally, Li 1s, N 1s, and S 2p spectra (Figure S48, Supporting Information) reveal the involvement of the LiFSI component in the reaction. XPS depth profiling demonstrates that the WSE with a fluorinated cosolvent facilitates the incorporation of anions into the solvation sheath, leading to the formation of an SEI layer with outer organic components and inner inorganic species.

### 2.6 Surface Compositional Analysis after 200 Cycles

**Figure 6:**
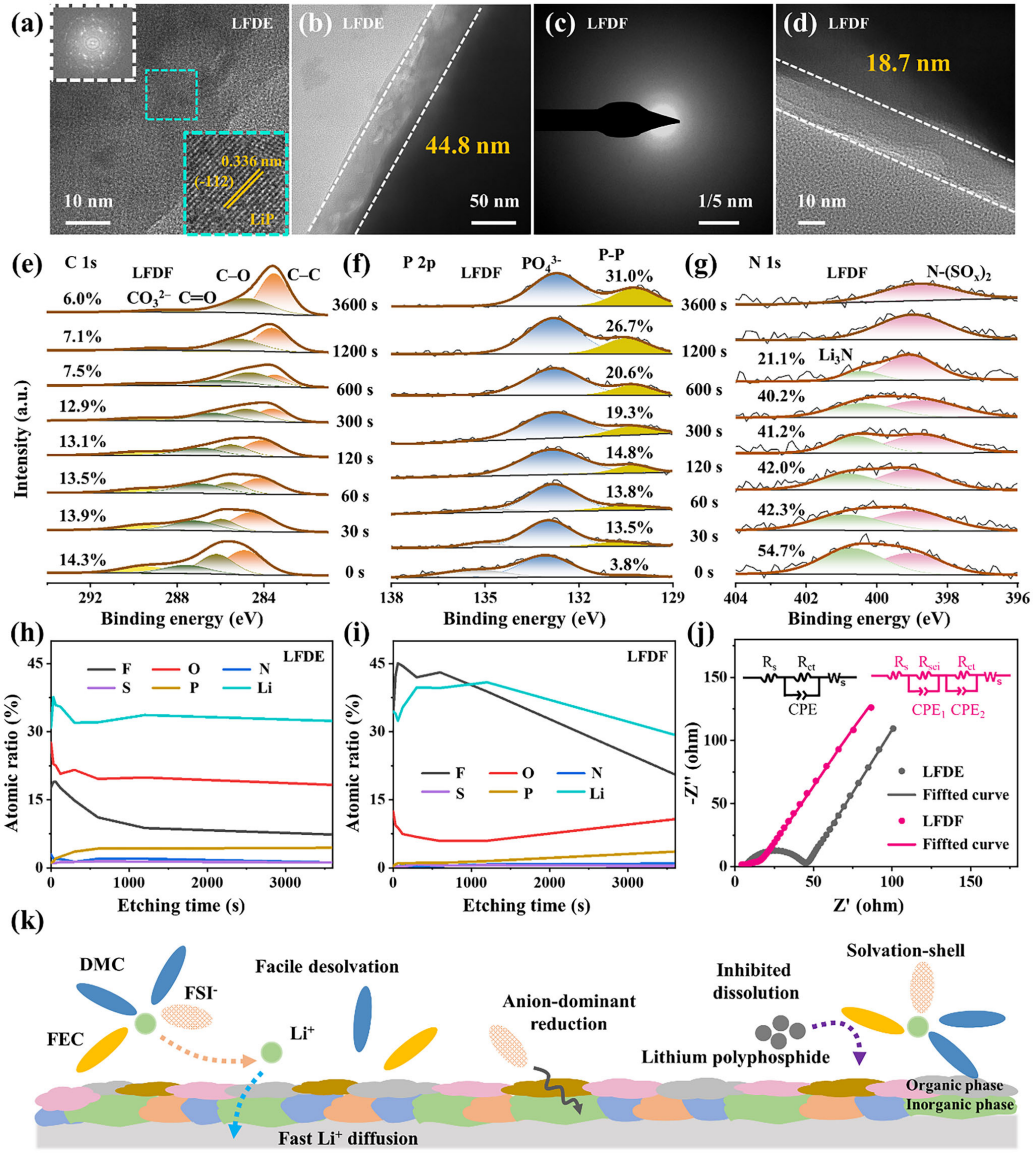
SEI analysis after 200 cycles. a,b) TEM images of the BP@C@LPO in LFDE after 200 cycles. c,d) SAED patterns and TEM image of the BP@C@LPO in LFDF after 200 cycles. e) C 1s, (f) P 2p, and (g) N 1s spectra of BP@C@LPO in LFDE. h,i) Atomic ratios of elements of the SEI formed in different electrolytes at various sputtering times. j) EIS spectra of BP@C@LPO after 200 cycles in LFDE and LFDF. k) Schematic of the mechanism of WSE using a fluorinated cosolvent.

To evaluate the long‐term stability and effectiveness of the SEI layer, we conducted a series of characterizations after 200 cycles. Energy‐dispersive X‐ray spectroscopy mapping showed pronounced S/F signals and significant phosphorus loss in a BP@C@LPO electrode in LFDE, indicating severe electrolyte decomposition and active material dissolution (Figures S49–S51, Supporting Information). These findings suggest that the WSE with a fluorinated cosolvent effectively mitigates the dissolution of LiPPs and suppresses detrimental side reactions. Correspondingly, the electrode in LFDE exhibited 49.3% volumetric expansion versus 21.2% for LFDF counterparts, indicating that the robust SEI formed in the electrode with LFDF helps alleviate volume expansion (Figure S52, Supporting Information). TEM analysis revealed intermediate products like LiP in the electrode with LFDE, indicating irreversible reactions, while the one with LFDF maintained its amorphous structure, showing rapid and complete reaction kinetics (Figure S53, Supporting Information and **Figure** **6**a–d). The BP@C@LPO electrode with LFDE formed a porous and thick SEI, which allows easier penetration of electrolyte components, potentially leading to continuous side reactions between the electrolyte and electrode. On the other hand, the LFDF electrolyte promoted the formation of a dense and robust SEI layer that effectively protects the electrode while enabling efficient Li^+^ transport. This dual functionality of the LFDF electrolyte enhances the rate capability and cycle life of the electrode.

XPS was employed to analyze the components of the SEI. In the C 1s spectrum (Figure 6e and Figure S54a, Supporting Information), Li_2_CO_3_ was detected in both samples after 200 cycles. As the etching depth was increased, the Li_2_CO_3_ content remained relatively stable, indicating the formation of a dense SEI in the LFDF electrolyte. Higher elemental P and lower phosphate ions in the LFDF‐based BP@C@LPO electrode (Figure 6f, Figure S54b, Supporting Information) confirm suppressed side reactions. In the N1s spectrum (Figure 6g and Figure S54c, Supporting Information), peaks at 400.7 and 398.9 eV were assigned to Li_3_N and N‐(SO_x_)_2_, respectively.^[^ ^33^ ^]^ The higher Li_3_N content in the LFDF electrolyte suggests enhanced SEI conductivity. The S 2p and F 1s spectra (Figure S55, Supporting Information) further confirm the presence of inorganic components within the SEI. Atomic ratio analysis at various sputtering depths (Figure 6h,i) reveals that the electrode in LFDF has a lower oxygen content and higher fluorine content, indicating a SEI with a higher inorganic and lower organic content. Additionally, the gradual decrease in F and Li signals with increased etching time in the electrode in LFDF suggests a uniform SEI primarily composed of inorganic LiF components. EIS after 200 cycles confirmed the superior conductivity of the SEI in LFDF with the R_ct_ of the BP@C@LPO at only 7 Ω, while it was 36 Ω in LFDE (Figure 6j). From its initial formation to 200 cycles, the SEI in LFDF retains high conductivity and compactness, indicating that the WSE with a fluorinated co‐solvent facilitates the formation of an SEI with high conductivity and a uniform, inorganic‐rich composition.^[^ ^34^ ^]^

FEC, serving as a fluorinated cosolvent in the WSE, offers several advantages over non‐fluorinated EC, as illustrated in Figure 6k. The fluorinated cosolvent is conducive to suppressing the dissolution of LiPPs more effectively for the lower adsorption energy of LiPPs. The reduced polyphosphide dissolution enhances Li^+^ transport and reduces the loss of active material, thereby improving the conversion kinetics among various phosphorus species. Except for the direct interactions with phosphorus‐based anodes, the fluorinated cosolvent in WSEs also greatly enhances interfacial stability and reaction kinetics of the phosphorus‐based anodes. First, FEC exhibits a weaker binding energy with Li⁺ and a lower solvation energy, which promotes efficient desolvation and favorable kinetic behavior, even under low‐temperature conditions. Furthermore, FEC lowers the LUMO of the electrolyte, facilitating the incorporation of more anions into the solvation shell and promoting the formation of a stable, inorganic‐rich SEI layer on the BP@C@LPO anode. Consequently, fluorination plays a pivotal role in designing electrolytes tailored for fast‐charging applications with phosphorus‐based anodes.

## 3 Conclusions

In conclusion, we systematically investigated the role of cosolvents in WSEs for phosphorus‐based anodes. The study reveals that a high dielectric constant, a significant dipole moment, and fluorination are crucial for ensuring optimal solvent coordination while enhancing anion coordination. Based on these criteria, a WSE using FEC as a cosolvent was designed, demonstrating significant advantages in minimizing the dissolution of LiPPs, improving reaction kinetics, enhancing interfacial stability, and reducing irreversible reactions. The phosphorus‐based anode paired with this electrolyte exhibited outstanding electrochemical performance, achieving a specific capacity of 2615.2 mAh g⁻¹ at 1C, with 91.7% retention after 1000 cycles. At a higher rate of 4C, it maintained a capacity of 2210.7 mAh g⁻¹, with a remarkable 96.7% retention after 1500 cycles. Even at 0 °C, the anode retained a capacity of 1956.2 mAh g⁻¹ after 300 cycles. This innovative approach offers a promising strategy for phosphorus‐based anodes, enabling rapid charging, excellent cycling stability, and low‐temperature adaptability, making it a valuable advancement for next‐generation energy storage applications.

## Conflict of Interest

The authors declare no conflict of interest.

## Supporting information



Supporting Information

## Data Availability

The data that support the findings of this study are available in the supplementary material of this article.
