# Supplementary material for: Fast‐Charging Phosphorus Anodes Enabled by Fluorinated Weakly Solvated Electrolytes for Stable and High‐Rate Lithium Storage
Source: Adv Mater. 2025 May 7;37(29):2504248. doi: 10.1002/adma.202504248 (PMC12288809; doi:10.1002/adma.202504248)
Supplement: Supplementary file 1 — Supporting Information [file ADMA-37-2504248-s001.docx]

**Supporting Information**

**Fast-Charging Phosphorus Anodes Enabled by Fluorinated Weakly Solvated Electrolytes for Stable and High-Rate Lithium Storage**

Huixian Xie^┼^, Lingwen Liu^┼^, Hongyi Chen^┼^, Kwan San Hui*, Zhuoheng Kuang, Guangmin Zhou, Yuanmiao Sun, Hui-Ming Cheng* and Kwun Nam Hui*

^┼^ These authors contributed equally to this work, * Corresponding authors.

H. Xie, L. Liu, H. Chen, Z. Kuang, Prof. K. N. Hui

Joint Key Laboratory of the Ministry of Education, Institute of Applied Physics and Materials Engineering, University of Macau, Avenida da Universidade, Taipa, Macau SAR, P.R. China

E-mail: bizhui@um.edu.mo

Prof. K. S. Hui

Department of Mechanical Engineering, College of Engineering, Prince Mohammad Bin Fahd University, P.O. Box 1664, Al Khobar, 31952 Kingdom of Saudi Arabia

E-mail: khui@pmu.edu.sa

Prof. G. M. Zhou

Tsinghua Shenzhen International Graduate School, Tsinghua University, Shenzhen 518055, P. R. China

L. Liu, Prof. Y. M. Sun, Prof. H. M. Cheng

Shenzhen Key Laboratory of Energy Materials for Carbon Neutrality, Institute of Technology for Carbon Neutrality, Shenzhen Institutes of Advanced Technology, Chinese Academy of Sciences, Shenzhen 518055, P. R. China

E-mail: hm.cheng@siat.ac.cn

Prof. H. M. Cheng

Faculty of Materials Science and Energy Engineering, Shenzhen University of Advanced Technology, Shenzhen, 518055 P. R. China

Shenyang National Laboratory for Materials Science, Institute of Metal Research, Chinese Academy of Sciences, Shenyang, 110016 P. R. China

**Experimental section**

**Material preparation**

Black phosphorus (BP) was produced by transforming red phosphorus (RP) (Aladdin, 99.99%) through a ball milling process using a FRITSCH Pulverisette-7 planetary ball mill from Germany. The process began with placing 2.2 grams of red phosphorus into an 80 mL ball milling jar, with a steel ball to red phosphorus mass ratio of 40:1. The jar was filled with a 1:1 ratio by count of 10 mm and 5 mm steel balls. To preserve an inert environment, the jar was sealed within a glove box purged with argon gas. After 16 hours of milling at a speed of 800 revolutions per minute (rpm), BP was successfully synthesized.

**Synthesis of BP composites**

In the process of making BP and super P composites, stoichiometric BP accounting for 40 wt.% and super P making up 60 wt.% were mixed in stainless-steel grinding bowls. The mass ratio of steel ball to powder was 70:1 within an argon atmosphere. The ball milling was carried out for 6 hours at a rotational speed of 800 rpm. The resulting sample was labeled as BP@C. Using the same preparation conditions and fixing proportion of black phosphorus (40%), stoichiometric amounts of crystal lithium phosphate (10 and 20 wt.%) (Aladdin, AR, 99%) were added. The composite are named BP@C@LPO and BP@C@LPO(20) respectively.

**Synthesis of the lithium polyphosphide solution**

To prepare lithium polyphosphide, the synthesis started with dissolving 1.54 g of biphenyl (99.5% purity from Macklin) in 10 ml of tetrahydrofuran (THF) (also 99.5% purity from Macklin). Subsequently, 0.01 mol of lithium pieces were added to this solution and the mixture was vigorously stirred to initiate a reaction, leading to the formation of a 1 M solution of lithium biphenyl. After that, 200 mg of red phosphorus was added to the solution, and the reaction was left to complete at room temperature within a glove box. Once the reaction was complete, the solution was centrifuged to separate the solid product. The solid product was then dissolved in diethyl carbonate (DEC) to yield the lithium polyphosphide solution.

**Preparation of electrolyte**

The chemicals of lithium bis(fluorosulfonyl)imide (LiFSI), was purchased from Canrd. Ethyl methyl carbonate (EMC), diethyl carbonate (DEC), dimethyl carbonate (DMC), 1,3-Dioxolane (DOL), triethyl Phosphate (TEP), ethylene carbonate (EC), fluoroethylene carbonate (FEC), and vinylene carbonate (VC) were purchased from Aladdin and dried with 4Å molecular sieves before use. 1 mol lithium salt (LiFSI) was added to the DMC, DEC, EMC to achieve the electrolytes of 1 м LiFSI DMC (LDMC), 1 м LiFSI DEC (LDEC) and 1 м LiFSI EMC (LEMC) respectively. DMC was selected as the main solvent, and DOL, TEP, EC, FEC were added as the cosolvent with the volume ratio of 9:1. The obtained electrolytes of 1 м LiFSI DMC:DOL (9:1 by volume), 1 м LiFSI DMC:TEP (9:1 by volume), 1 м LiFSI DMC:EC (9:1 by volume), 1 м LiFSI DMC:FEC (9:1 by volume) were named as LFDD, LFDT, LFDE and LFDF. To obtain coordination numbers of different solvents, the electrolytes using DOL, TEP, EC and FEC as the single solvent with 1 mol lithium salt (LiFSI) were fabricated, named as LFD, LFT, LFE and LFF. To explore the optimal amount of cosolvent, 1 м LiFSI DMC:FEC (7:3 by volume) (labeled as LFD3F) and 1 м LiFSI DMC:FEC (5:5 by volume) (identified as LFD5F) were prepared. This preparation was conducted within an argon-purged glove box, ensuring that the levels of oxygen and moisture were kept below 1.0 ppm. The commercial electrolyte was 1 mol L^-1^ LiPF_6_ in ethylene carbonate (EC) and diethyl carbonate (DEC) (1:1 in volume) with 10 wt.% fluoroethylene carbonate (FEC) and 1 wt.% vinylene carbonate (VC) as additives (Purchased from Canrd Technology Co. Ltd).

**Calculation of coordination Number (CN)**

The average solvation number, which is the proportion of solvent molecules that coordinate with Li^+^ cations, can be ascertained based on the following relationship: ^[1]^

$\frac{A_{C-S}}{A_{C-S}+A_{F-S}}$ = N $\frac{C_{\mathrm{Li}}}{C_{S}}$

In this formula, $A_{C-S}$ and $A_{F-S}$ denote the Raman peak areas corresponding to the coordinated solvents and free solvents, respectively. $C_{\mathrm{Li}}$ and $C_{S}$ represent the concentrations of lithium salt and the solvents, respectively. The term N refers to the coordination number (CN) of the solvents. For the electrolytes containing two solvents, the CN value is the sum of the CN values of these two solvents. The CN of the FSI^-^ anion is determined by subtracting the CN of the solvent from 4.

**Electrochemical measurements**

The active material, acetylene black, and binders (styrene butadiene rubber (SBR) and sodium carboxymethyl cellulose (CMC) in a 1:1 mass ratio) were combined with deionized water in a 7:1.5:1.5 mass ratio to form a slurry. This slurry was thoroughly mixed using magnetic stirring. Once the mixture was well homogenized, it was applied to copper foil and then pre-dried in a drying oven at 60°C for 10 minutes. Subsequently, the foil was moved to a vacuum drying oven and dried at 60°C for an additional 8 hours. The dried electrode was then compressed and cut into discs measuring 12 mm in diameter for use in further experiments. Coin-type half cells (CR2032) were constructed within an argon-filled glove box (Lab2000, Etelux). Lithium metal served as the counter electrode, and a polypropylene (PP) separator was used to complete the cell assembly. The electrochemical properties of the batteries were evaluated within a voltage range of 0.01 to 3 V using the Neware CT-4000 battery tester from Shenzhen, China. The capacity is calculated based on phosphorus, and the 1C rate is defined as 2600 mAh g⁻¹. The areal capacity of the half cell is 0.92-1.1 mAh cm^-2^. In the case of a symmetrical Li||Li battery, both electrodes consist of lithium metal with a diameter of 14 mm, and they are operated with a capacity of 0.5 mAh cm^−2^ at 1 mA cm^−2^.

The galvanostatic intermittent titration technique (GITT) involved a 10-minute charge or discharge cycle at a 0.1C rate, succeeded by a 60-minute open circuit period. The Li^+^ diffusion coefficient can be determined from GITT curves using the following formula: ^[2]^

D_Li+_=$\frac{4}{\pi t}$($\frac{mV_{M}}{\mathrm{MA}}$)^2^($\frac{{\Delta E}_{s}}{{\Delta E}_{\tau}}$)^2^

The variables m, V_M_, M and A correspond to the mass of the active material, the molar volume, the molar mass, and the effective electrode area, respectively. Additionally, t refers to the duration of a single charging step, $\Delta E_{s}$ signifies the change in voltage during the steady state following the relaxation phase, and $\Delta E_{t}$ is the transient voltage variation observed during a single titration.

Additionally, cyclic voltammetry (CV) and electrochemical impedance spectroscopy (EIS) analyses were carried out with an electrochemical workstation, the VMP-300 bio-logic system, manufactured in Germany. Linear sweep voltammetry (LSV) measurements were conducted to characterize the reduction and oxidation process in the Li||Cu and Li||stainless-steel cells using different electrolyte at 0.1 mv s^-1^. According to CV results, the rapid kinetics can be further verified by examining the current. The description is as follows:

I(v)=k_1_ v+k_2_ v^1/2^

Herein, the current response can be separated into two components. The k_1_ν stands for a capacitive contribution, and the k_2_ν^1/2^ indicates a diffusion-controlled contribution. Moreover, their proportions can be ascertained by determining the values of k_1_ and k_2_ at each specific potential. ^[3]^ The ionic conductivity (σ) was calculated through the following equation: ^[4]^

σ = $\frac{d}{\mathrm{RS}}$

Where d (0.0025 cm) and S (2 cm^2^) belong to the thickness and area of electrolyte. R denotes bulk resistance attributed to stainless-steel/electrolyte/stainless-steel configuration. Activation energy ($E_{a}$) is calculated in accordance with the Arrhenius equation as follows:

$$\frac{1}{R_{\mathrm{ct}}}=Aexp\frac{-E_{a}}{\mathrm{RT}}$$

In the equation, $R_{\mathrm{ct}}$ represents the charge-transfer resistance; A is the Arrhenius constant; $E_{a}$ is denoted the desolvated activation energy; R is the gas constant; and T is the absolute temperature respectively. ^[5]^

During the initial discharge cycle, in-situ EIS was conducted using a VSP-300 Biologic multichannel electrochemical workstation. The process began with the battery being allowed to rest, ensuring electrochemical stability was achieved with a voltage change rate of less than 1 mV per hour prior to EIS testing. ^[6]^ Subsequently, EIS measurements were taken across a frequency spectrum from 10 mHz to 100 kHz. The battery was then discharged for a duration of 2 hours at a 0.1C rate. This sequence of measurements was repeated in a cyclic manner until the battery voltage dropped to 0.01 V.

The Li^+^ transference number ($t_{{Li}^{+}}$) was calculated using the Bruce-Vincent method in lithium symmetrical battery:

$t_{\mathrm{Li}^{+}}$= $\frac{I_{s}(\Delta V-I_{0}R_{0})}{I_{0}(\Delta V-I_{s}R_{s})}$

In this context, $\Delta V$ refers to the constant voltage (10 mV) that is applied during the polarization process. The terms $I_{0}$ and $I_{s}$ represent the initial and the steady-state currents, respectively. Similarly, $R_{0}$ and $R_{s}$ denote the initial and steady-state resistance values, respectively. ^[7]^

The variable $T_{\mathrm{sand}}$ denotes the time required for the Li^+^ ions to become depleted near the electrode surface, a point at which the transport of Li^+^ ions can no longer keep up with their consumption. In other words, $T_{\mathrm{sand}}$ is the period during which the Li^+^ concentration at the electrode surface approaches zero. A higher T_sand_ value indicates enhanced Li⁺ transport capability, signifying the electrolyte’s efficiency in maintaining Li⁺ supply to the electrode. ^[8]^ Testing is carried out using a lithium symmetrical battery. In line with the traditional diffusion model formulated by the Sand’s equation: ^[9]^

$T_{\mathrm{sand}}$ = π$D_{\mathrm{Li}^{+}}$($\frac{nC_{0}F}{2J(1-t_{\mathrm{Li}^{+}})}$)^2^

The Li^+^ diffusion coefficient is denoted by $D_{\mathrm{Li}^{+}}$ (in units of cm²/s), where n is the number of electrons involved per species reaction (with n=1 for Li^+^), $C_{0}$ is the concentration of Li^+^ ions (in mol/cm³), F is Faraday’s constant (96,485 C/mol), J is the applied current density, and $t_{\mathrm{Li}^{+}}$ is the apparent transference number for Li^+^ ions. The $D_{\mathrm{Li}^{+}}$ was measured based on the pulse polarization method, as follows: ^[7]^

$D_{\mathrm{Li}^{+}}$ = $\frac{l^{2}\tau m_{\ln}}{\pi^{2}}$

Here, l represents the distance between the two electrodes, which is equivalent to the combined thickness of five layers of separators (5 × 25 μm). The symbol $\tau$ denotes the tortuosity of the separator, which is taken as the reported value of 2.5. The parameter $m_{\ln}$ is determined by calculating the linear slope of the Napierian logarithm of the relaxation potential plotted against the relaxation time.

**Material characterization**

X-ray diffraction (XRD) patterns were obtained utilizing a Rigaku Smartlab 9000 W diffractometer, which features a copper (Cu) anode and operates at a wavelength (λ) of 1.54181 Å. The surface topography and microstructure were scrutinized through a Zeiss Sigma field emission scanning electron microscope (FE-SEM) and a JEM-F200 transmission electron microscope (TEM), with EDS elemental mapping performed on a JEOL JEM-2100 microscope at an acceleration voltage of 200 kV. Infrared (IR) spectroscopy was executed with a Nicolet 6700 Fourier-transform (FT) spectrometer by Thermo Scientific. For thermogravimetric analysis (TGA), an SDT-Q600 analyzer was employed, heating the samples from room temperature to 1000 °C in a nitrogen (N_2_) environment at a rate of 20 °C per minute and a gas flow of 100 ml/min. The specific surface area was evaluated by the Brunauer-Emmett-Teller (BET) technique using a surface characterization analyzer from Micromeritics. Raman spectroscopy measurements were made with a Micro Raman System (LABHRev-UV), using a Nd:YAG laser for excitation at 532 nm. X-ray photoelectron spectroscopy (XPS) was conducted with an Al Kα source (hν=1486 eV) on equipment from Thermo Fisher Scientific. UV-visible absorbance spectra were recorded on a Jasco V-770 UV-Vis Spectrophotometer. FIB-SIMS data were collected using a ZEISS FIB Crossbeam 540 with a Tofwerk TOF-SIMS. A compact design of time-of-flight (ToF) SIMS analyser (TOFWERK, Switzerland) added to a focused ion beam (FIB, ZEISS Crossbeam 540, Germany) was also used to resolve the segregation of Li on grain boundaries.

**Theoretical calculation**

All DFT simulations were conducted using the DMol3 module in Materials Studio 2023. In DFT calculations, the structure optimization, and calculation of energy, orbitals (The Highest Occupied Molecular Orbital: HOMO, the lowest unoccupied molecular orbitals: LUMO) as well as electrostatic potential calculation were performed at GGA/PBE functional level with TNP basis set. The energy, force and displacement convergence criteria are 10^-5^ Ha, 0.004 Ha/Å and 0.005 Å, respectively. The global orbital cutoff was set as 4.4 Å. The Brillouin zone was sampled by a 1×1×1 k-points grid. For LiP_x_ coordination complexes, the binding energy between the components was calculated as: E_b_=E_total_-E_A_-E_B_, where E_total_, E_A_, and E_B_ are the total energy of A-B configuration, A component, and B component, respectively, where A and B can refer to LiP_x_, solvent molecules or FSI^-^. In addition, their differential charge density is calculated by this formula Δρ_DCD_=ρ_AB_-ρ_A_-ρ_B_, where ρ_AB_, ρ_A_, and ρ_B_ are the electron density of A-B configuration, A component, and B component, respectively.

**1. Pristine material characterization section**

It has been reported in our previous work that the amorphous zinc phosphate plays multifunctional roles in weakening the agglomeration of BP nanomaterials, reducing the volume expansion, and improving the environmental stability of BP nanocomposite electrodes in humid air (Energy Storage Mater., 2023, 57, 400). Compared with zinc phosphate, lithium phosphate component has a better Li-ion conductivity, which is often used as coating material in Li ion/metal batteries (Adv. Mater. 2020, 32, 2000030). Thus, lithium phosphate was combined into the BP and carbon composites. Detailed structural and morphological characterizations are provided as follows.


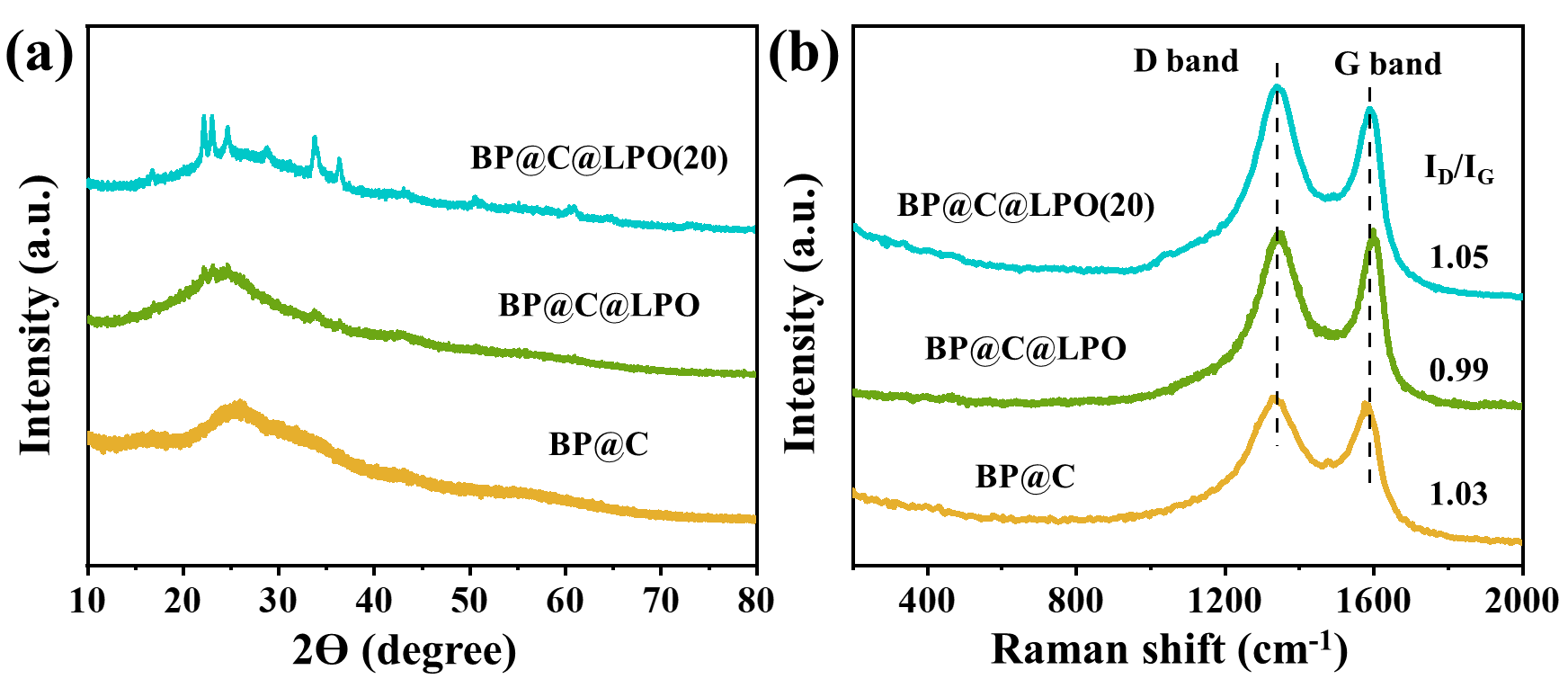


**Figure S1.** (a) XRD patterns and (b) Raman spectra for BP@C, BP@C@LPO and BP@C@LPO(20) samples. With the increasing amount of lithium phosphate, additional peaks appear in BP@C@LPO(20), which correspond to the crystalline lithium phosphate. Both BP@C and BP@C@LPO exhibit the amorphous features. Based on the Raman results, the I_D_/I_G_ is lowest in BP@C@LPO sample, indicating the lower number of defects. This suggests that the probability of side reactions occurring is relatively low.


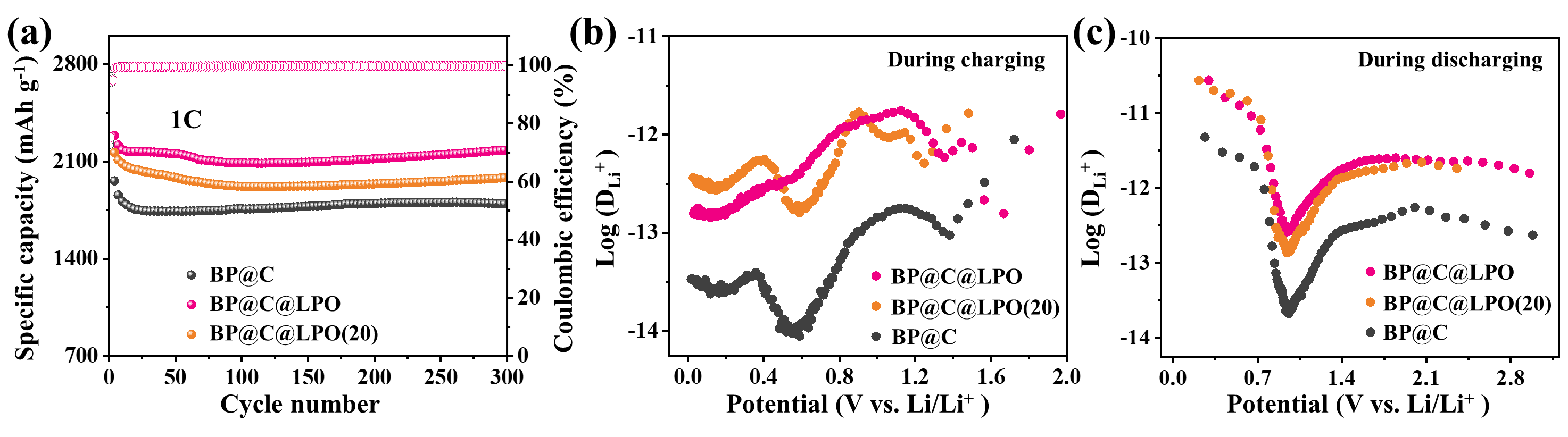


**Figure S2.** (a) Cycling performance at 1C and (b, c) The diffusion coefficient of lithium ions during the charging and discharging process of BP@C, BP@C@LPO and BP@C@LPO(20). The commercial electrolyte is used here. Compared with BP@C and BP@C@LPO(20), the BP@C@LPO sample delivery the highest capacity, the fastest ion diffusion rate. Thus, BP@C@LPO sample is the optimal sample in this work.


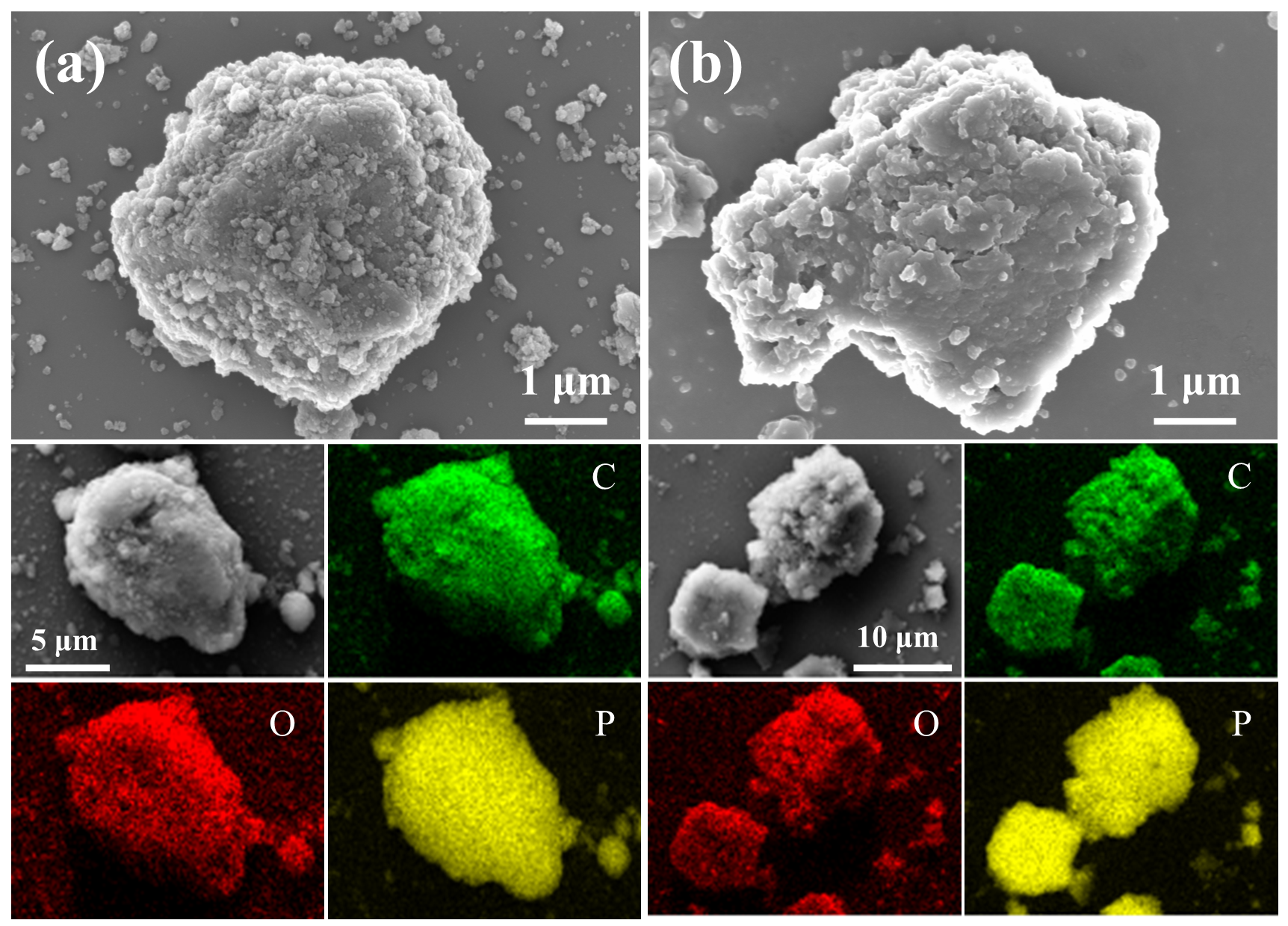


**Figure S3.** SEM images of (a) BP@C and (b) BP@C@LPO samples. It is obvious that these two composites are composed of secondary particles that form into spheres with irregular shapes and the elements are distributed uniformly.


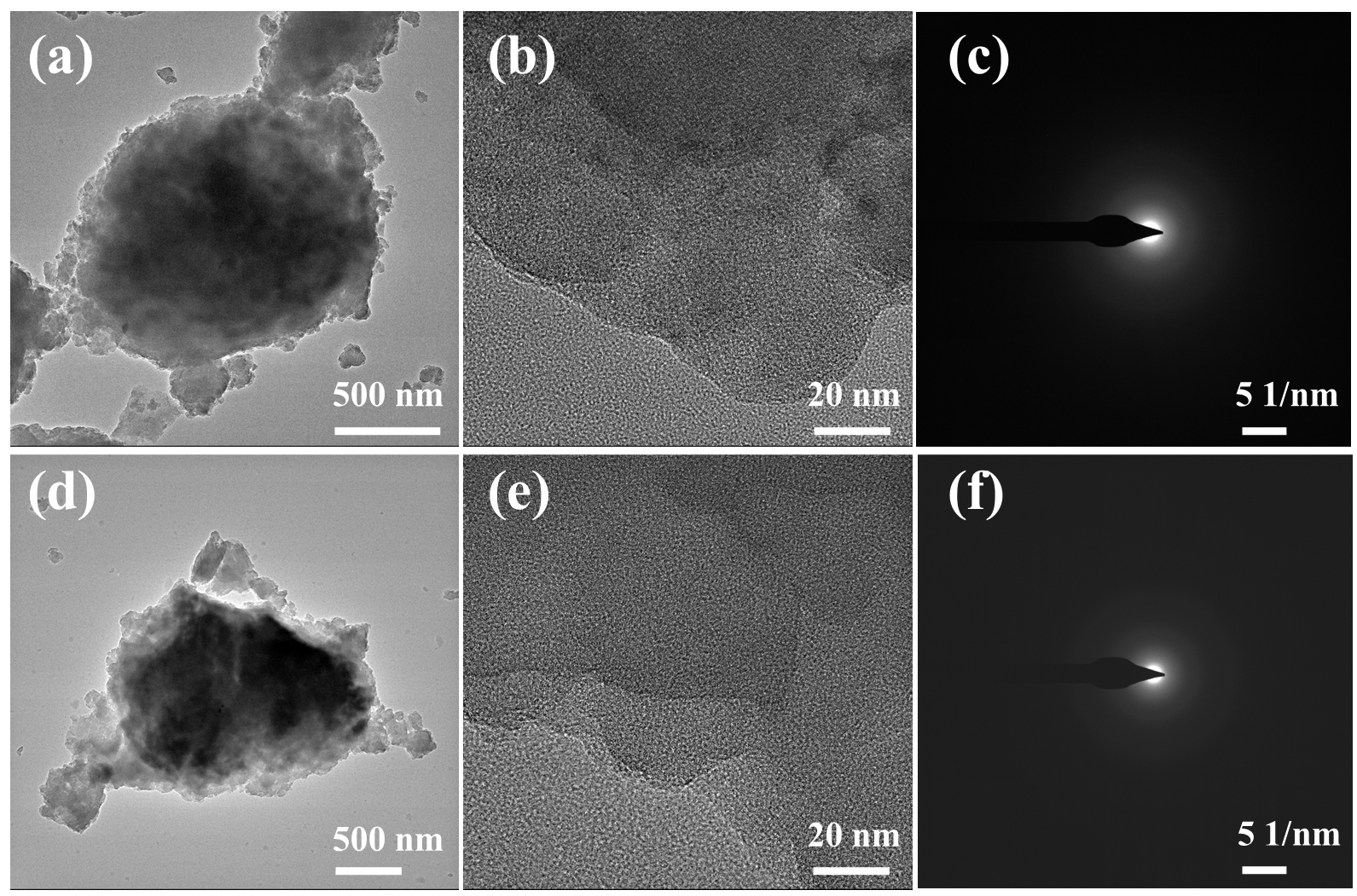


**Figure S4.** TEM images and SAED patterns of (a-c) BP@C and (d-f) BP@C@LPO samples. The absence of distinct lattice fringes or diffraction spots suggests that BP@C and BP@C@LPO exhibit amorphous characteristics.


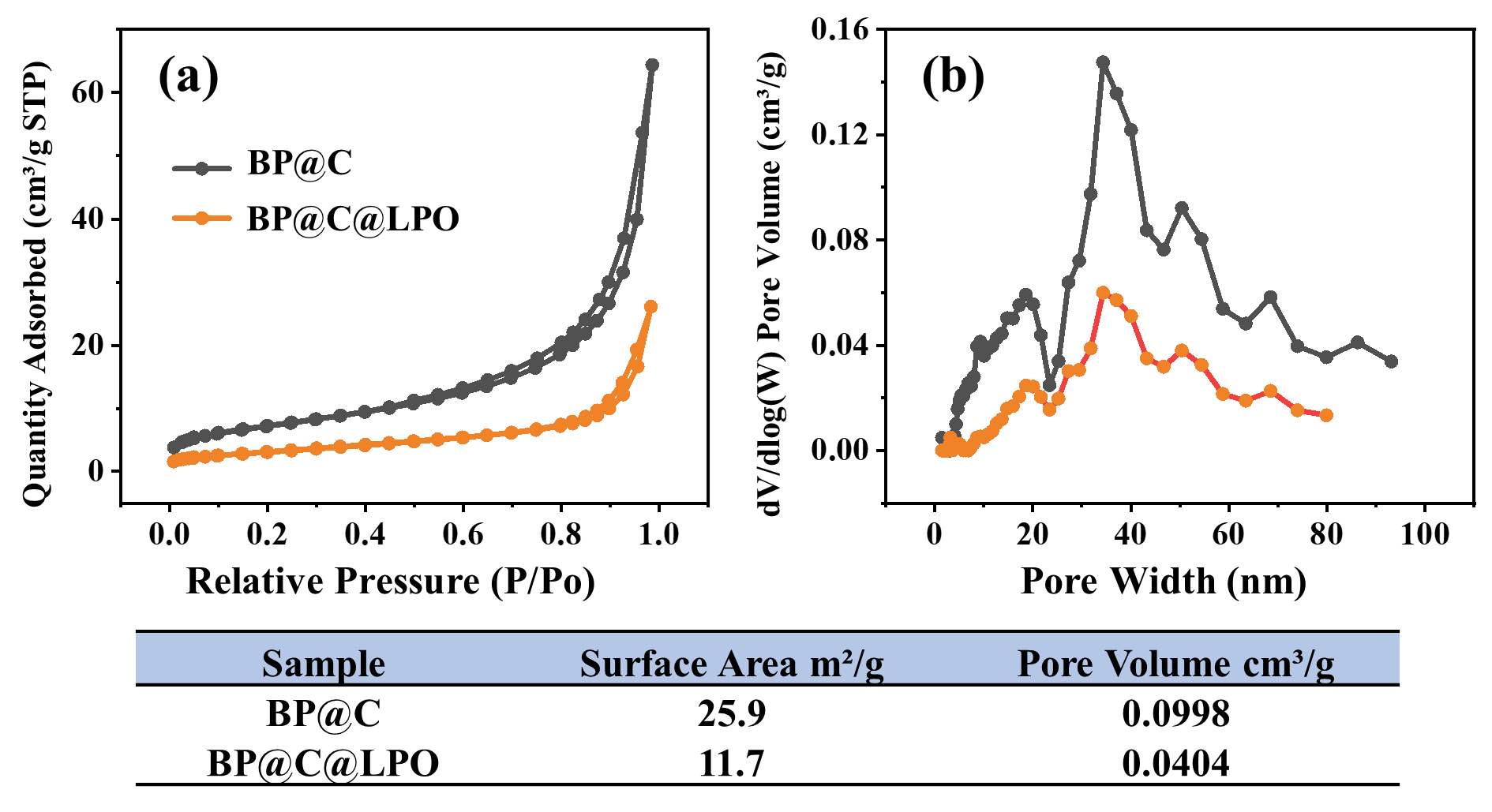


**Figure S5.** (a) Nitrogen adsorption-desorption isotherms and (b) pore size distribution of BP@C and BP@C@LPO respectively. The diminished surface area and pore volume of BP@C@LPO relative to BP@C are primarily due to the low surface area and pore volume characteristics of lithium phosphate.


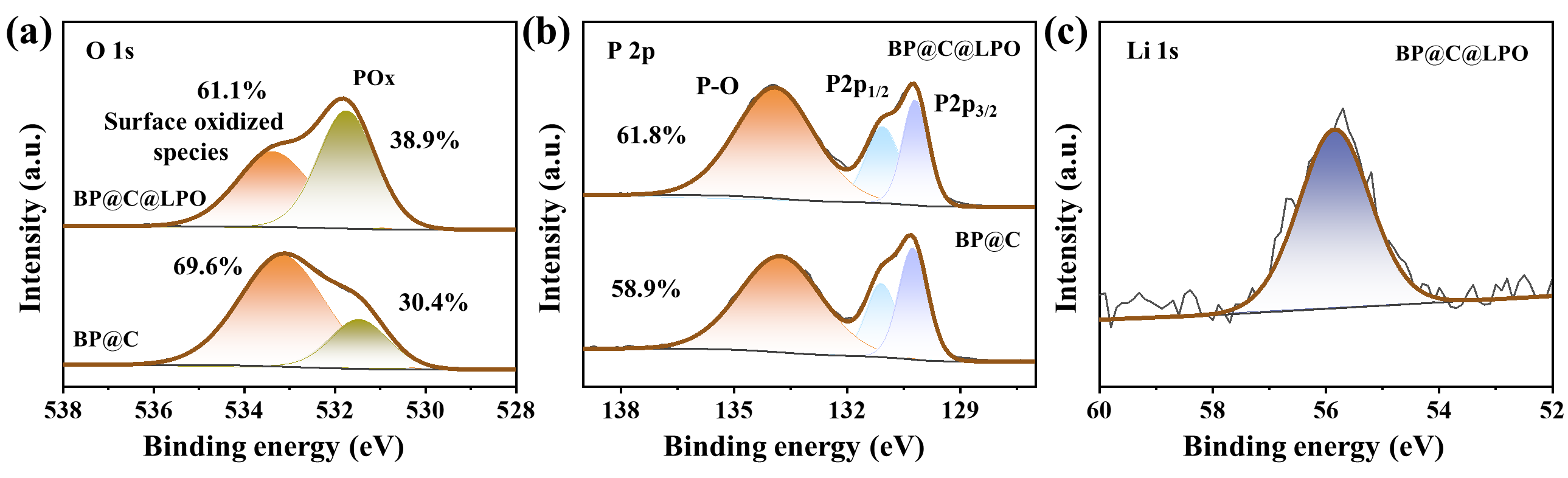


**Figure S6.** (a) O 1s, (b) P 2p spectra of BP@C and BP@C@LPO samples; (c) Li 1s spectrum of BP@C@LPO sample. The O 1s spectra reveal a reduced presence of surface-oxidized species in BP@C@LPO compared to BP@C, suggesting less surface contamination and the protective effect conferred by lithium phosphate. The presence of a slightly elevated P-O bond content in the P 2p spectra of BP@C@LPO can be attributed to the incorporation of lithium phosphate. The distinct peak observed in the Li 1s spectrum of BP@C@LPO confirms the presence of lithium elements within the material.


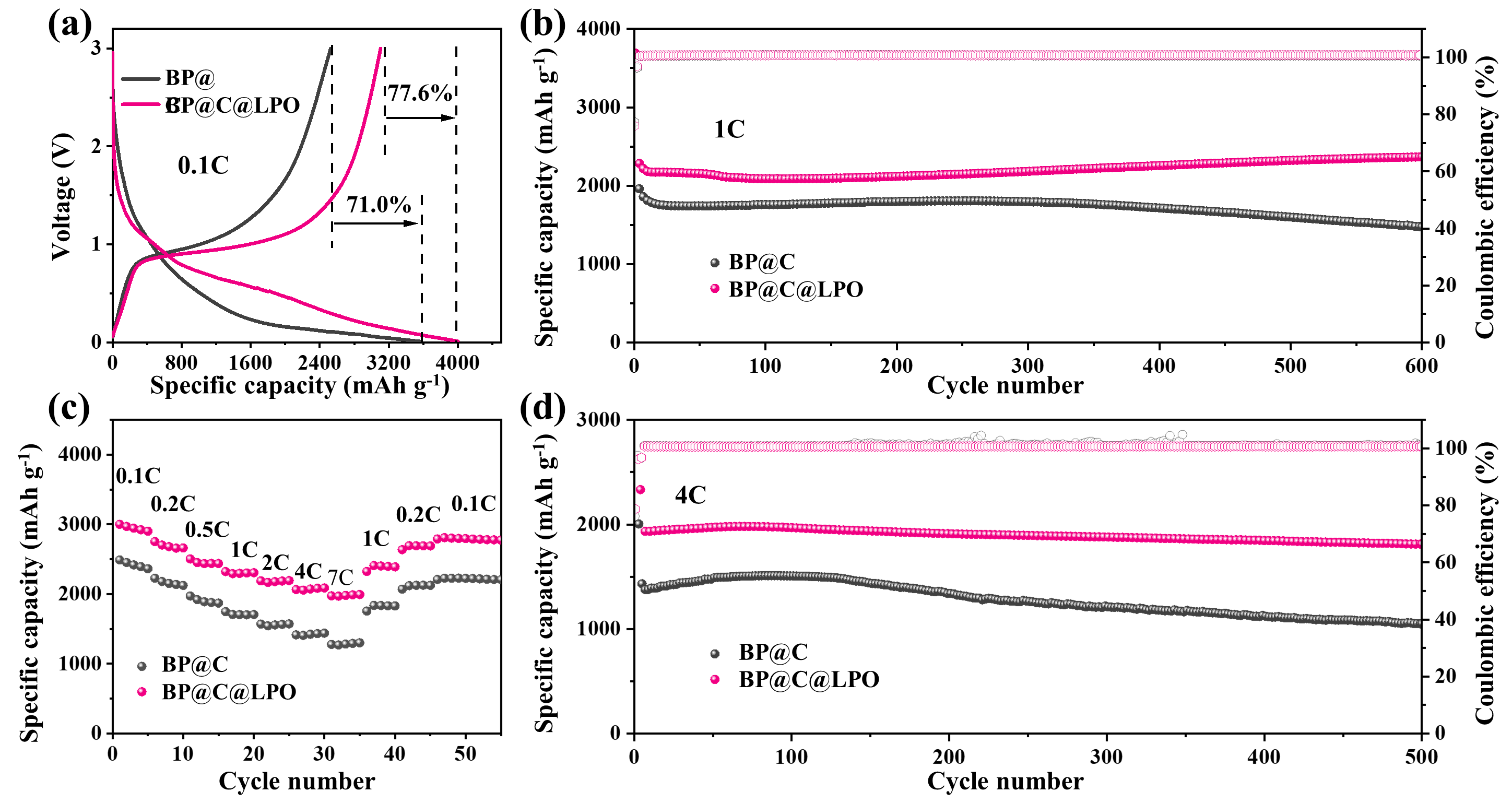


**Figure S7.** (a) Initial charge/discharge curves at 0.1C of BP@C and BP@C@LPO samples; (b) Cycling performance at 1C, (b) rate capability and (c) high-rate cycling performance at 4C of BP@C and BP@C@LPO samples. The commercial electrolyte is used here. BP@C@LPO demonstrates a superior initial coulombic efficiency (ICE) of 77.6%, surpassing that of BP@C, owing to its decreased defect density and reduced surface area. At a 1C rate, BP@C@LPO not only delivers a higher specific capacity but also exhibits superior capacity retention, maintaining nearly 100% after 600 cycles, in contrast to BP@C, which retains only 70.2%. BP@C@LPO demonstrates the better rate performance. At a 4C charging rate, the BP@C@LPO electrode achieves a delivery capacity of 1934 mAh g^-1^, accompanied by an impressive capacity retention rate of 97%. In comparison, the BP@C electrode provides a lower delivery capacity of 1378.1 mAh g^-1^, with a capacity retention rate of 76.3%. Following the comparative analysis, the phosphorus-based anode material BP@C@LPO has been selected for subsequent electrolyte optimization due to its superior performance.

**2. Electrolyte and cycled anode characterization section**


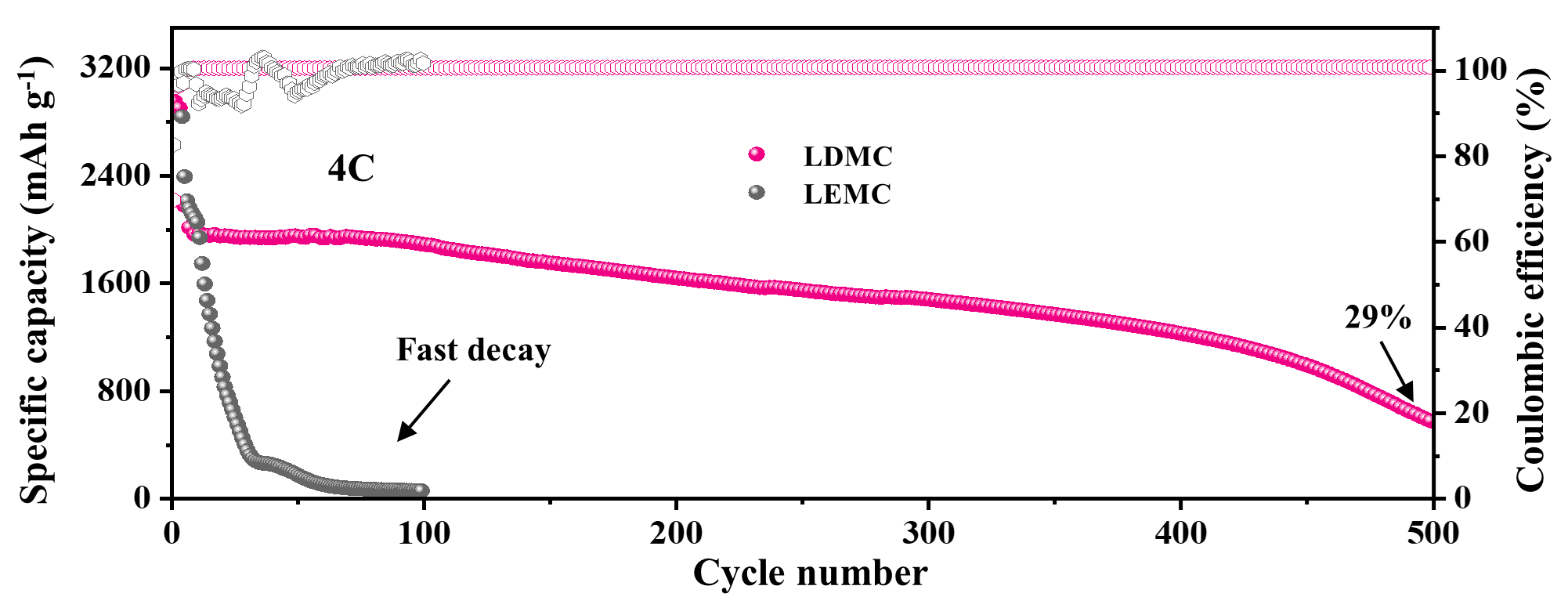


**Figure S8.** High-rate cycling performance of BP@C@LPO at 4C in LDMC and LEMC electrolytes.


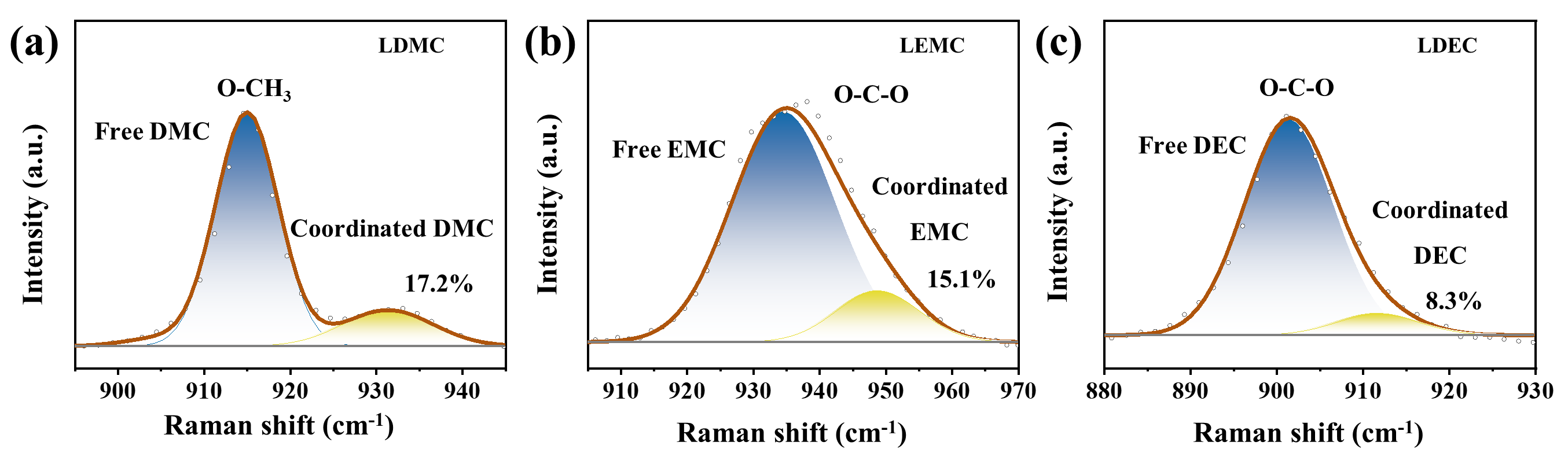


**Figure S9.** Fitting results of Raman spectra for (a) LDMC, (b) LEMC and (c) LDEC.


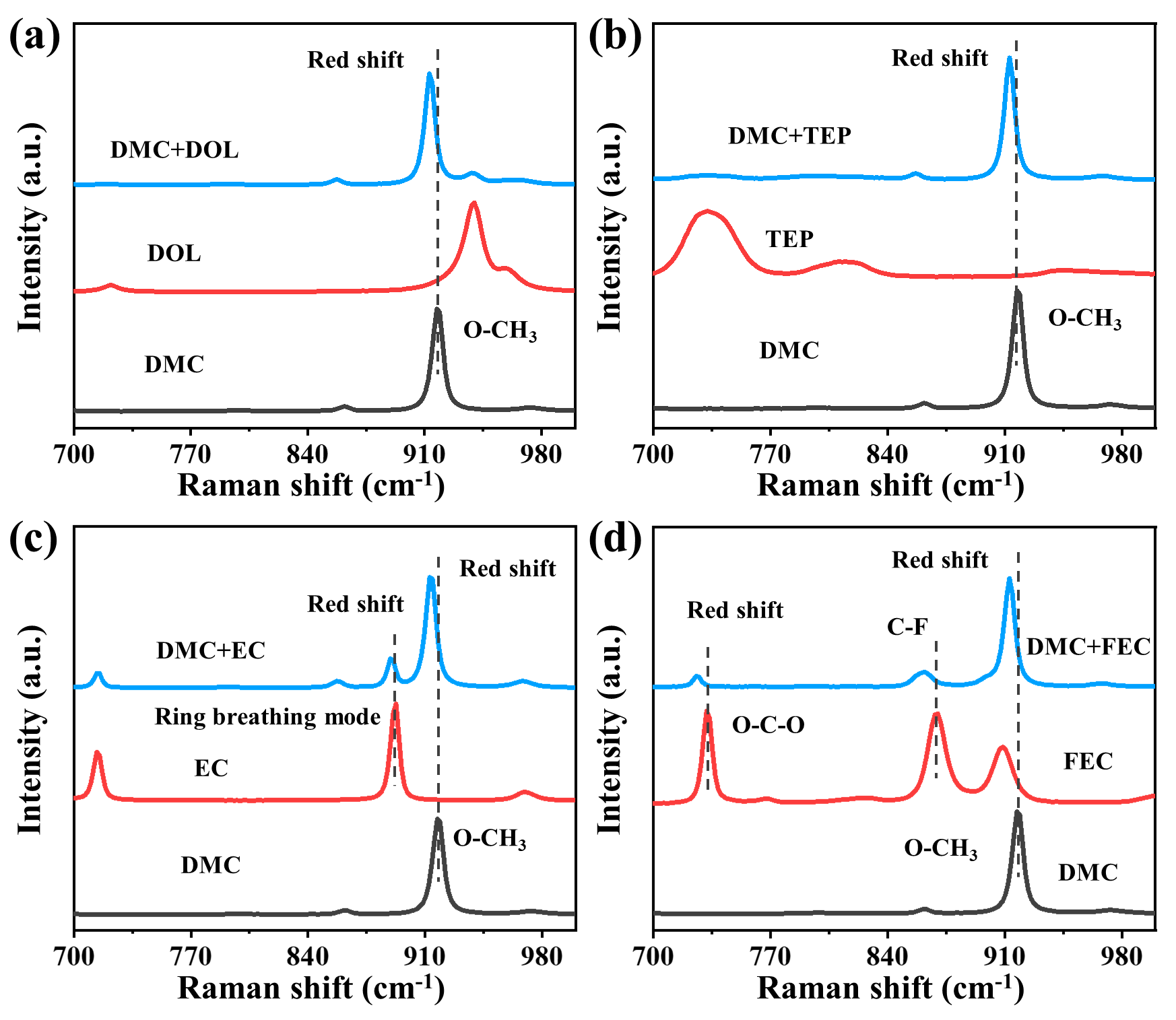


**Figure S10.** Raman spectra of (a) DMC single solvent, DOL single solvent and DMC, DOL mixed solvent, (b) DMC single solvent, TEP single solvent and DMC, TEP mixed solvent, (c) DMC single solvent, EC single solvent and DMC, EC mixed solvent, (d) DMC single solvent, FEC single solvent, DMC, FEC mixed solvent.


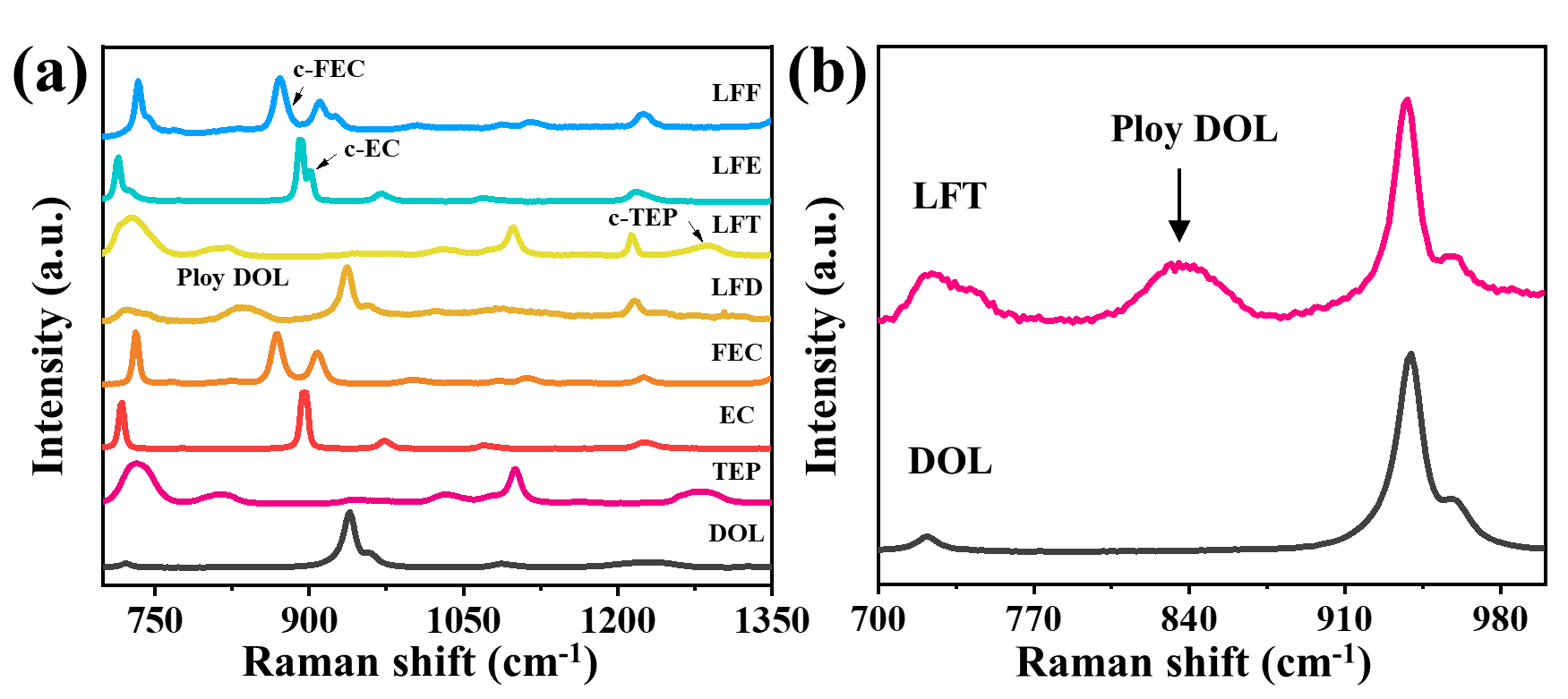


**Figure S11.** (a) Raman spectra of DOL, TEP, EC, FEC, LFD, LFT, LFE and LFF; (b) Raman spectra of DOL and LFT. The “c” in c-TEP, c-EC, c-FEC means the corresponding coordinated molecules. Raman spectra revealed that DOL and LiFSI underwent polymerization, as evidenced by characteristic spectral features. ^[10]^ In contrast, distinct peaks corresponding to coordinated TEP, EC, and FEC were observed, confirming their solvation behavior.


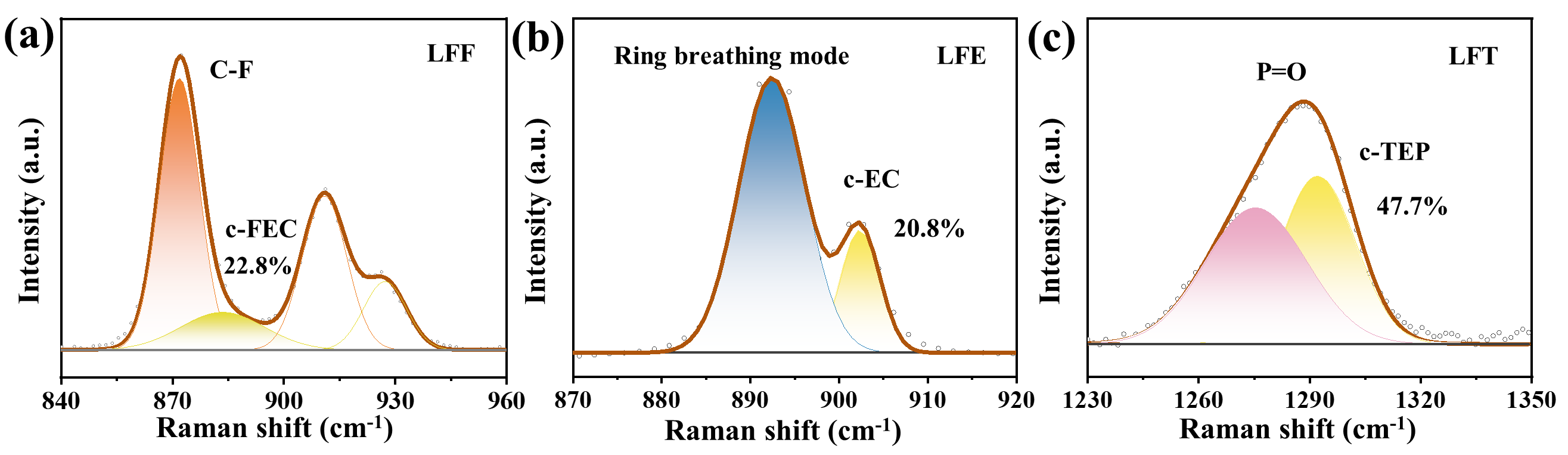


**Figure S12.** (a-c) Fitting Raman results for LFF, LFE and LFT. The “c” in c-FEC, c-EC, c-TEP means the corresponding coordinated molecules. Raman spectral fitting of LFF, LFE, and LFT revealed coordination proportions of 22.8% for FEC, 20.8% for EC, and 47.7% for TEP, with calculated coordination numbers of 3.1, 3.12, and 2.81, respectively.


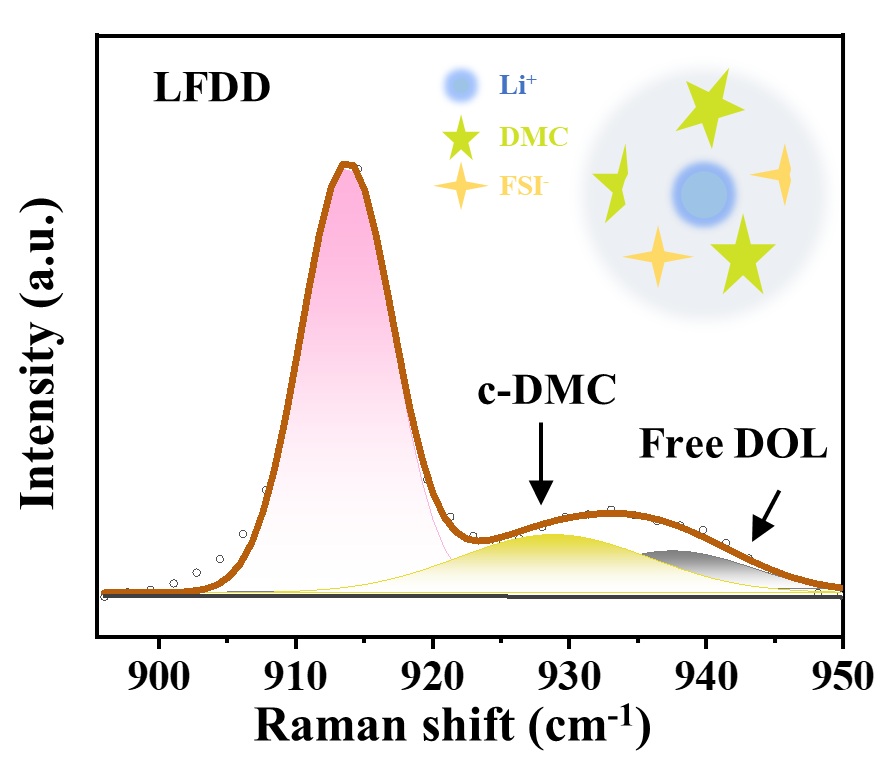


**Figure S13.** Fitting Raman results for LFDD. The “c” in c-DMC means the corresponding coordinated molecules.


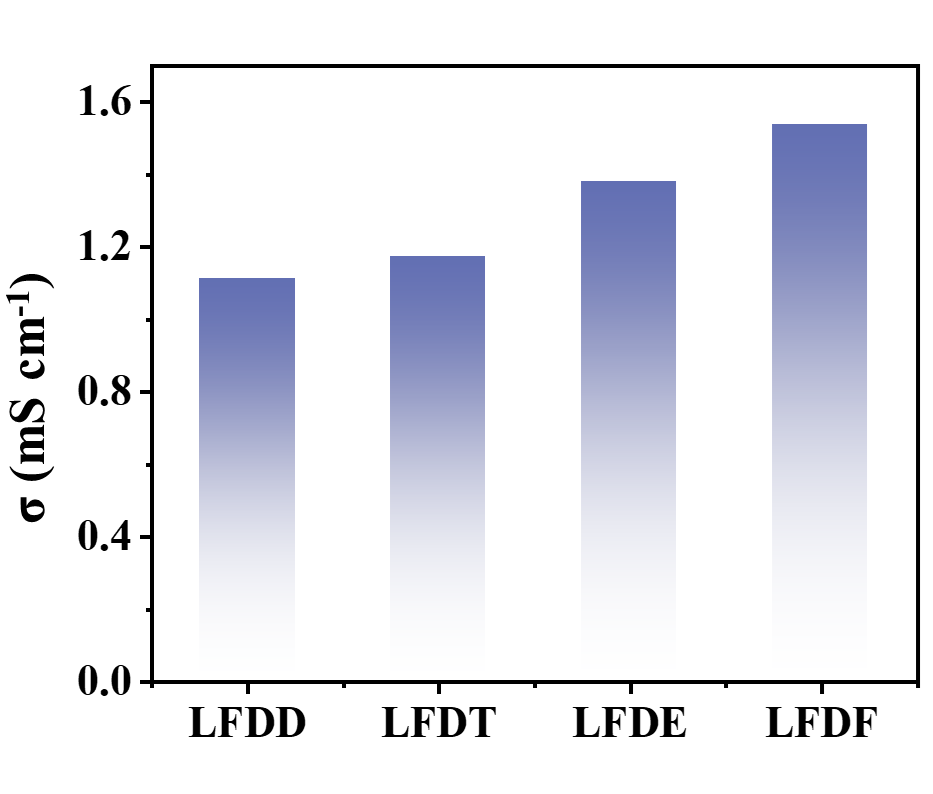


**Figure S14.** Ion conductivity of LFDD, LFDT, LFDE and LFDF.


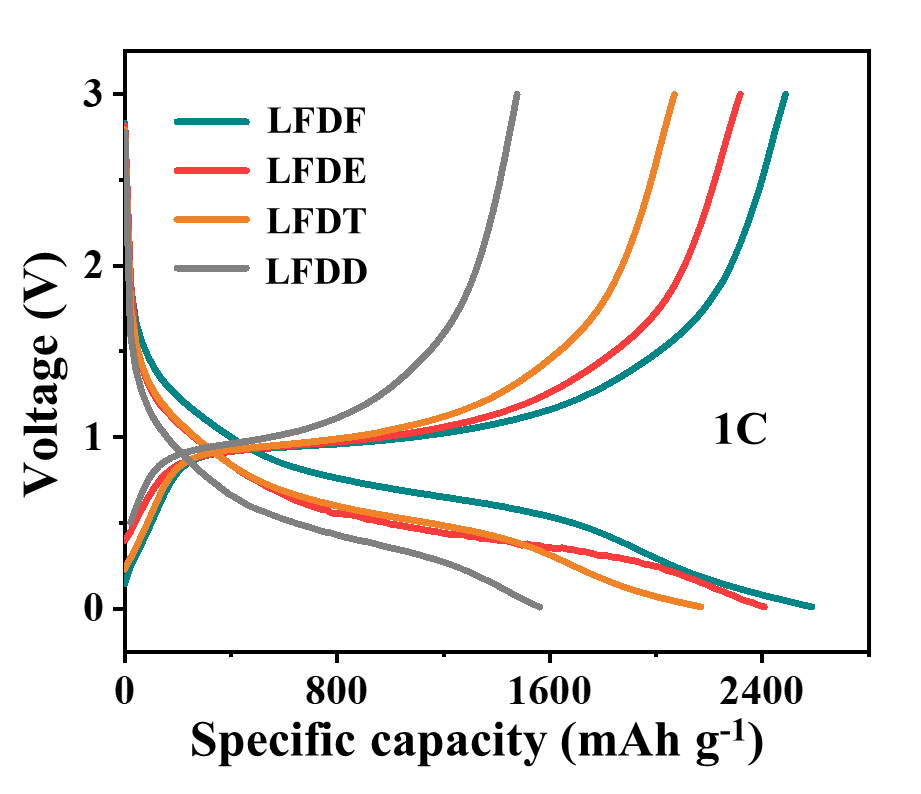


**Figure S15**. Charge/discharge curves of BP@C@LPO electrode in LFDD, LFDT, LFDE and LFDF.


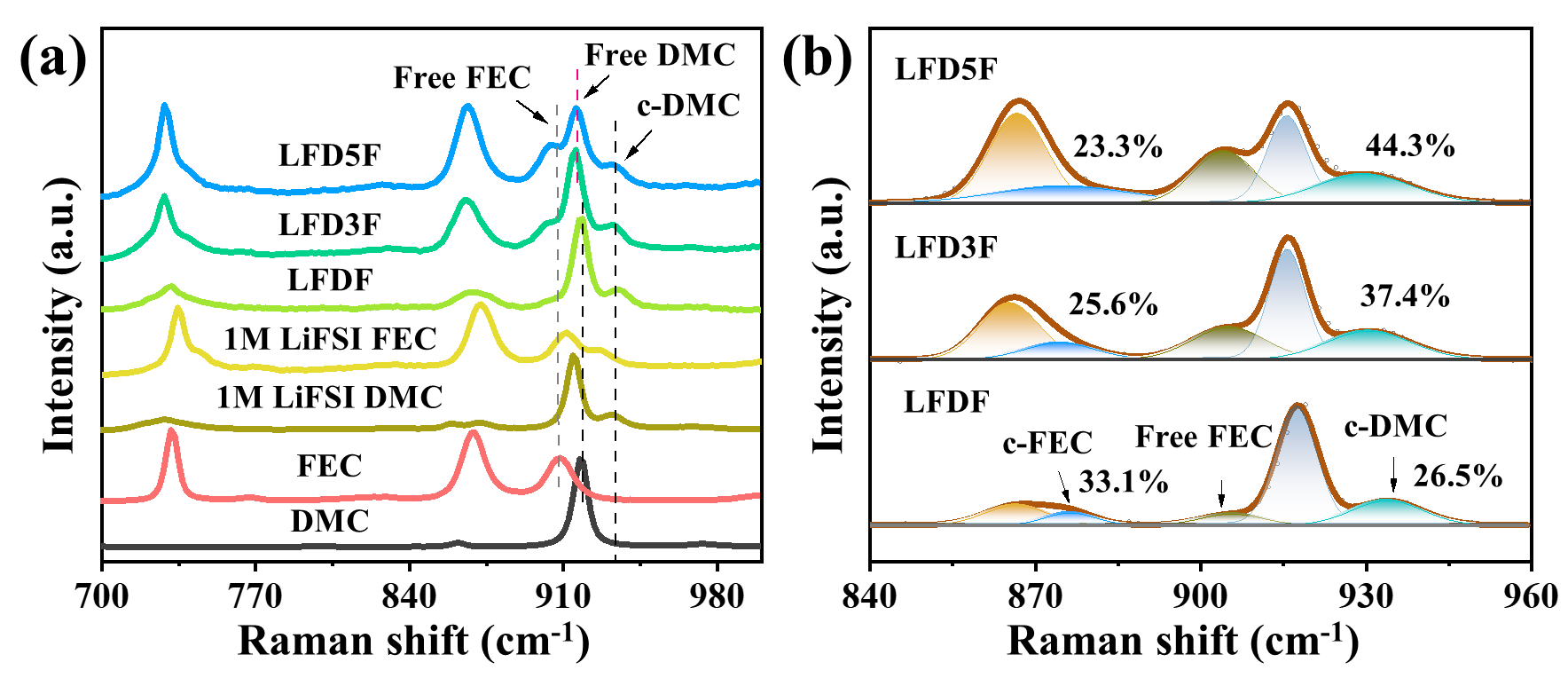


**Figure S16.** (a) Raman spectra of DMC, FEC, 1M LiFSI DMC, 1 м LiFSI FEC, LFDF, LFD3F and LFD5F; (b) Fitting Raman results of LFDF, LFD3F (1 м LiFSI DMC:FEC, 7:3 by volume) and LFD5F (1 м LiFSI DMC:FEC, 5:5 by volume). As the FEC content increased to 10%, 30%, and 50%, the coordination number of solvent molecules also increased, reaching values of 3.28, 4.16, and 4.23 in LFDF, LFD3F, and LFD5F, respectively (**Table S2**).


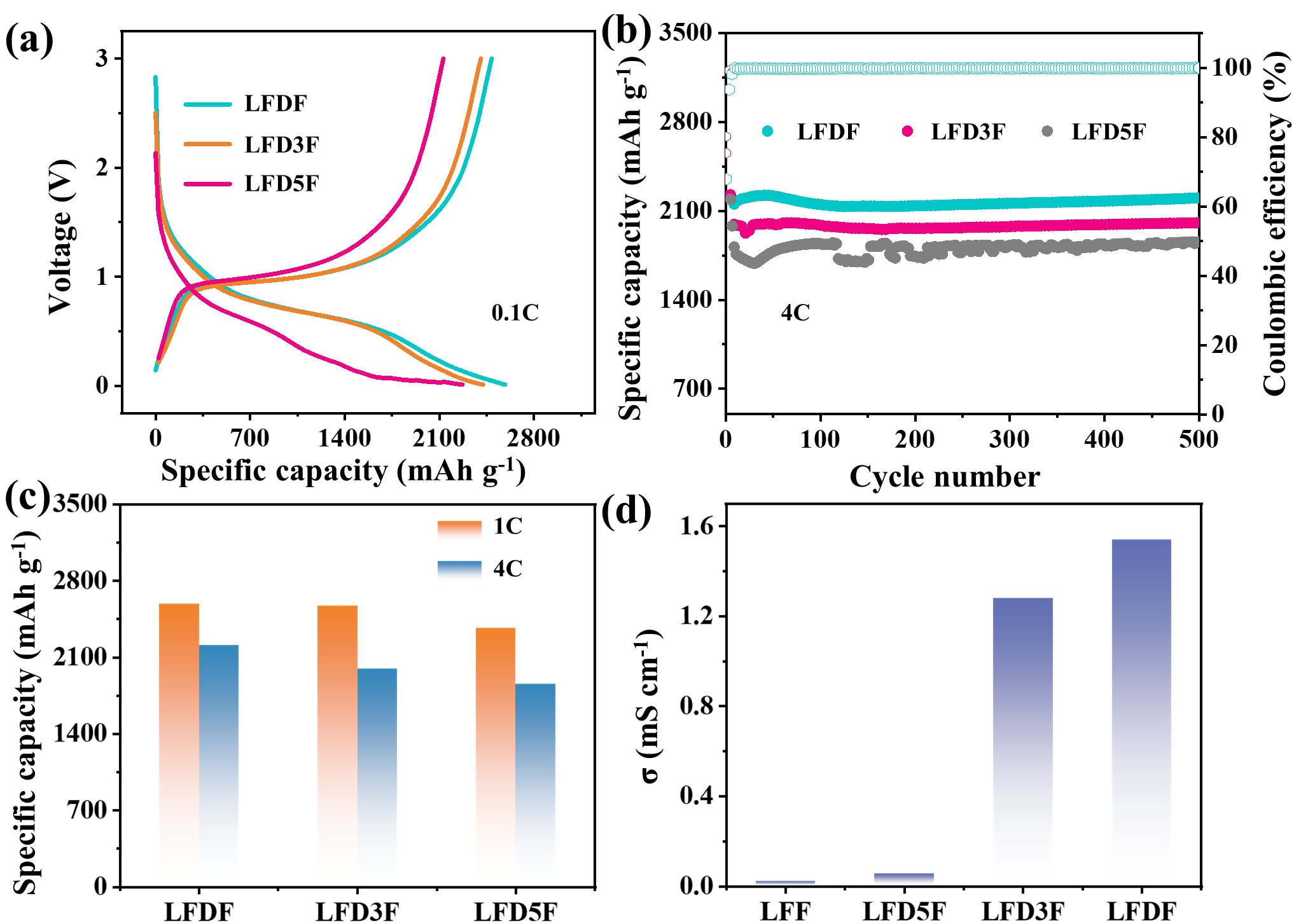


**Figure S17.** (a) Charge/discharge curves, long cycling performance conducted at 4C and specific capacity of BP@C@LPO electrode at 1C and 4C in LFDF, LFD3F and LFD5F; (d) Ion conductivity of LFF, LFDF, LFD3F and LFD5F. Electrochemical evaluation revealed that the BP@C@LPO electrode achieved the highest capacities at 0.1C, 1C, and 4C when paired with the LFDF electrolyte, which also exhibited the highest ionic conductivity. These findings suggest that an optimal coordination number is critical for superior electrochemical performance. This is attributed to the ability of an optimally coordinated solvation structure to maintain weak solvation stability while simultaneously ensuring high ionic conductivity and rapid Li⁺ desolvation kinetics.


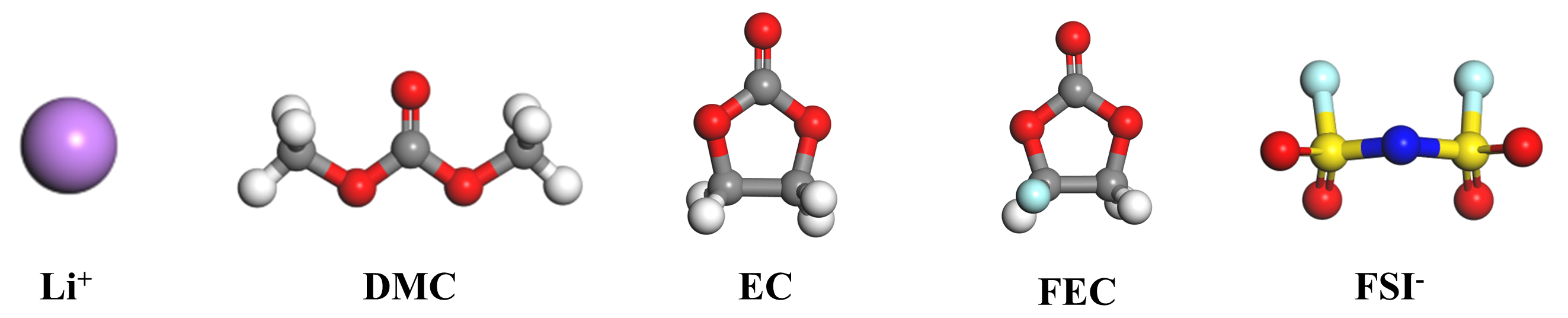


**Figure S18.** Structural optimization of solvent and salt molecules.


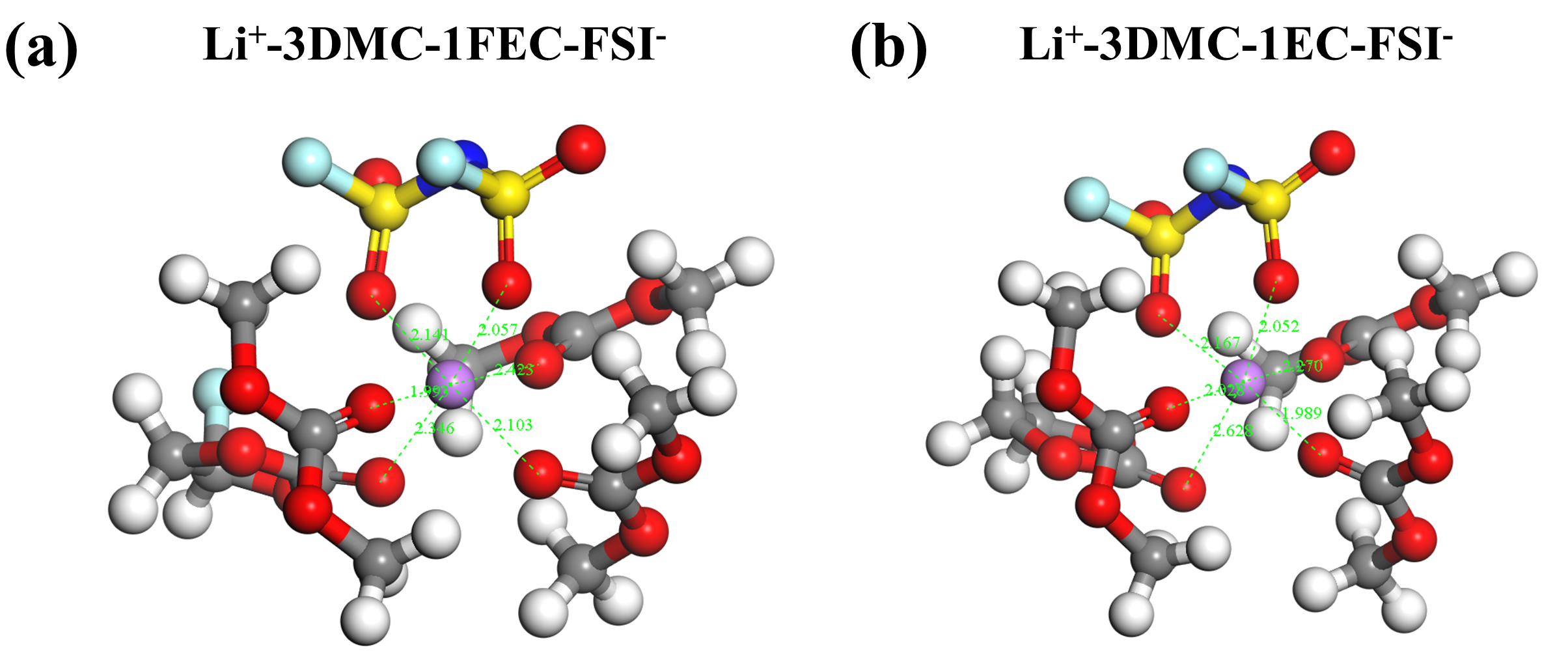


**Figure S19.** The distances between Li⁺ and the coordinated components.


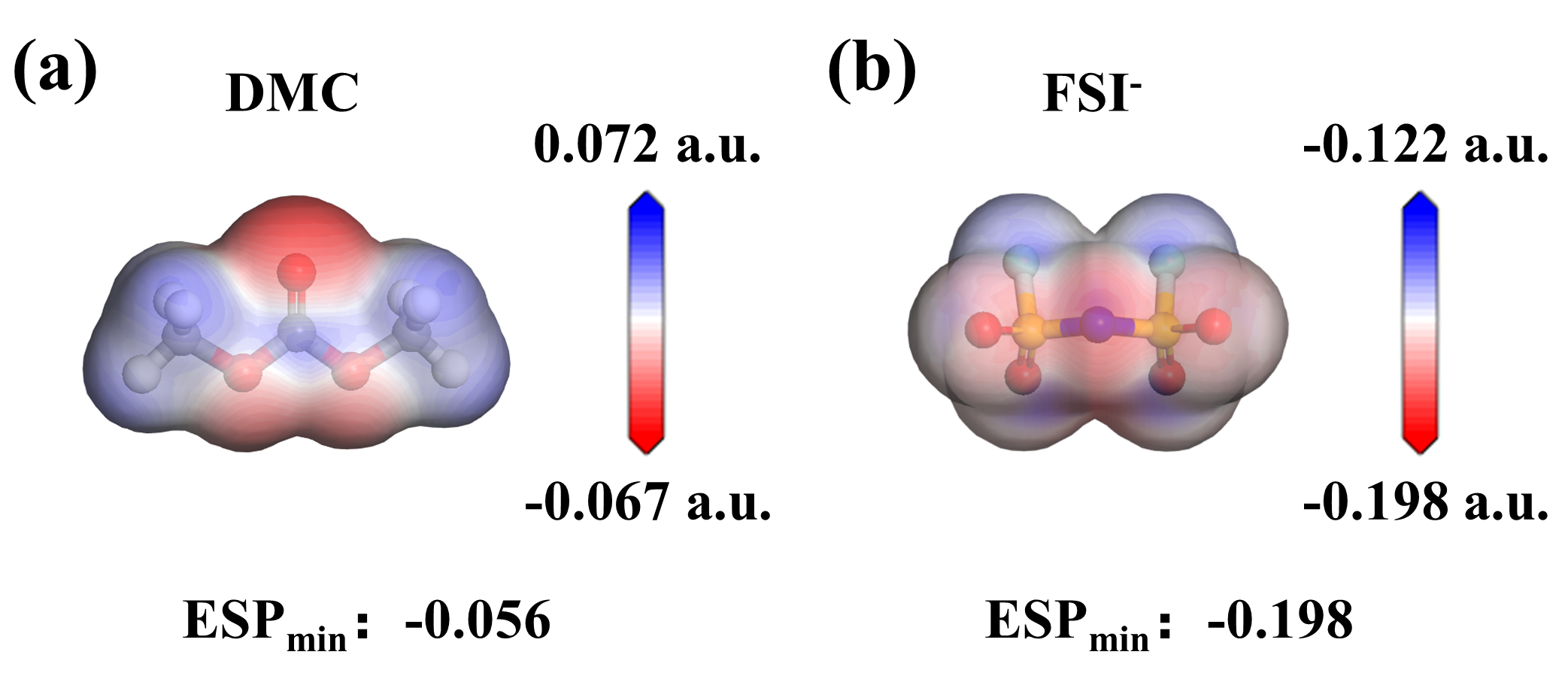


**Figure S20.** Electrostatic potential mapping of DMC and FSI^-^.


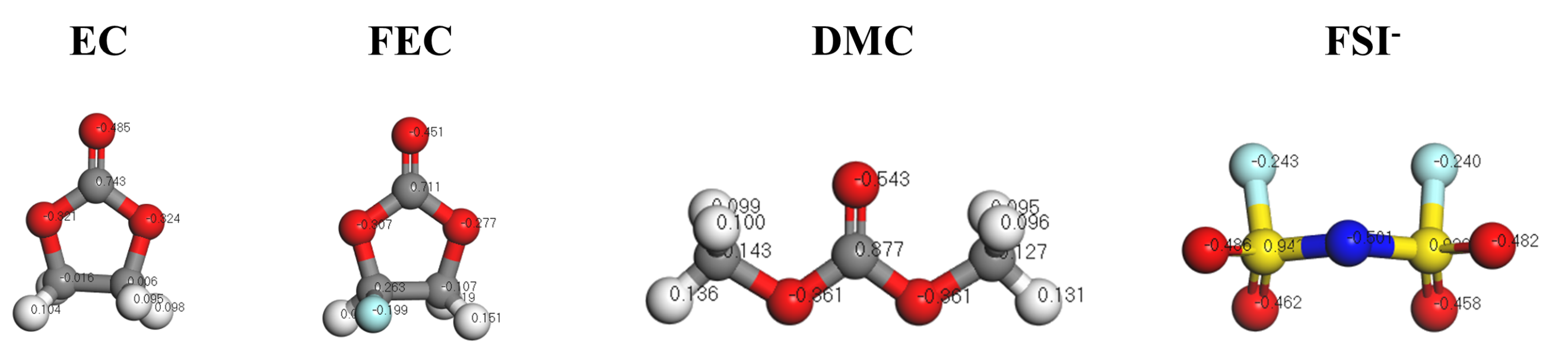


**Figure S21.** Charge distribution of EC, FEC, DMC and FSI^-^.


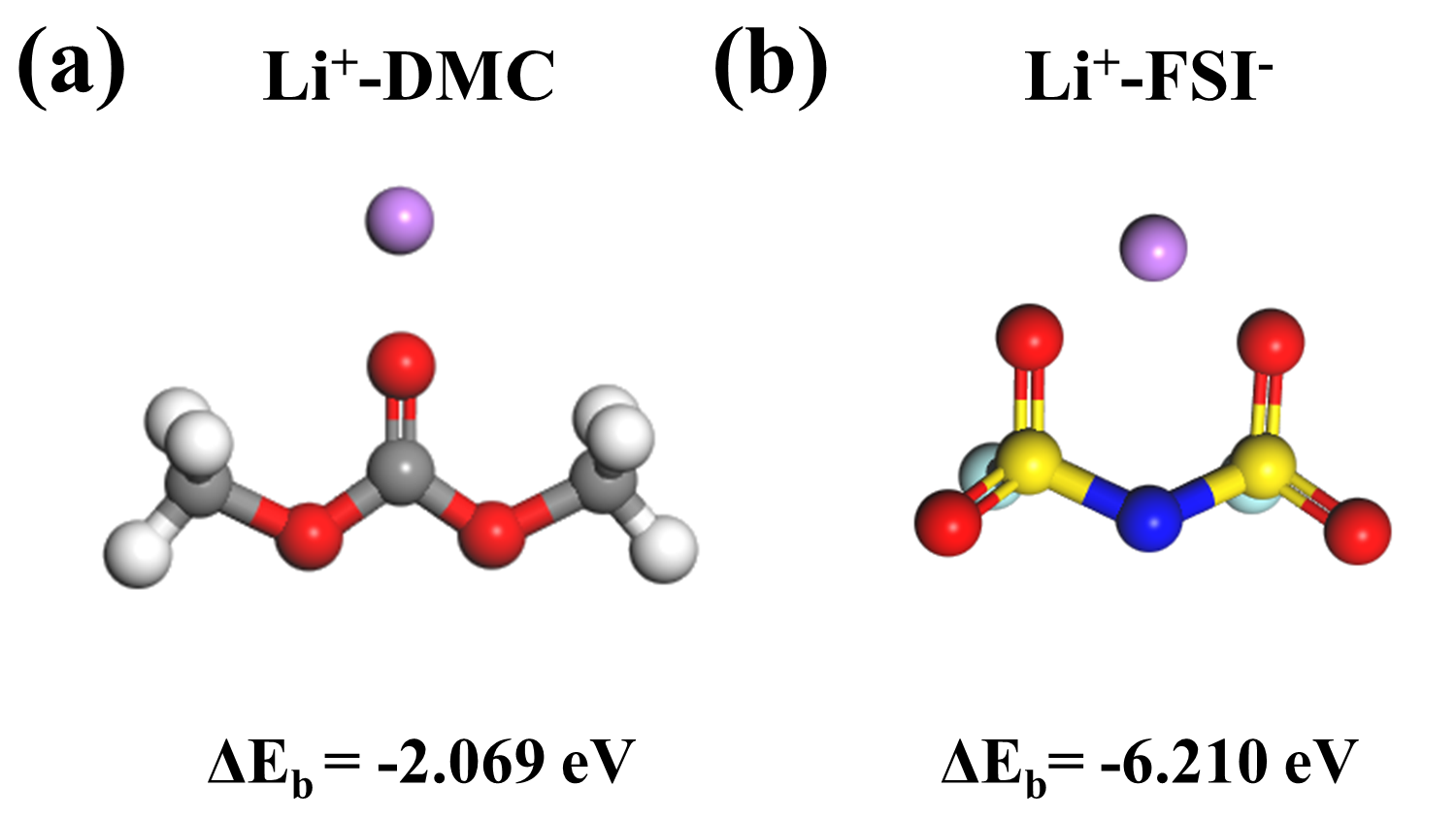


**Figure S22.** Binding energy of Li^+^ and anions/solvents.


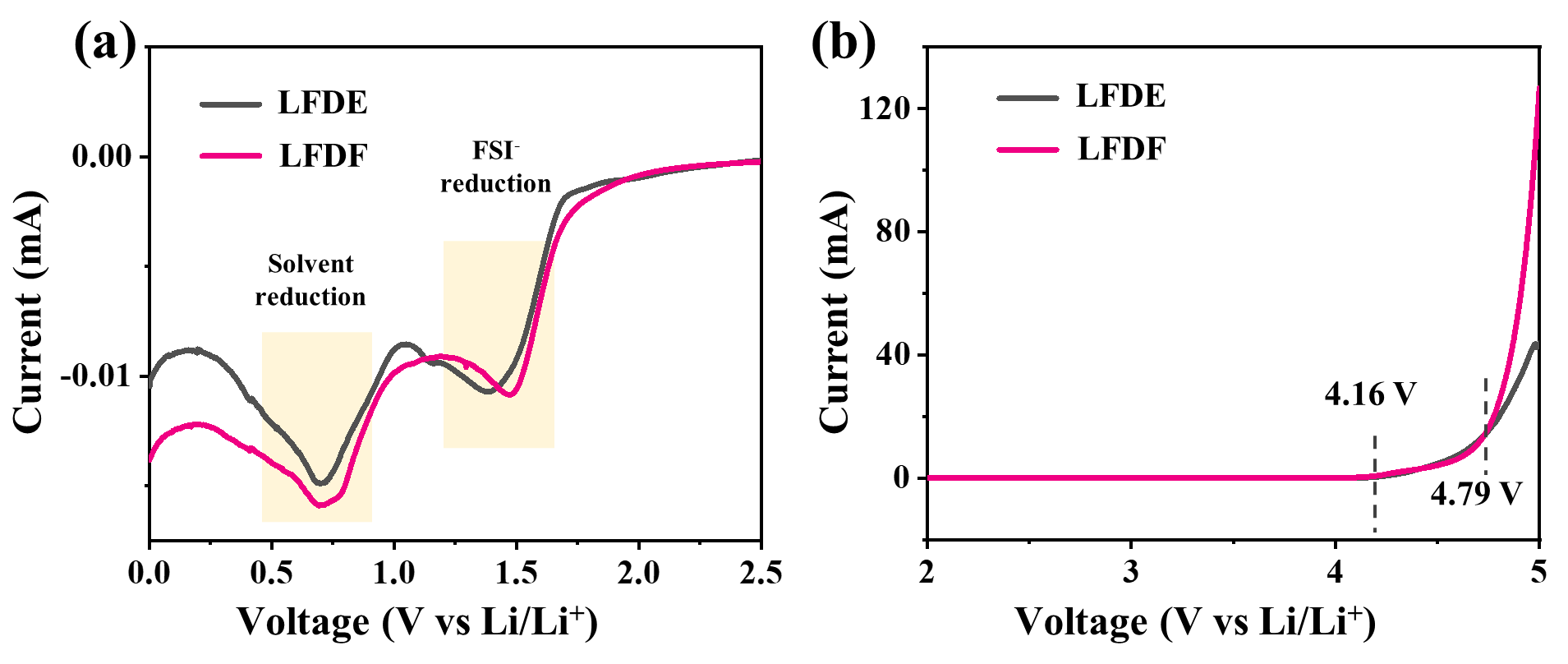


**Figure S23.** The linear sweep voltammograms of the LFDE and LFDF electrolytes using (a) Li||Cu and (b) Li||stainless-steel cells at 0.1 mv s^-1^. The reduction peak at ~1.5 V corresponds to FSI⁻ reduction, while the peak at ~0.75 V is attributed to solvent reduction. ^[11]^ The more pronounced FSI⁻ reduction peak in LFDF compared to LFDE suggests that fluorination enhances the reduction of electrolyte species, consistent with the calculated LUMO energy levels. Moreover, both electrolytes exhibit comparable oxidation stability.


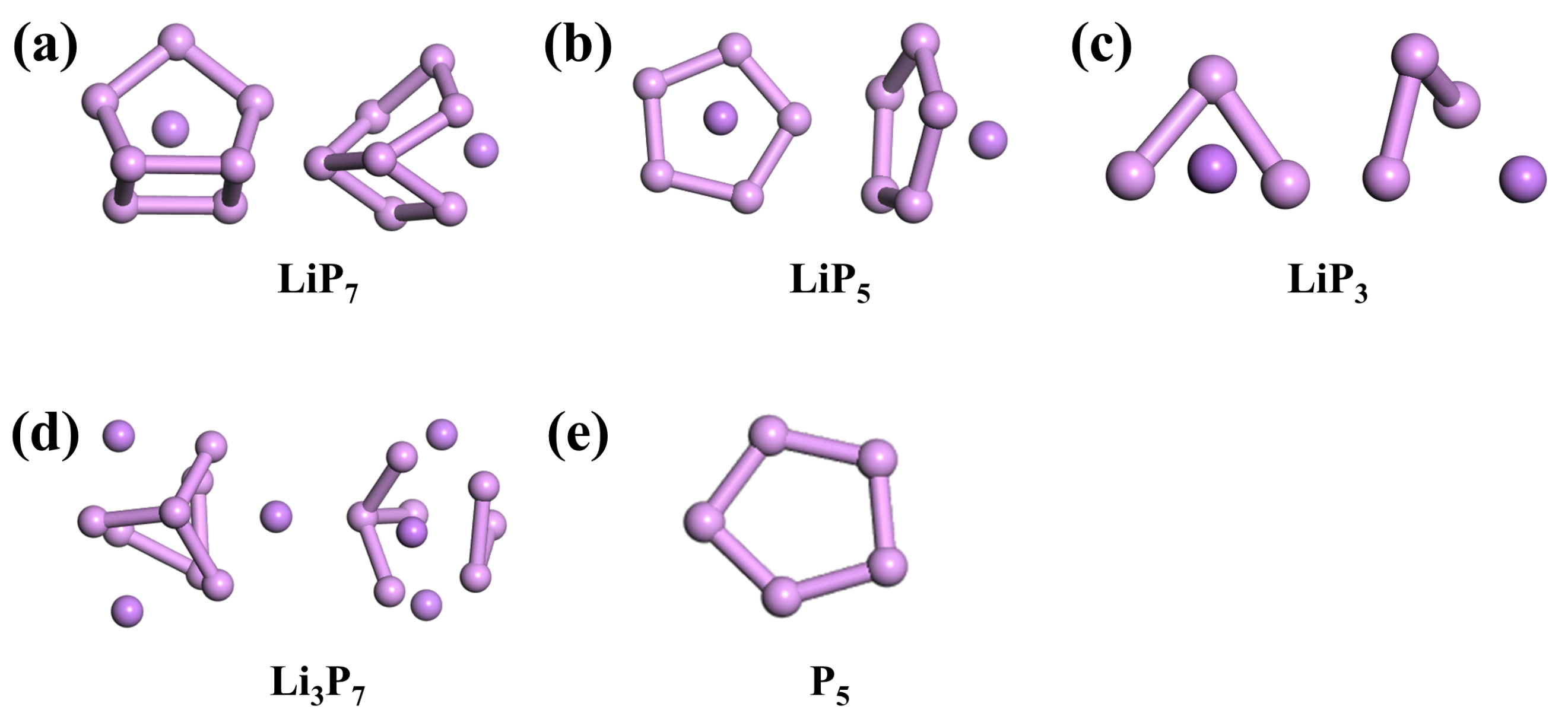


**Figure S24.** Structural optimization of lithium polyphosphide molecules.


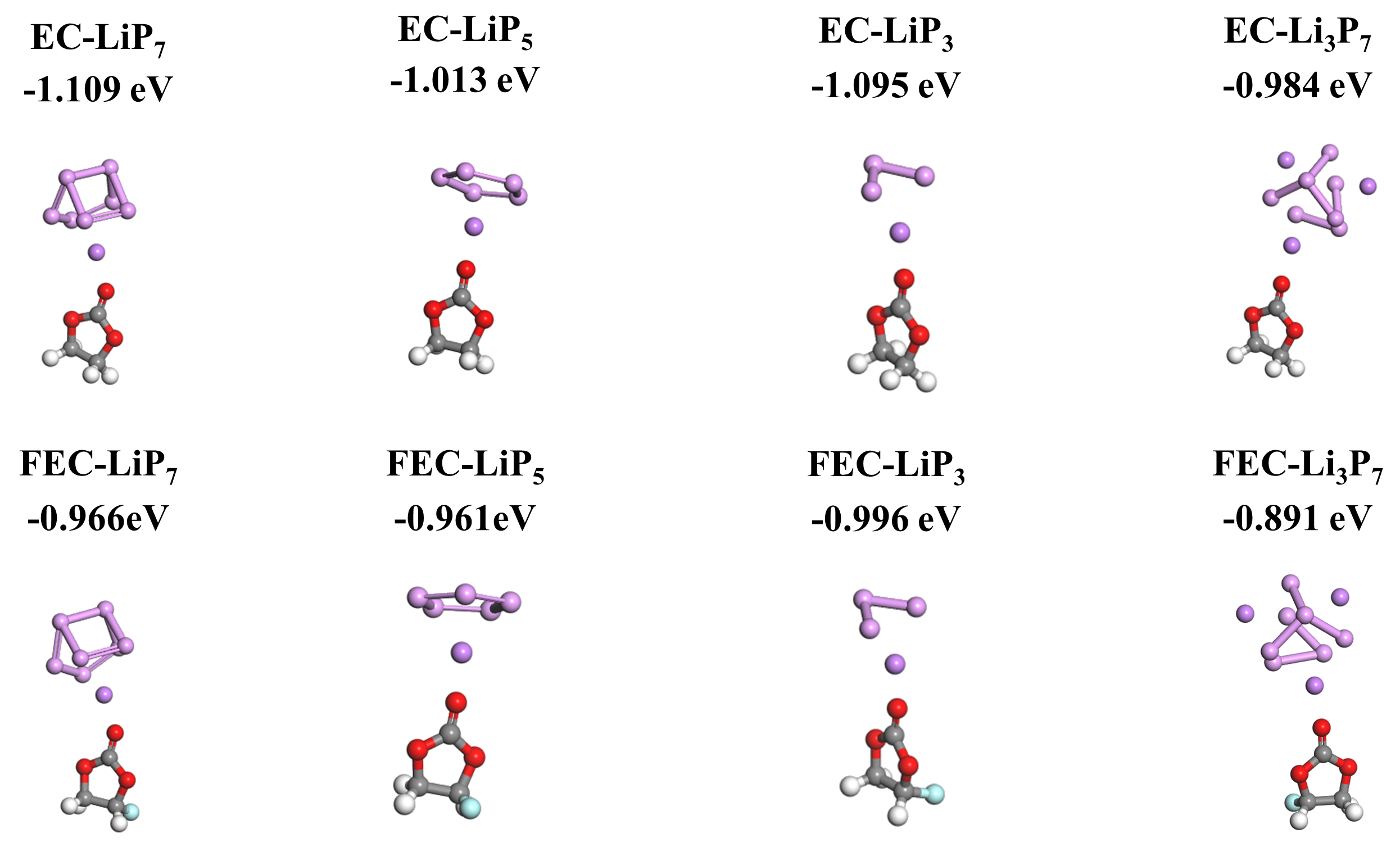


**Figure S25.** Adsorption energy of EC and FEC with LiP_7_/LiP_5_/LiP_3_/Li_3_P_7_.


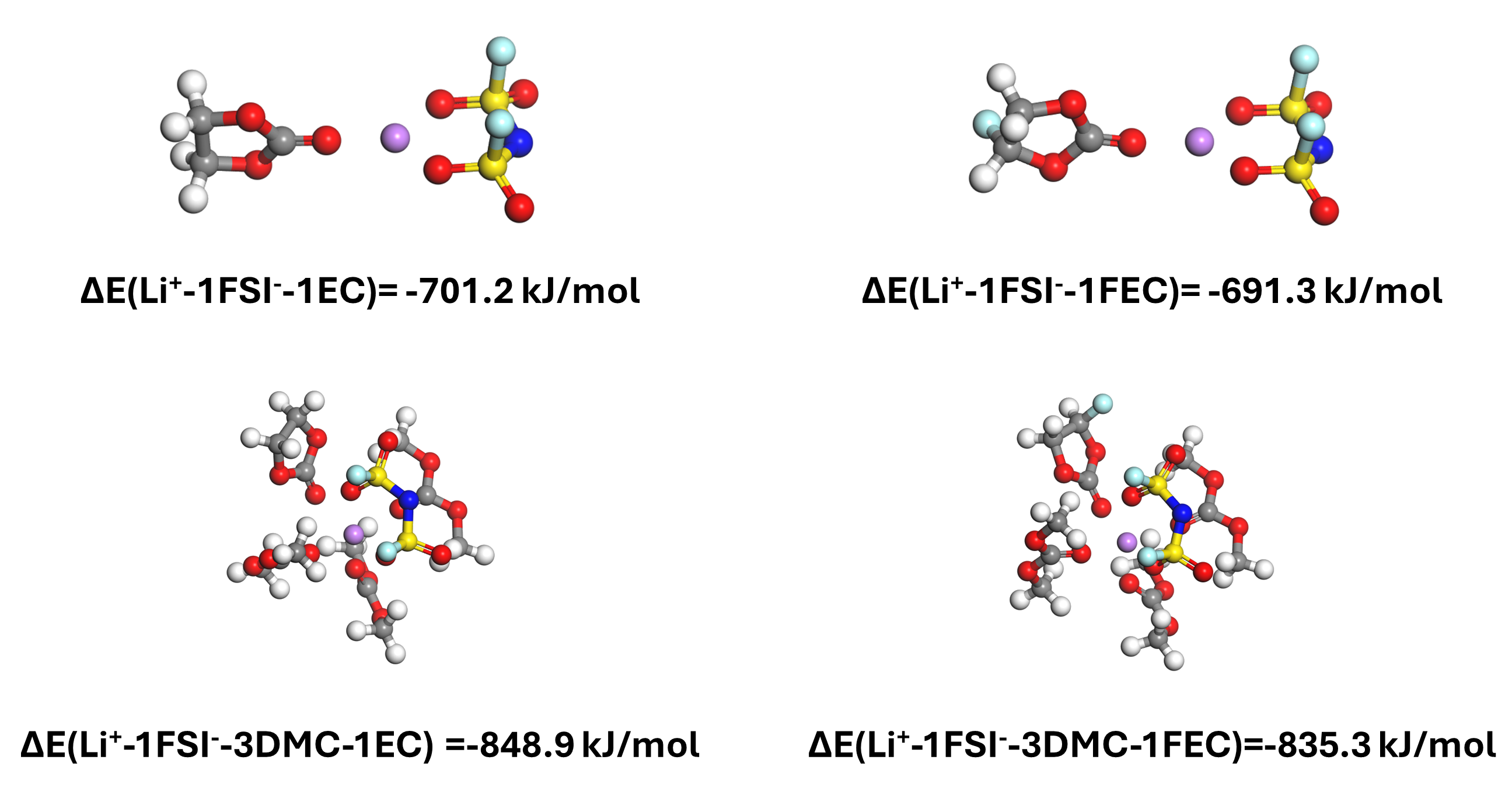


**Figure S26.** Solvation energy of (a) Li^+^-1FSI^-^-1EC, (b) Li^+^-1FSI^-^-1FEC, (c) Li^+^-1FSI^-^-3DMC-1EC and (d) Li^+^-1FSI^-^-3DMC-1FEC configuration.


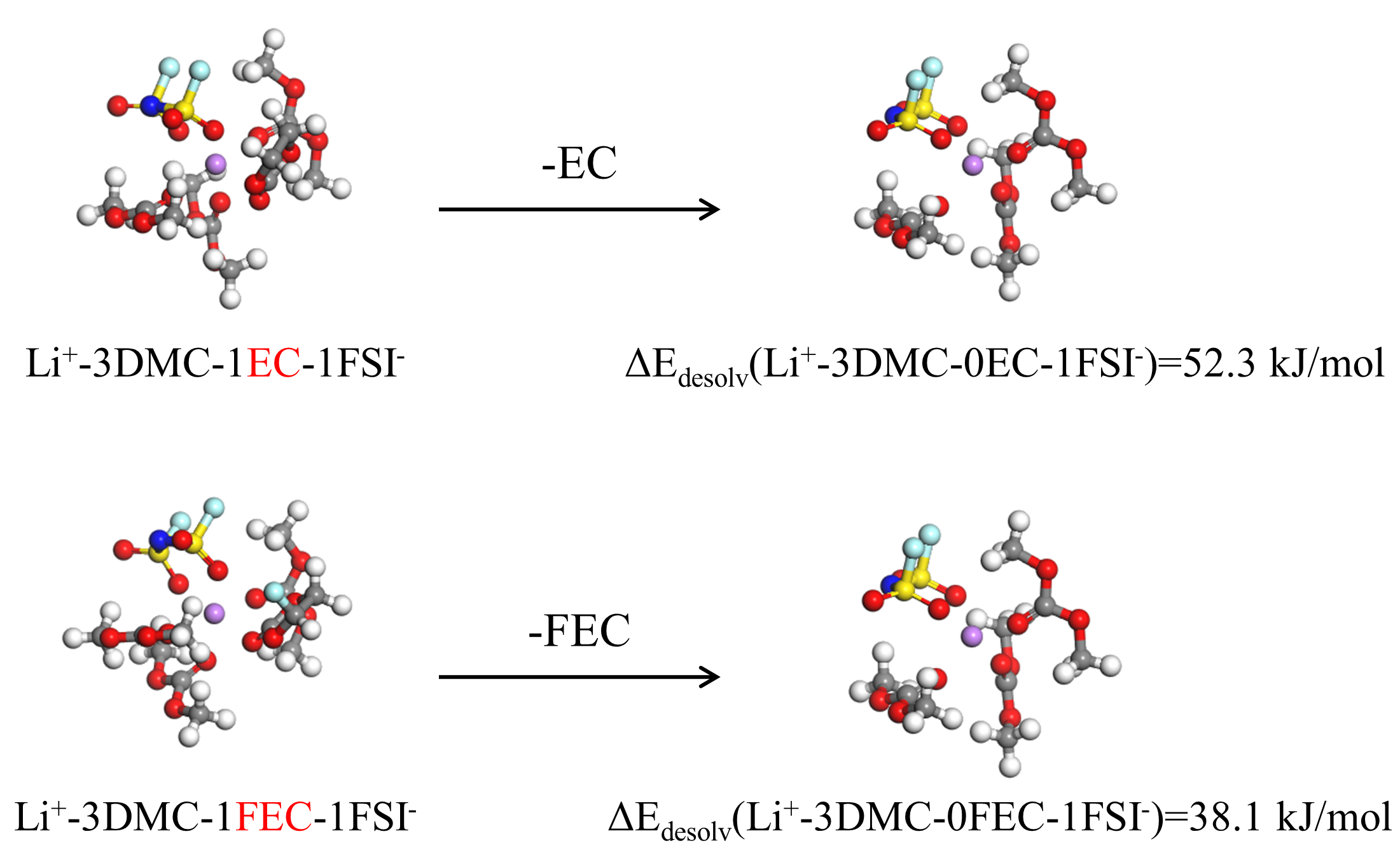


**Figure S27.** The desolvation energy of Li^+^-3DMC-1EC-1FSI^-^ and Li^+^-3DMC-1FEC-1FSI^-^. The desolvation energy is defined as the energy required to remove one solvent molecule from the first solvation shell.


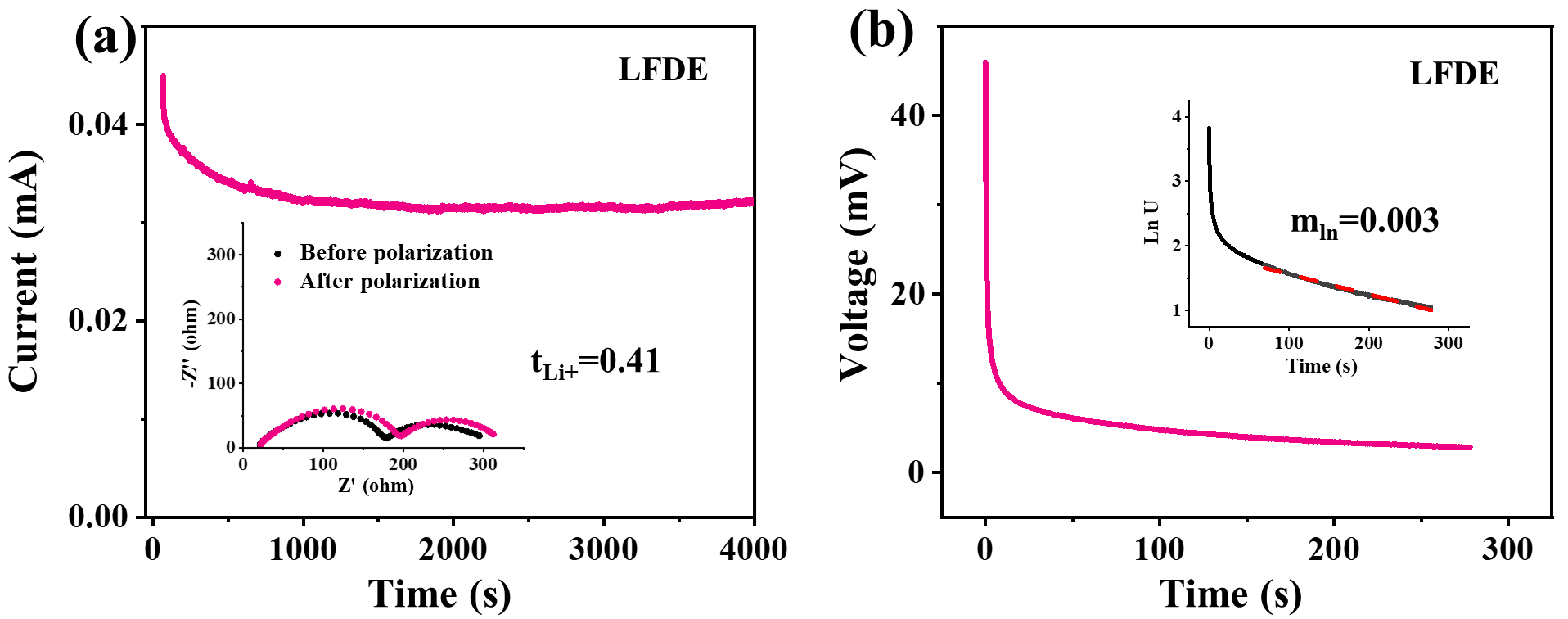


**Figure S28.** Measurements of Li^+^ transference number. (a) Chronoamperometry profile under a polarization voltage of 10 mV for LFDE electrolyte. The corresponding EIS of the initial and steady-state values of resistances before and after polarization are shown inside respectively. (b) Results of the galvanostatic pulse polarization experiment for LFDE electrolytes. The polarization time is 60 s and the polarization current density is Ip = 1 mA/cm² (corresponding to a current of Ip = 1.13 mA).


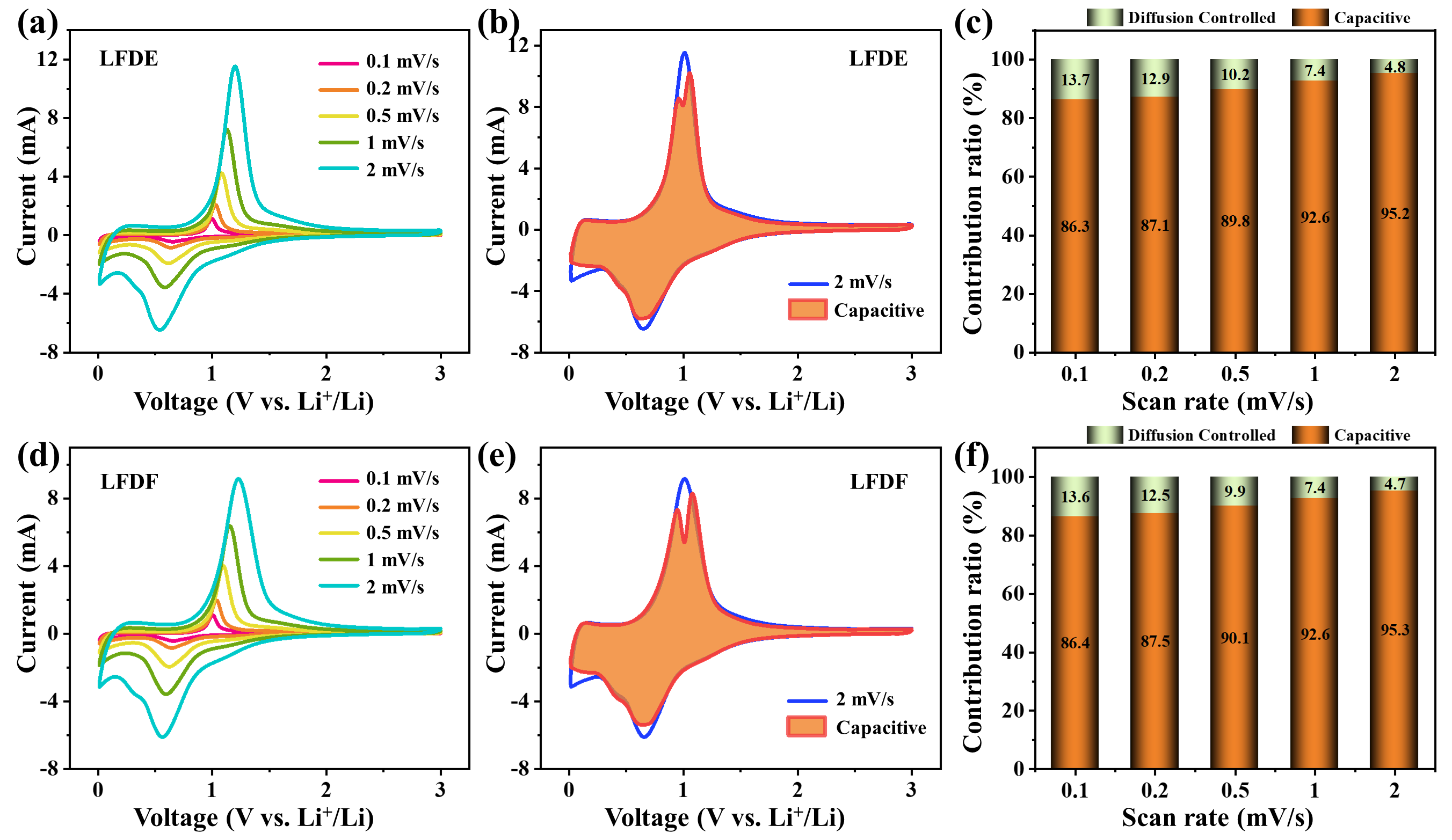


**Figure S29.** (a) CV curves of BP@C@LPO electrode at various scan rates ranging from 0.1 to 2 mV s^-1^ in LFDE; (b) The percentage of pseudocapacitance of BP@C@LPO electrode in LFDE at 2 mV s^-1^; (c) contribution ratios of diffusion controlled and pseudocapacitive currents of BP@C@LPO electrode at various scan rates in LFDE.


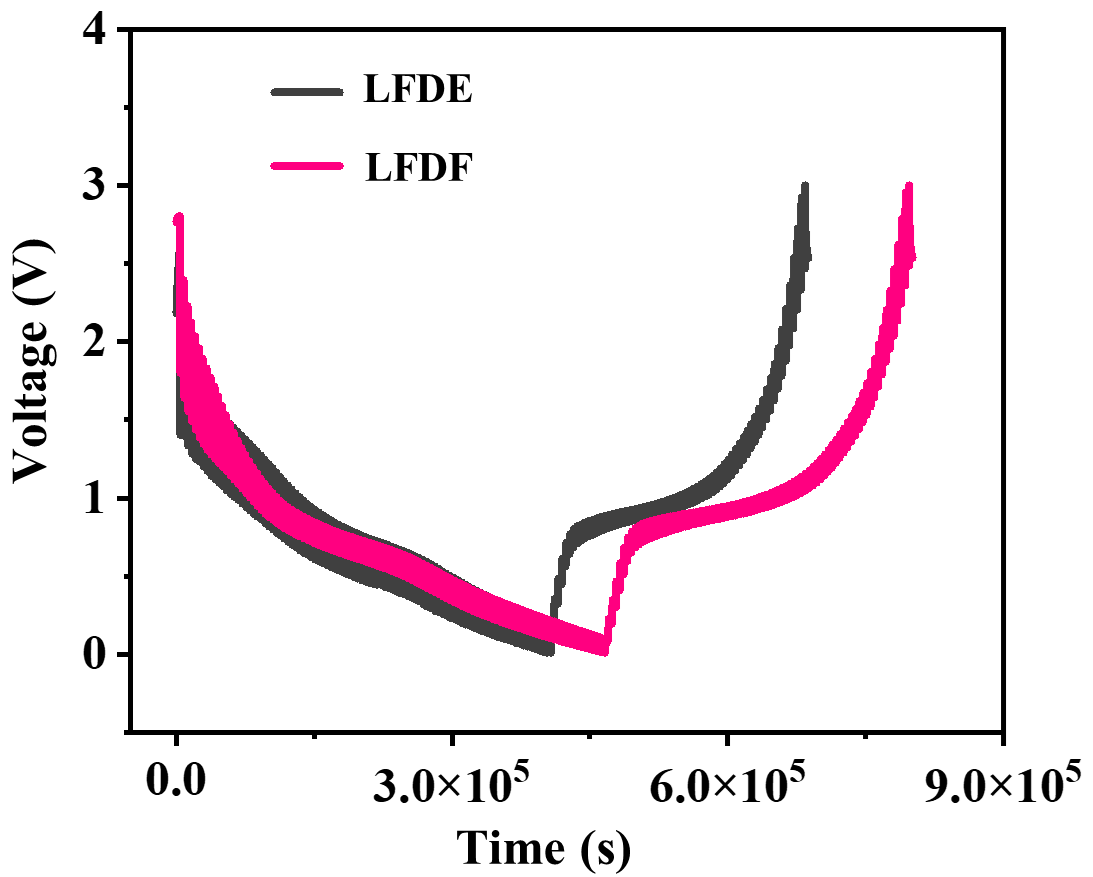


**Figure S30.** The potential response curves of BP@C@LPO electrode in LFDE and LFDF electrolytes during GITT measurements.


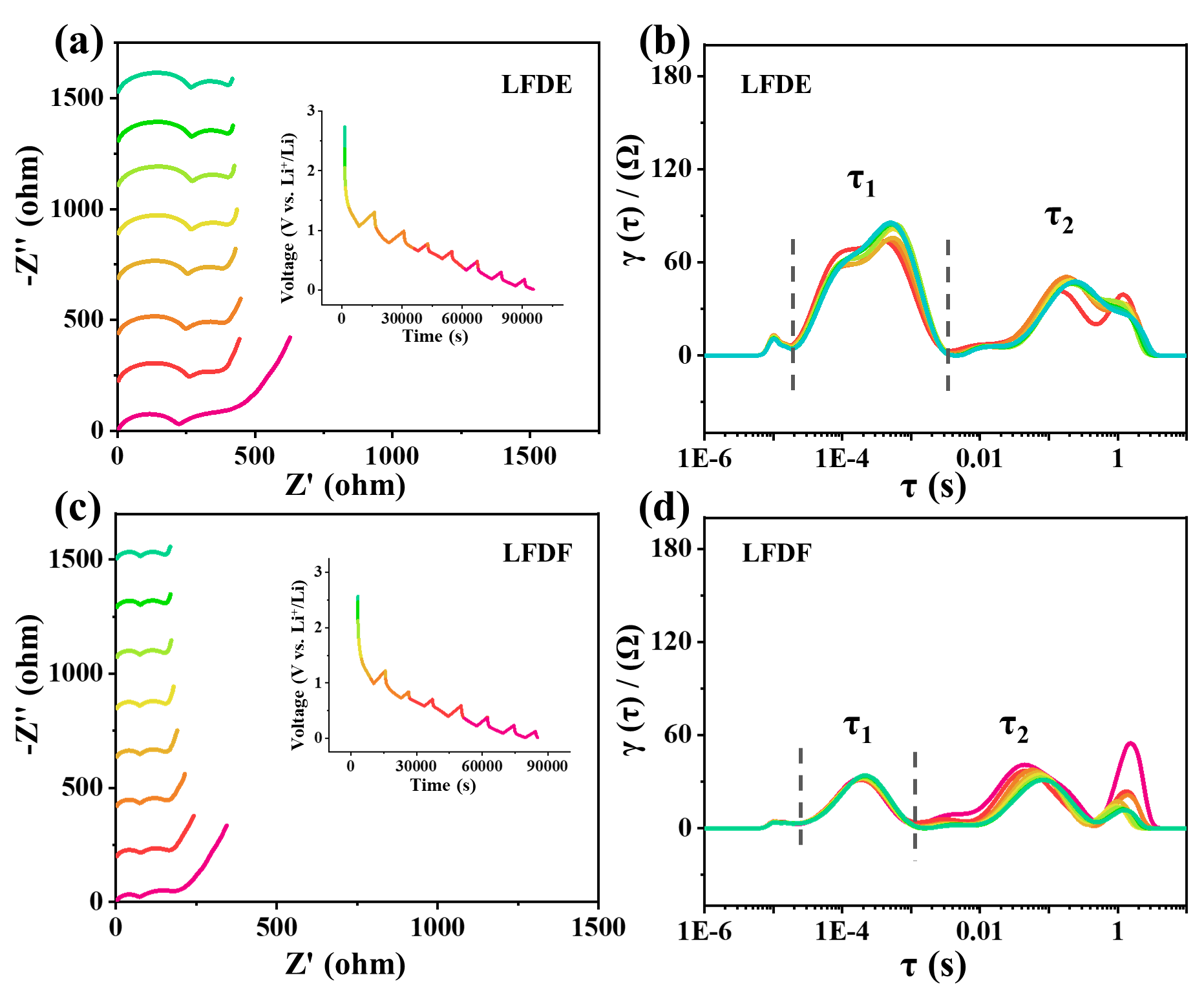


**Figure S31.** The in-situ EIS curves and DRT curves of BP@C@LPO in (a, b) LFDE and (c, d) LFDF. The inset is the voltage-time discharge curve.


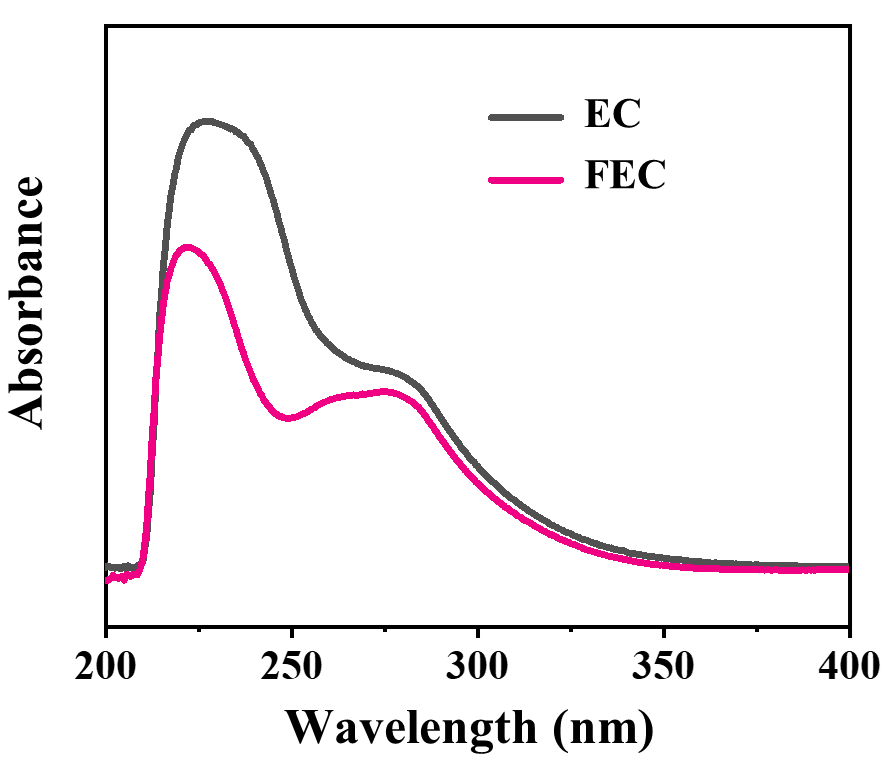


**Figure S32.** UV-visible spectra of lithium polyphosphide after dissolving in EC and FEC solvents respectively.


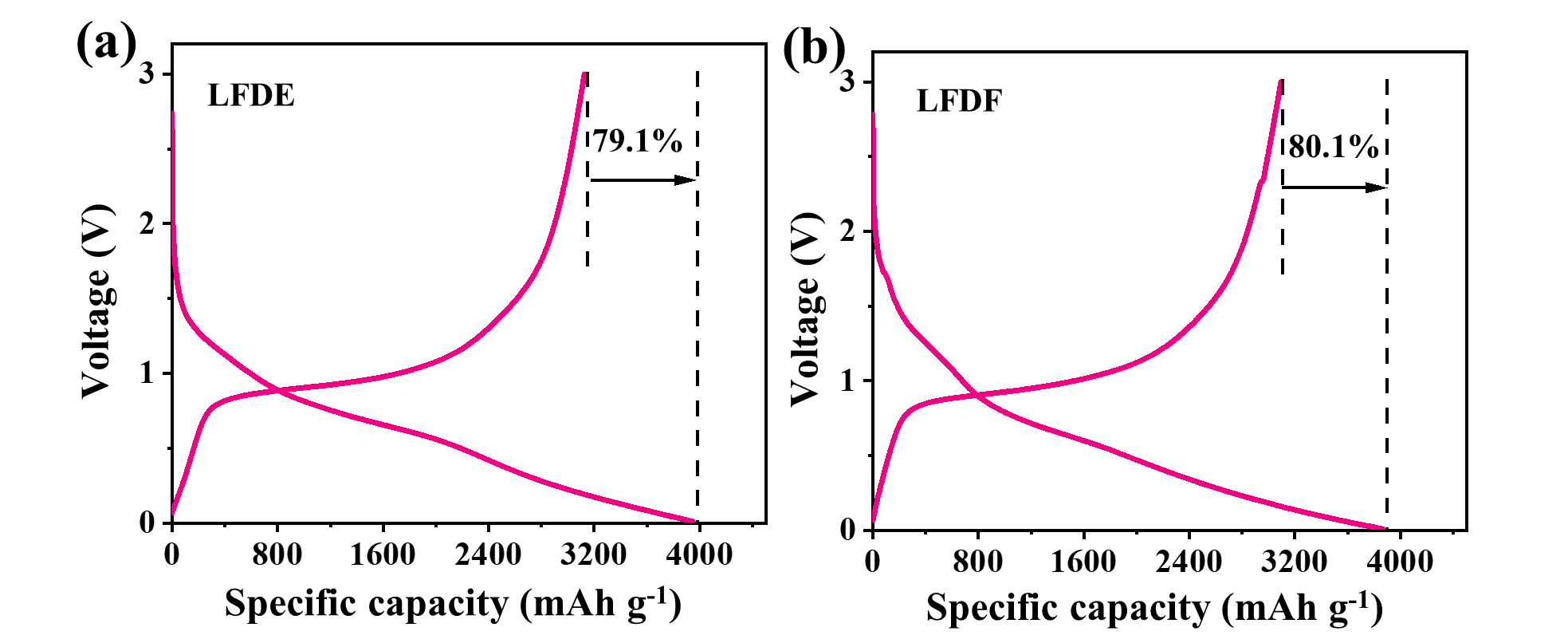


**Figure S33.** Charge/discharge curves conducted at 0.1C of BP@C@LPO electrode using (a) LFDE and (b) LFDF electrolytes.


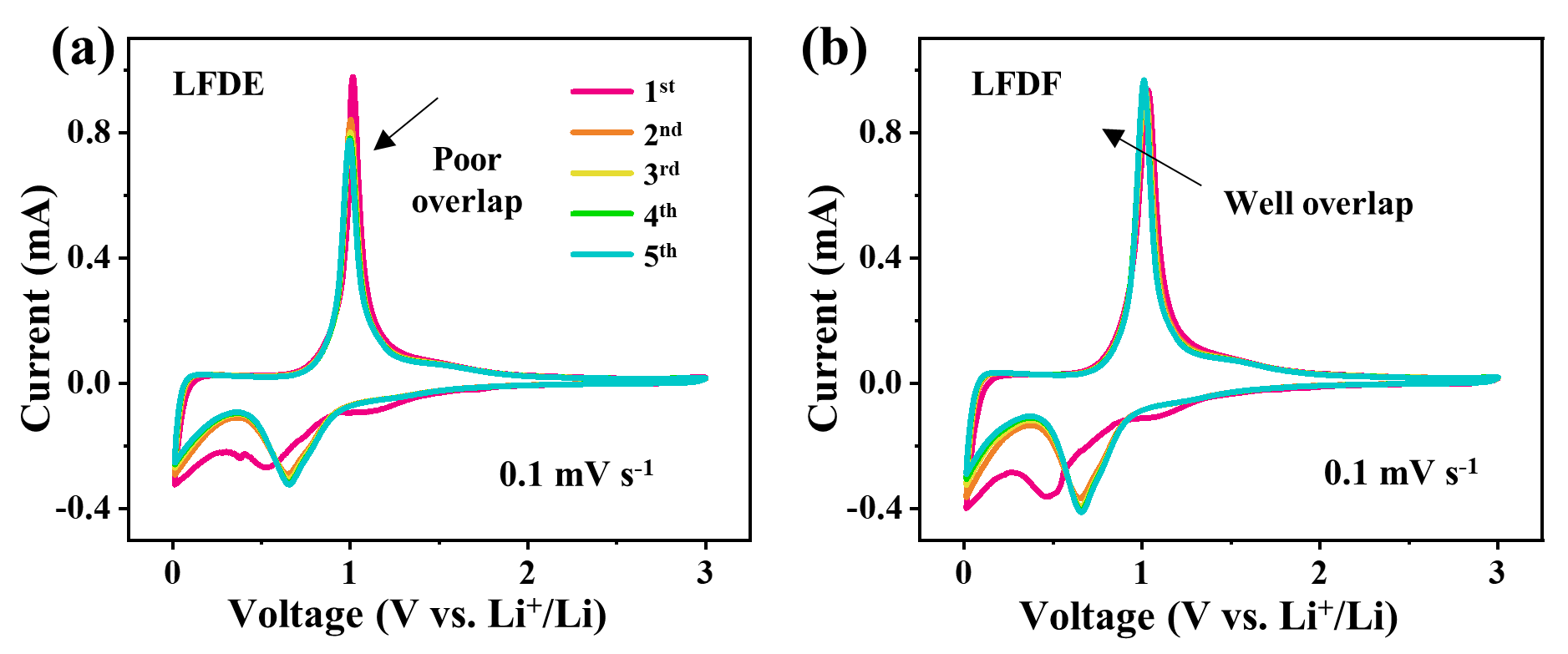


**Figure S34.** CV curves of BP@C@LPO electrode at 0.1 mV s^-1^ in (a) LFDE and LFDF.


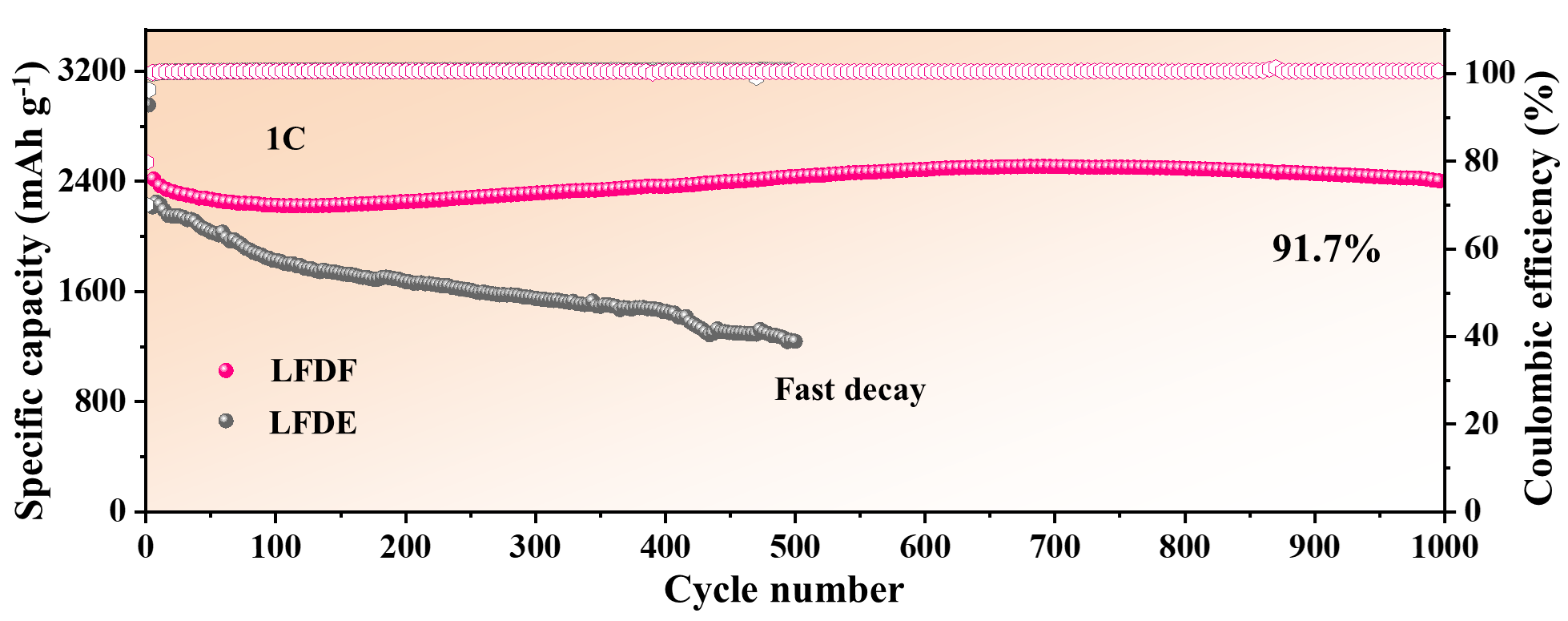


**Figure S35.** The long cycling performance conducted at 1C of BP@C@LPO using LFDE and LFDF electrolytes.


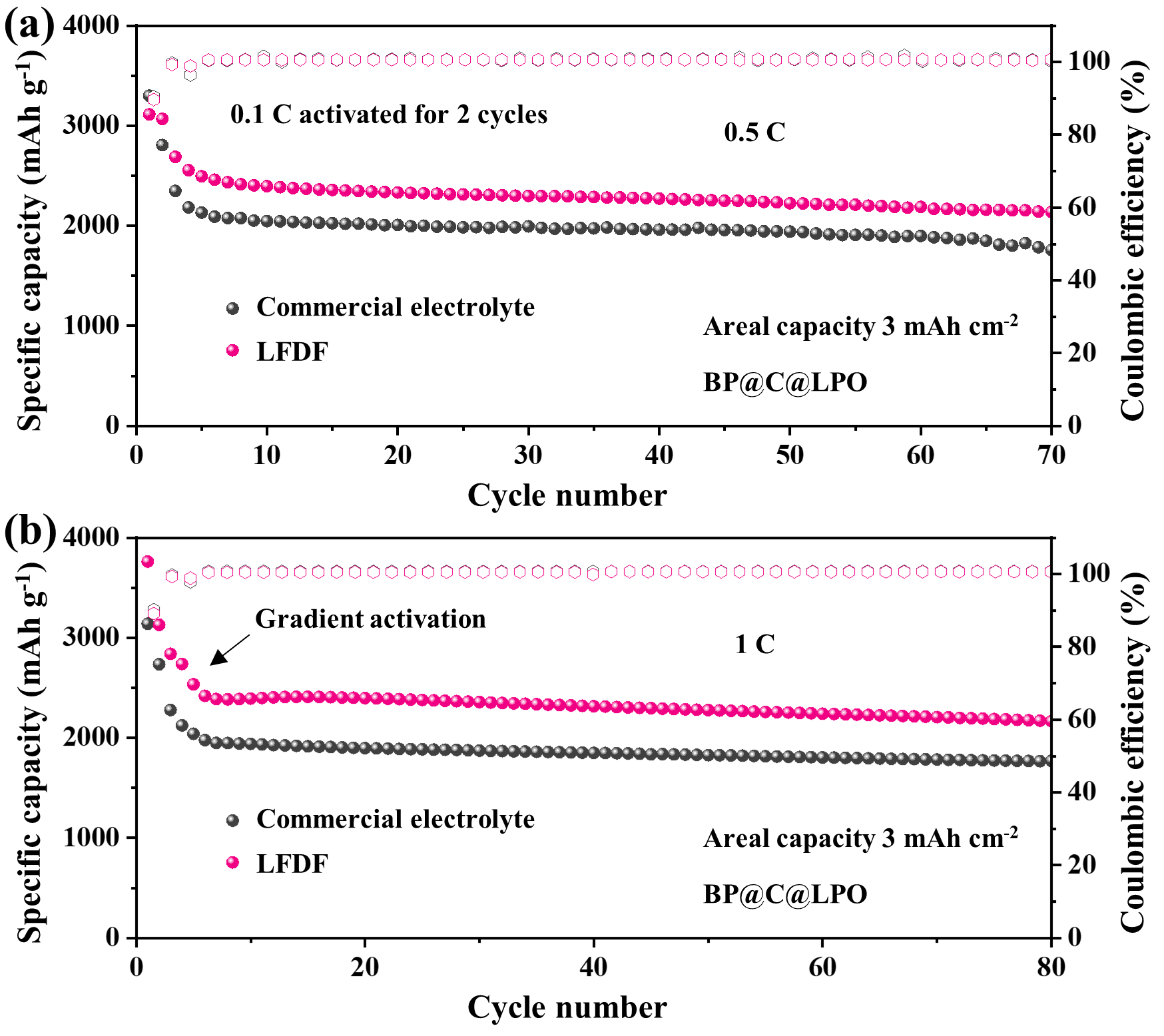


**Figure S36.** The cycling performance conducted at (a) 0.5C and (b) 1C of BP@C@LPO electrode with the areal capacity of 3 mAh cm^-2^ using LFDF and commercial electrolyte. After activation at 0.1C, the BP@C@LPO electrode used LFDF with an areal capacity of 3 mAh cm^-2^ demonstrates superior performance, delivering a capacity of 2555.1 mAh g^-1^ at 0.5C, significantly outperforming the 2183.5 mAh g^-1^ achieved with commercial electrolyte. At a slightly higher rate of 1C, the BP@C@LPO electrode with LFDF still delivers an impressive capacity of 2419.7 mAh g⁻¹, whereas the electrode with the commercial electrolyte only reaches 1977.3 mAh g⁻¹. These results clearly demonstrate that the LFDF electrolyte enables excellent high-rate capability while supporting high mass loading, making it particularly suitable for practical high-energy-density battery applications.


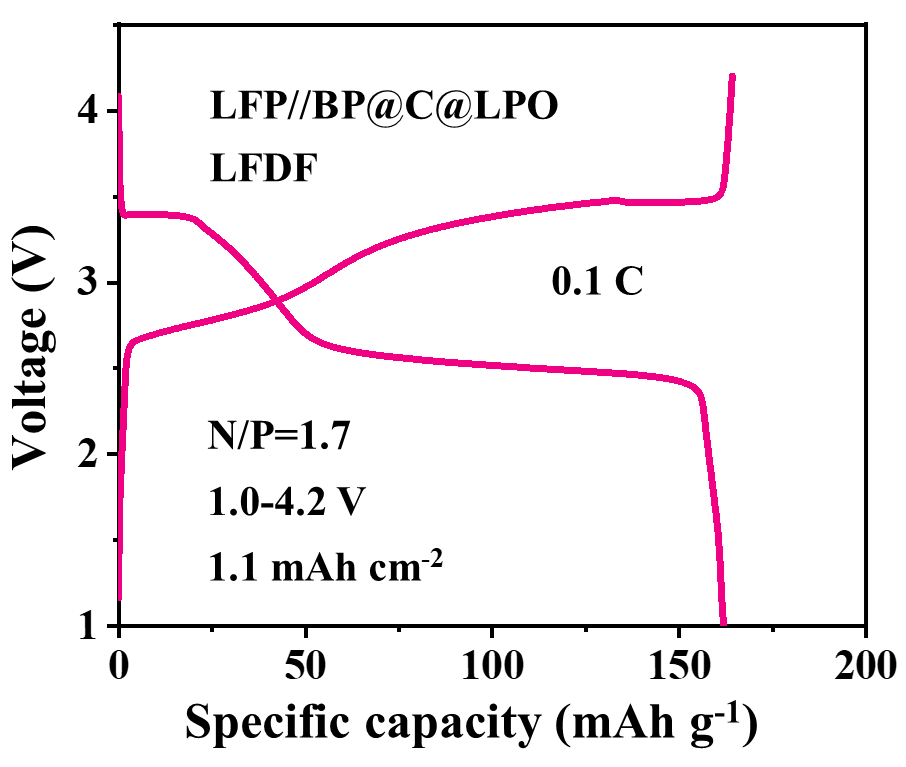


**Figure S37.** Charge/discharge curves conducted at 0.1C of LFP//BP@C@LPO full cell using LFDF electrolyte. The BP@C@LPO anode was prelithiated for 5 cycles at 0.1C.


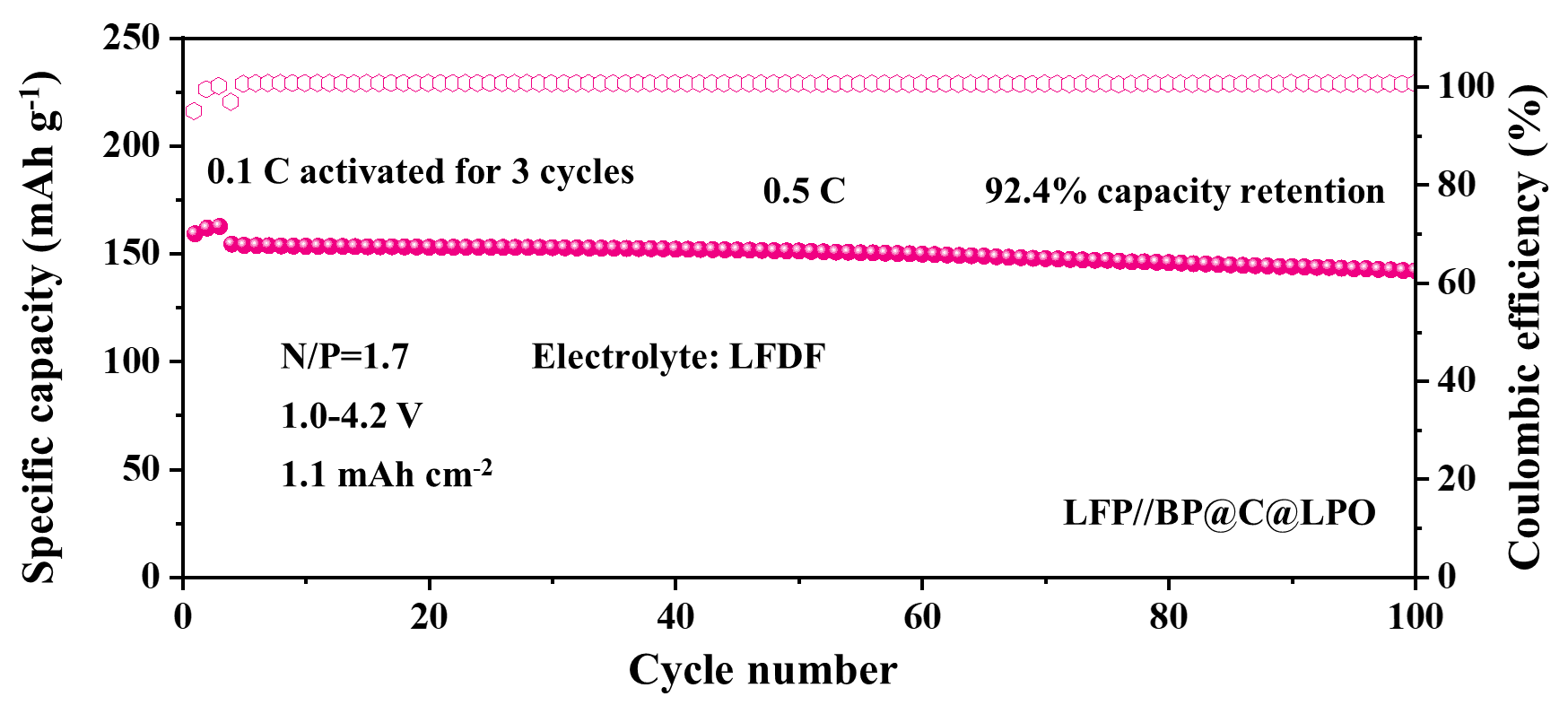


**Figure S38.** The long cycling performance conducted at 0.5C of LFP//BP@C@LPO full cell using LFDF electrolyte. LFP refers to lithium iron phosphate (LiFePO_4_) and the capacity is calculated based on cathode.


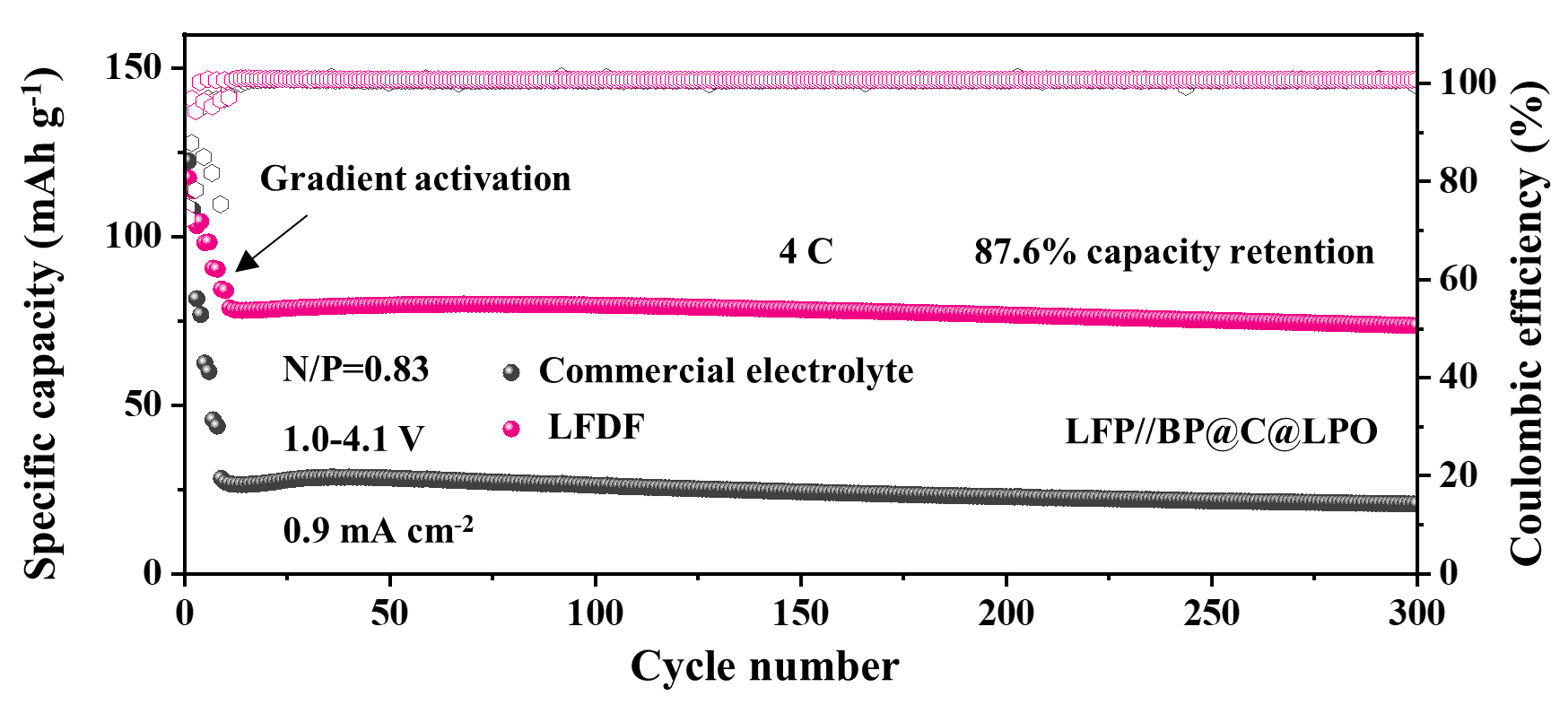


**Figure S39.** The long cycling performance conducted at 4C of LFP//BP@C@LPO full cell using commercial and LFDF electrolyte.


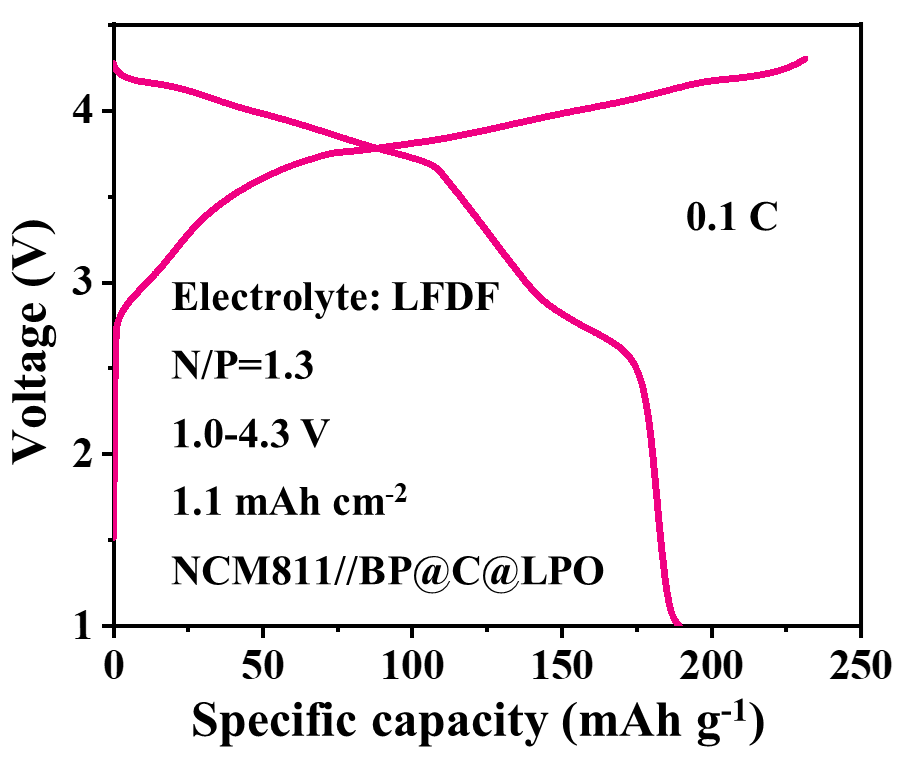


**Figure S40**. Charge/discharge curves conducted at 0.1C of NCM811//BP@C@LPO full cell using LFDF electrolyte. The NCM811//BP@C@LPO with LFDF delivers a capacity of 189.3 mAh g^-1^ at 0.1 C.


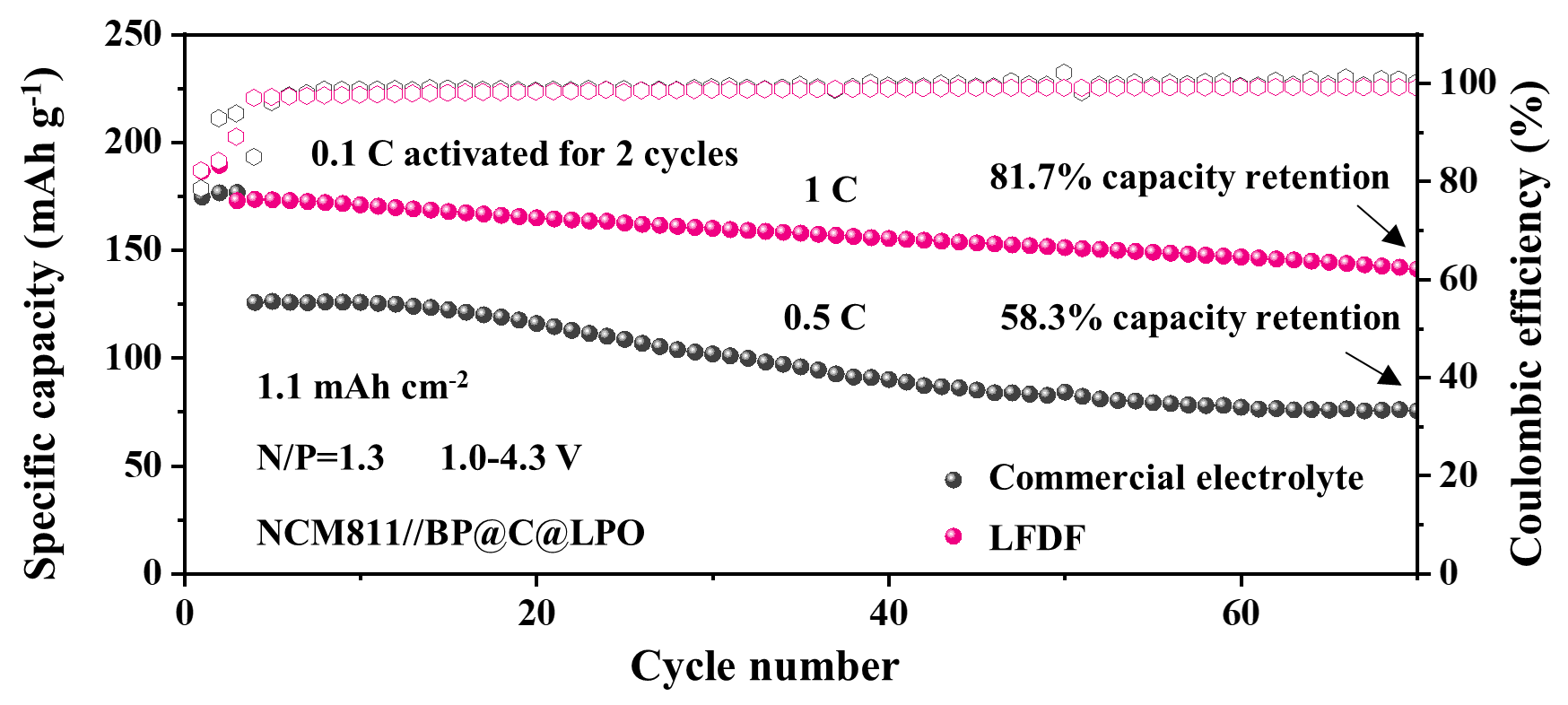


**Figure S41**. The cycling performance of NCM811//BP@C@LPO full cell using LFDF electrolyte conducted at 1C and commercial electrolyte at 0.5C. The NCM811//BP@C@LPO with LFDF delivers a capacity of 173 mAh g^-1^ at 1 C, which is significantly higher than the one with commercial electrolyte conducted at lower rate of 0.5C. The NCM811//BP@C@LPO with LFDF retains its capacity of 81.7% after 70 cycles while only 58.3% for the one with commercial electrolyte.


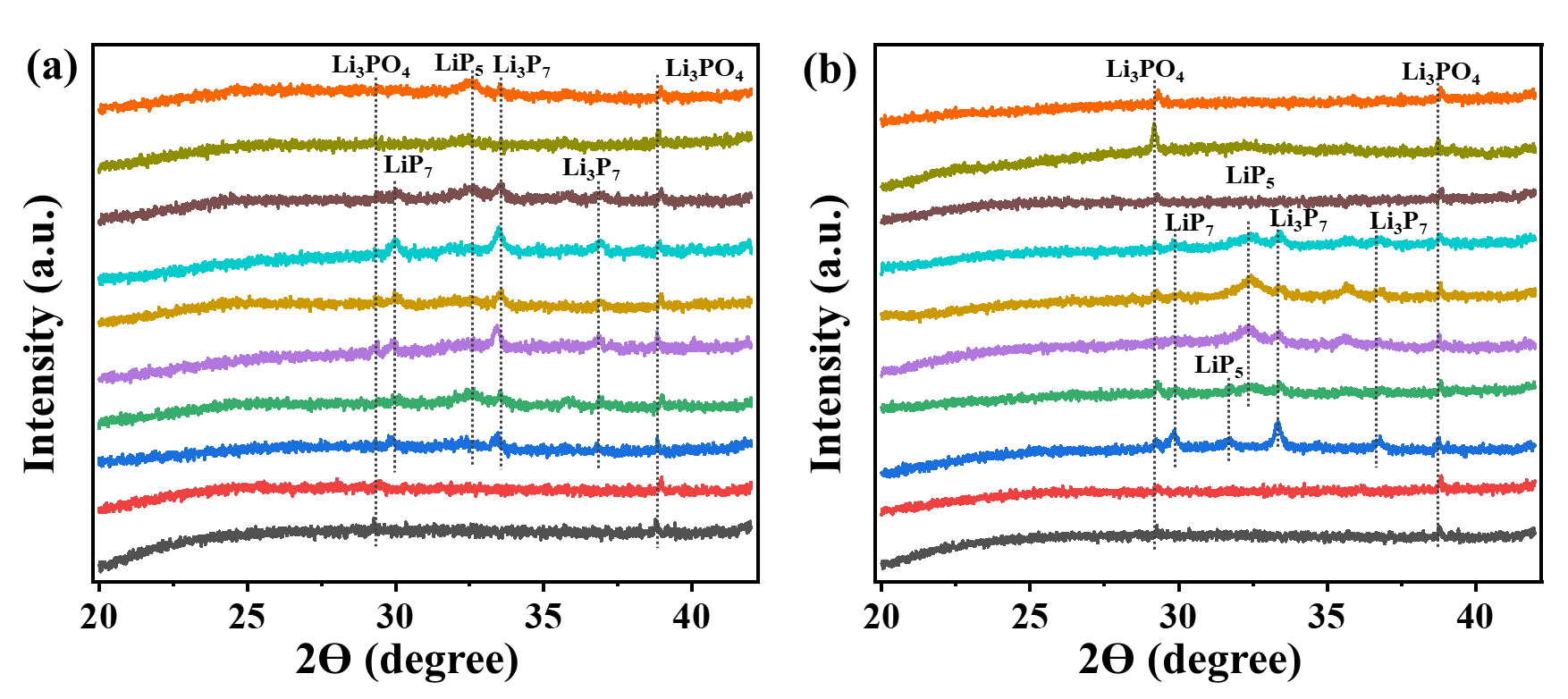


**Figure S42.** The ex-situ XRD patterns of (a) BP@C@LPO in (a) LFDE and (b) LFDF. From the bottom to the top, the charging and discharging conditions of electrodes are as follows: initial electrode, after being discharged to 1 V, 0.7 V, 0.5 V, 0.2 V, 0.01 V, and after being charged to 0.5 V, 1 V, 1.5V, and 3 V, respectively. The peaks located at around 29.3^o^ and 38.8 ^o^ are attributed to the microcrystal Li_3_PO_4_ in the original material (PDF#71-1528). The peak centered at 30.1° is assigned to LiP_7_ (PDF#73-1162), while the peaks at 31.7° and 32.6° are attributed to LiP_5_ (PDF#73-1161), and those at 33.5° and 36.8° to Li_3_P_7_ (PDF#77-2425).


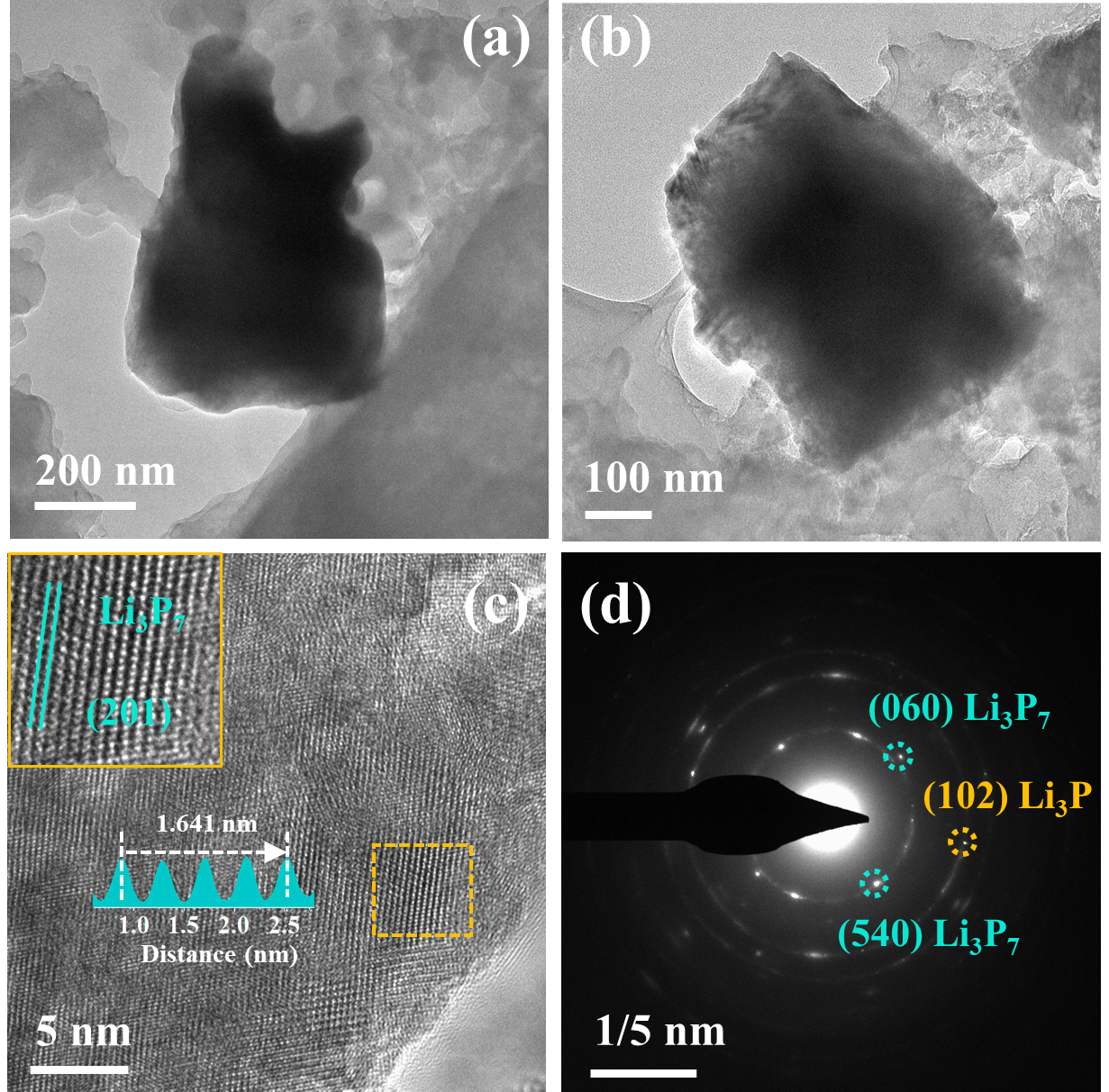


**Figure S43.** TEM images of BP@C@LPO using (a) LFDE and (b) LFDF after discharged to 0.01 V; (c) The HRTEM image and (d) corresponding SAED of BP@C@LPO using LFDF after discharged to 0.01 V. The lattice spacing of 0.410 nm in (c) can be attributed to (201) of Li_3_P_7_. As for SAED pattern in (d), the lattice spacing of 0.175, 0.157 and 0.264nm can be assigned to (060), (540) and (102) of Li_3_P_7_ and Li_3_P. Notably, while Li_3_P was detected by TEM, it was not observed in XRD, likely due to the sensitivity of XRD to long-range periodic structures, which require sufficient crystallinity and size to produce clear diffraction signals. In contrast, TEM’s higher spatial resolution allows the detection of Li_3_P even at lower concentrations.


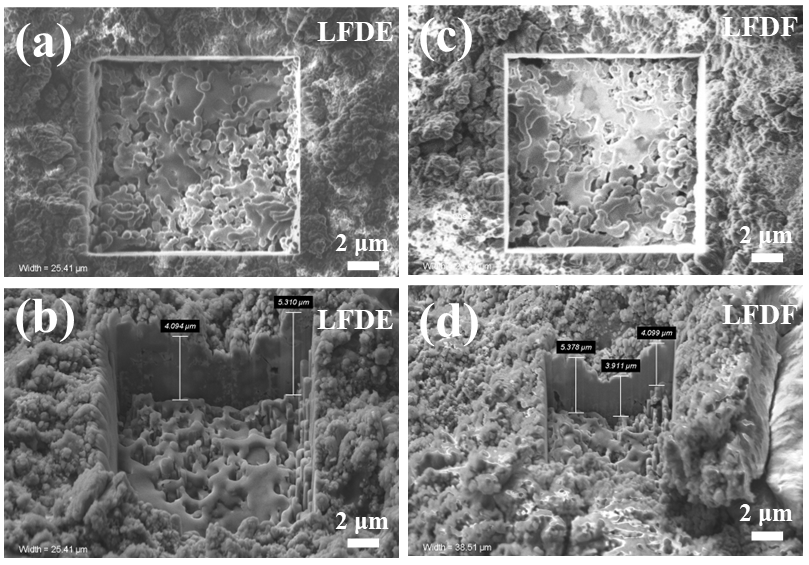


**Figure S44.** Electron microscopy image of BP@C@LPO after discharged to 0.01 V in (a, b) LFDE and (c, d) LFDF.


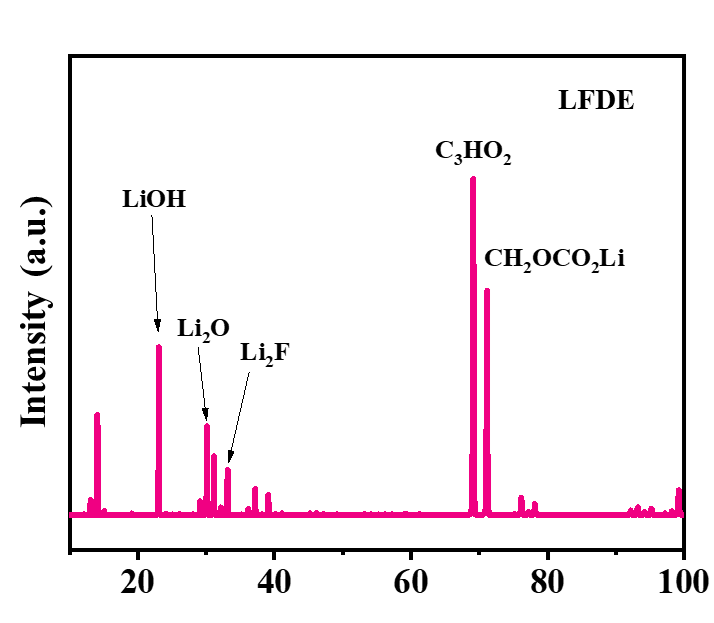


**Figure S45.** Mass spectrum of BP@C@LPO in LFDE electrolyte after discharged to 0.01 V collected in positive mode.


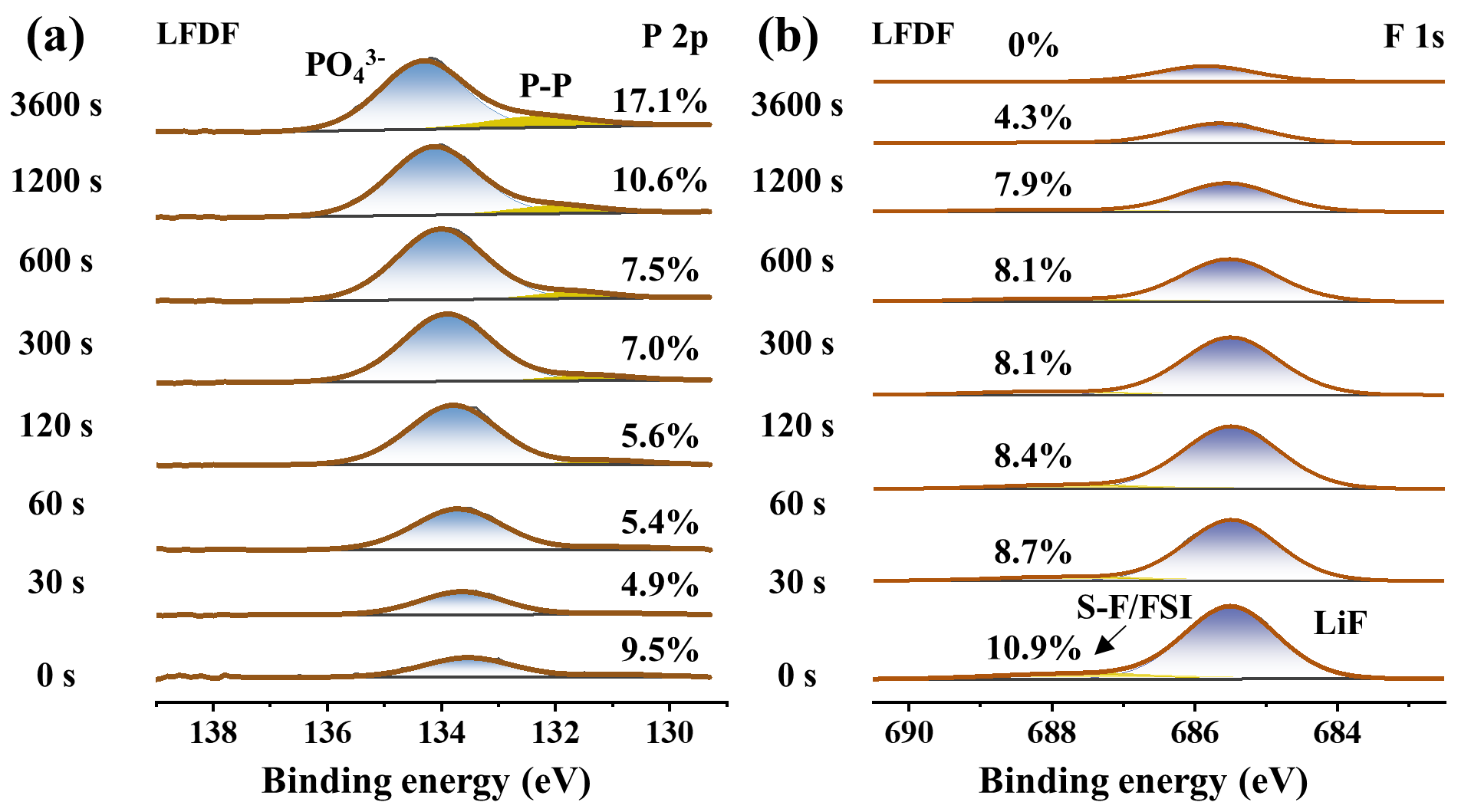


**Figure S46.** (a) P 2p and (b) F 1s spectra at different etching times of BP@C@LPO using LFDF. The peaks at 133.6 and 132.1 eV are attributed to PO₄³⁻ and P-P components, respectively. ^[12]^ Peaks observed at 687.8 and 685.1 eV correspond to S-F/FSI and LiF components. ^[12b, 13]^


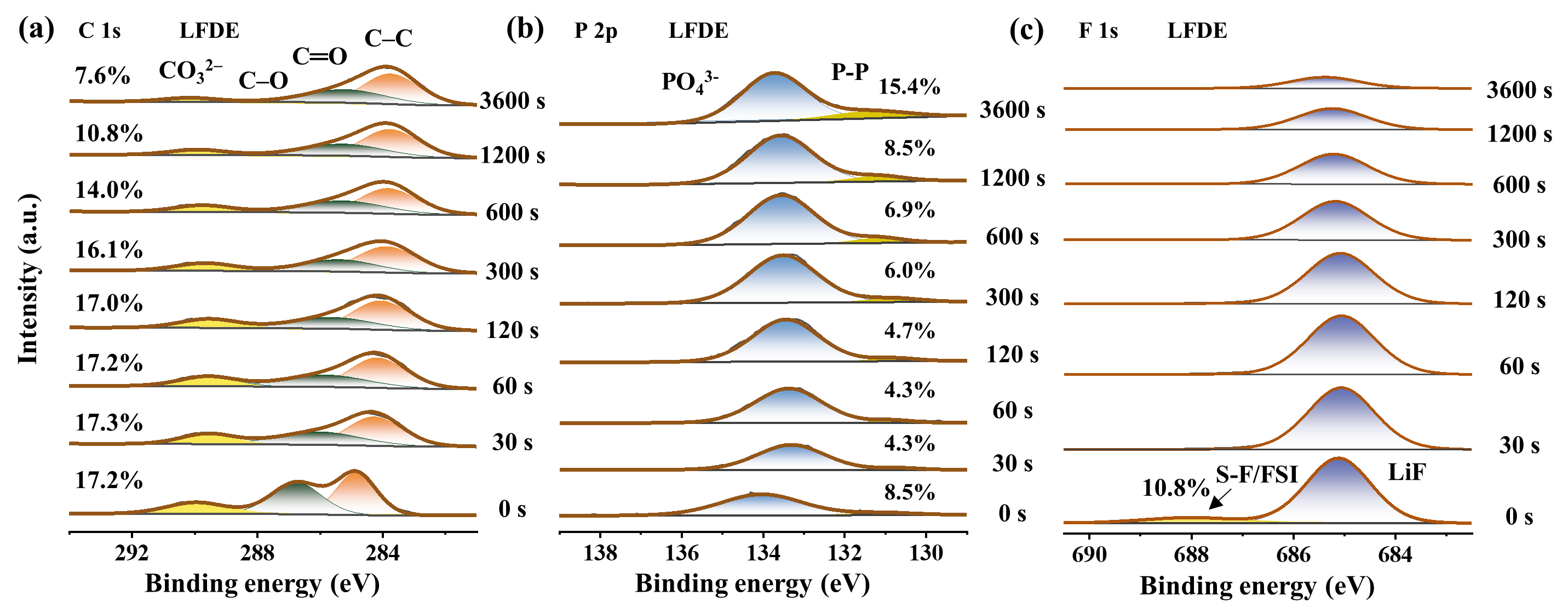


**Figure S47.** C 1s, P 2p and F 1s spectra at different etching times of BP@C@LPO using LFDE after discharged to 0.01 V.


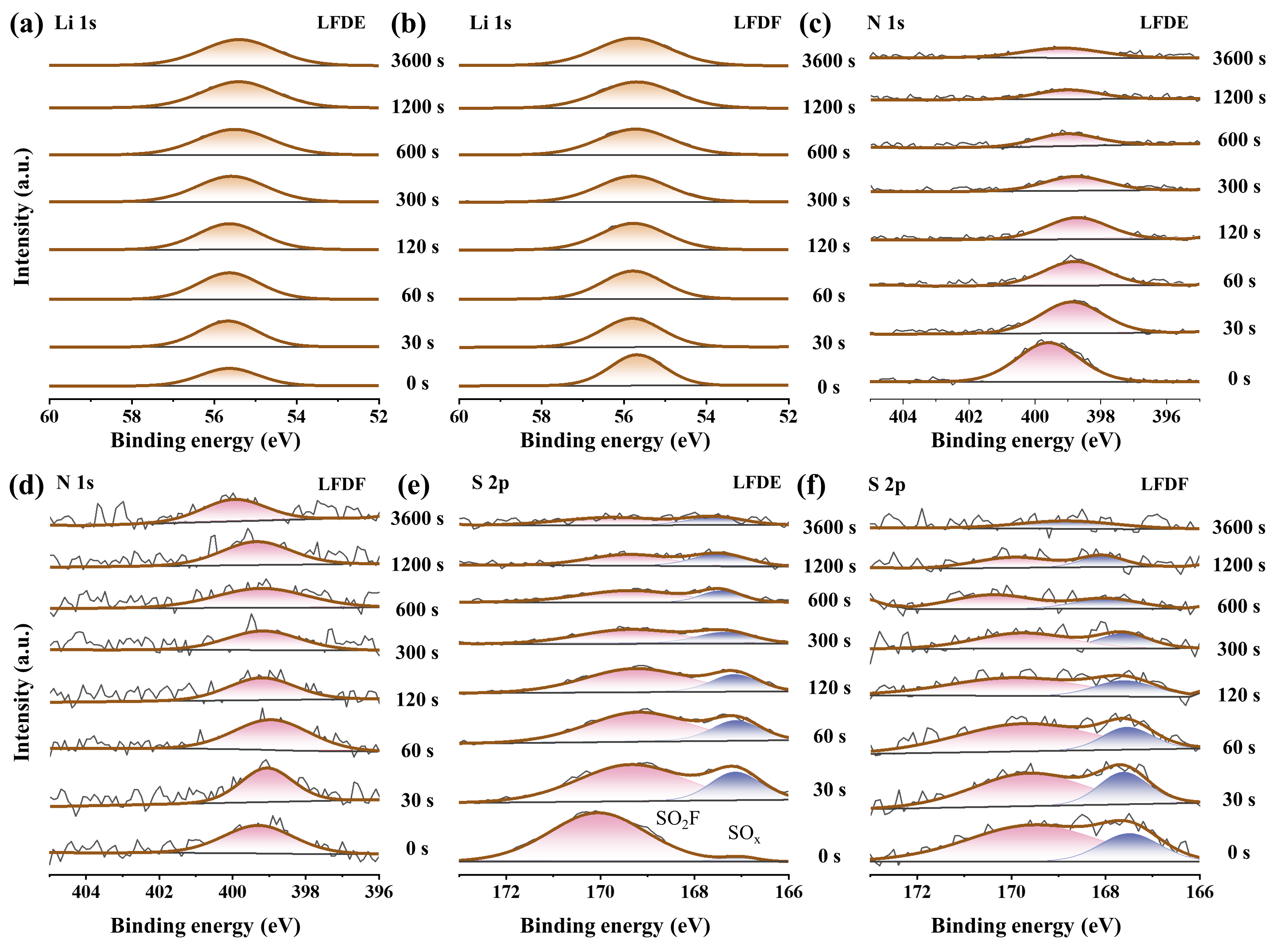


**Figure S48.** Li 1s, N 1s and S 2p spectra at different etching times of BP@C@LPO using LFDE and LFDF after discharged to 0.01 V.


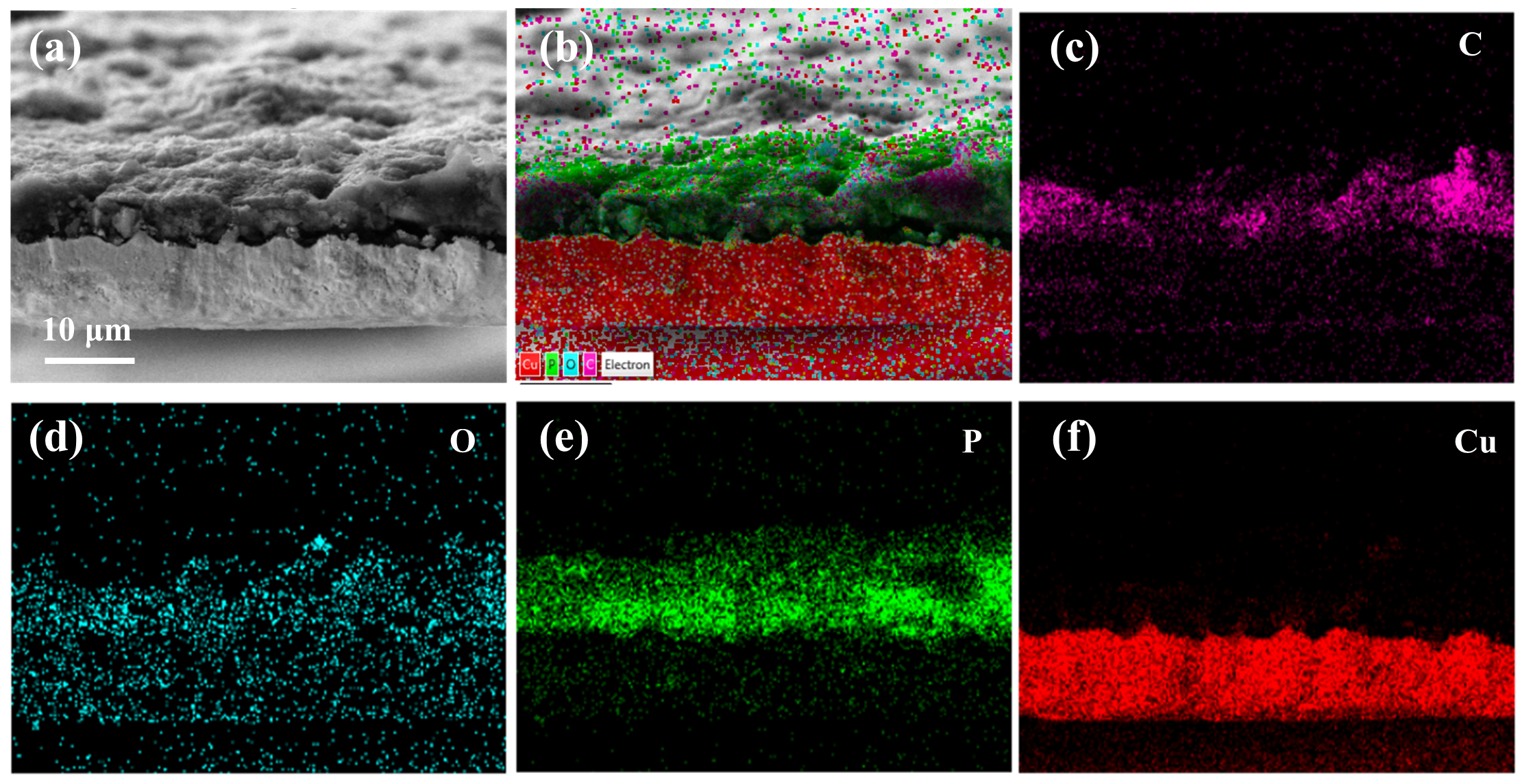


**Figure S49.** SEM images of pristine BP@C@LPO electrode and corresponding C, O, P and Cu elemental maps. The pristine electrode exhibited a uniform distribution of C, O, and P elements.


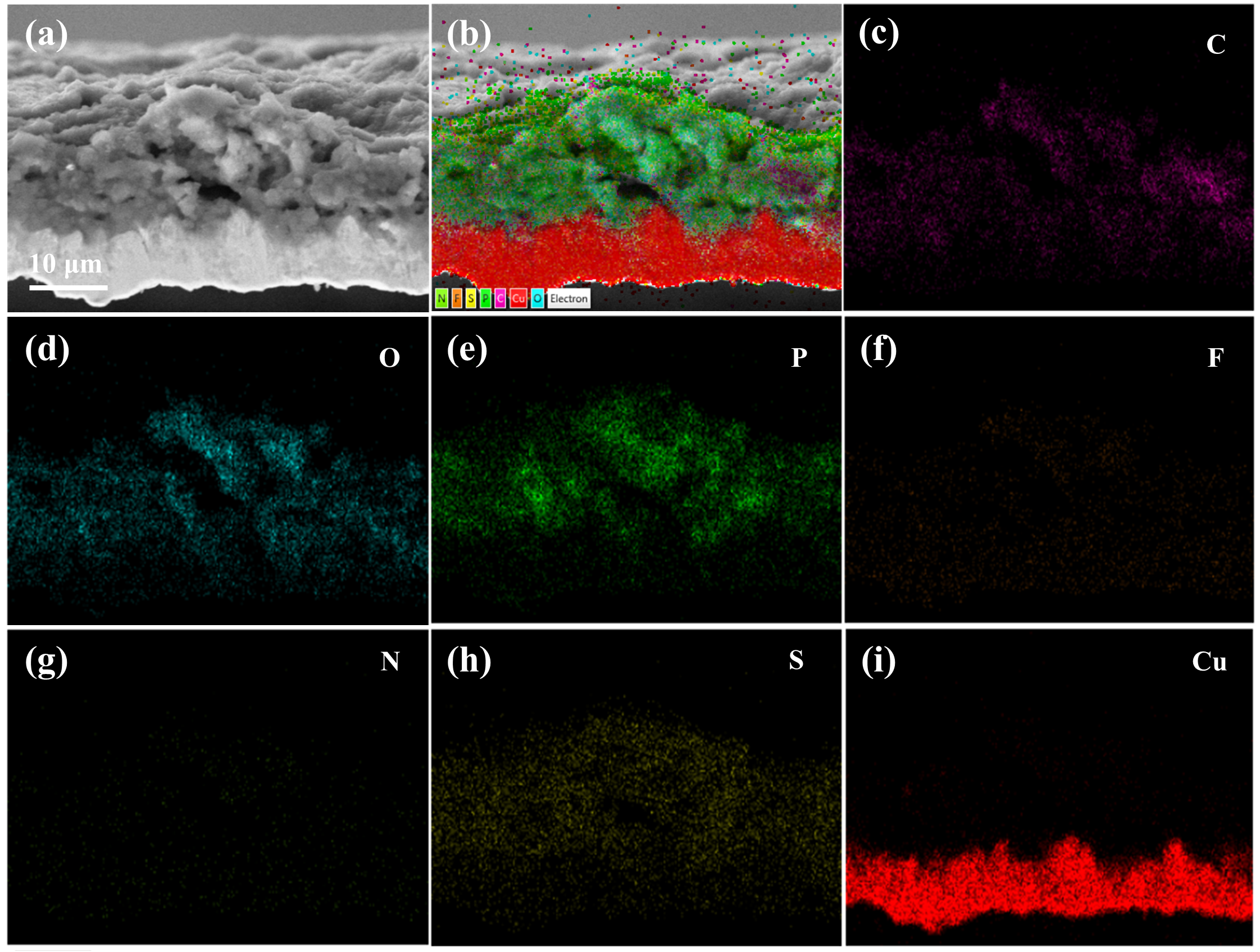


**Figure S50.** SEM images of the BP@C@LPO electrode in LFDE after 200 cycles at a 4C rate, and corresponding elemental maps for C, O, P, F, N, S, and Cu. The content of P, F, S and N is 24.4, 2.1, 12.2 and 0 wt.%.


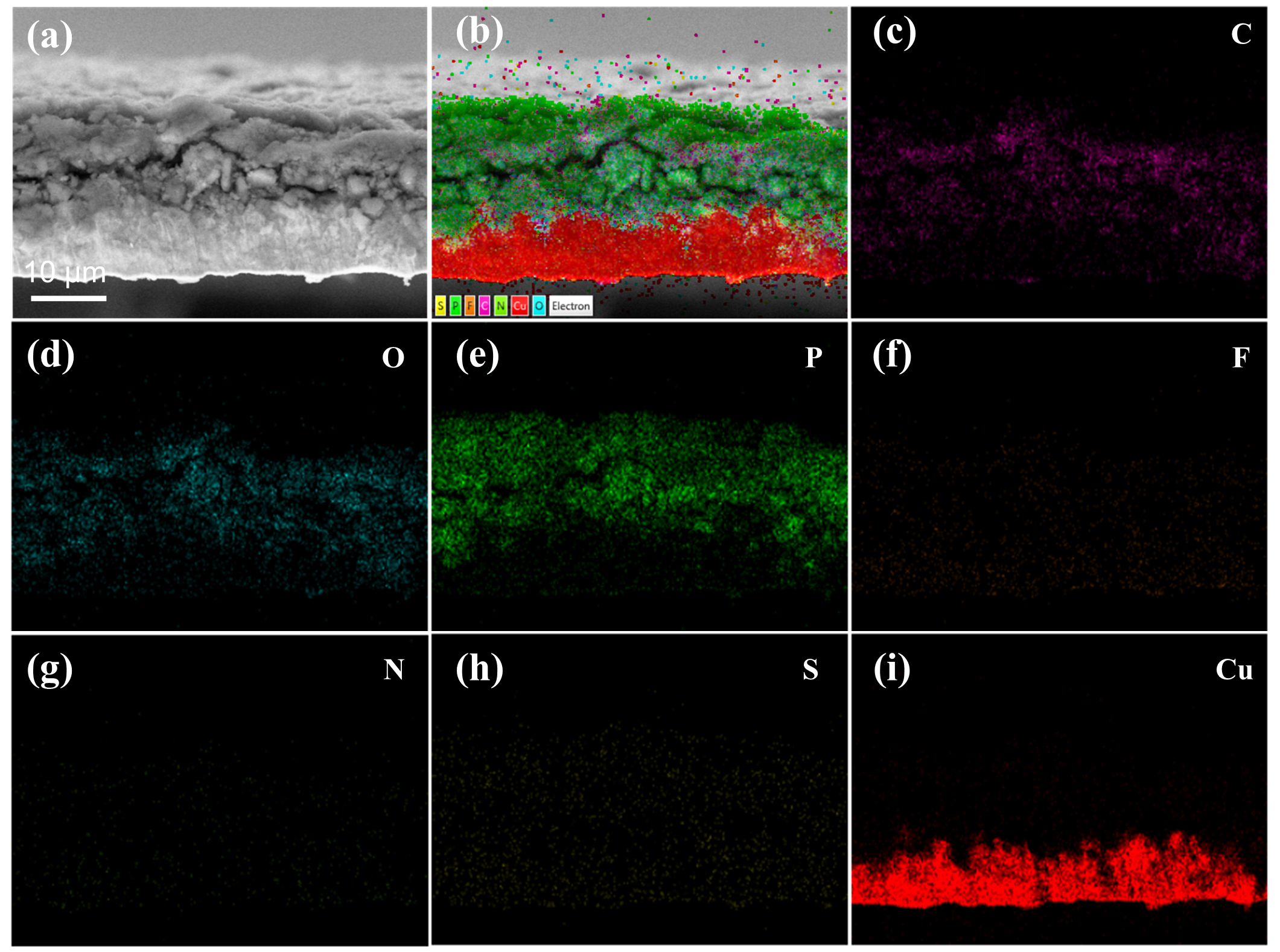


**Figure S51.** SEM images of the BP@C@LPO electrode in LFDF after 200 cycles at a 4C rate, and corresponding elemental maps for C, O, P, F, N, S, and Cu. The content of P, F, S and N is 35.4, 1.3, 1.4 and 0 wt.%.


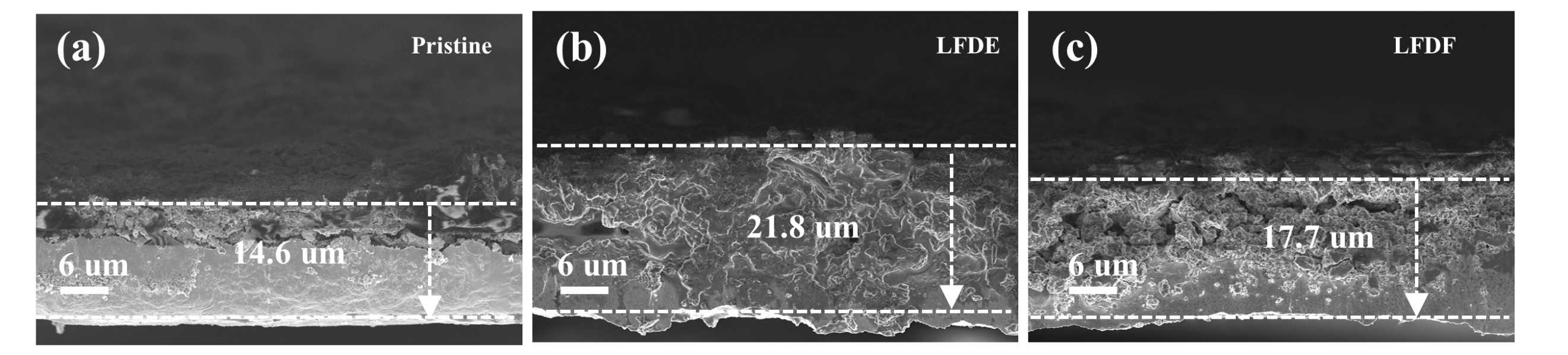


**Figure S52.** SEM cross-sectional views of the (a) pristine BP@C@LPO electrode, and the BP@C@LPO electrodes after cycling in (b) LFDE and (c) LFDF electrolytes at 4C for 200 cycles.


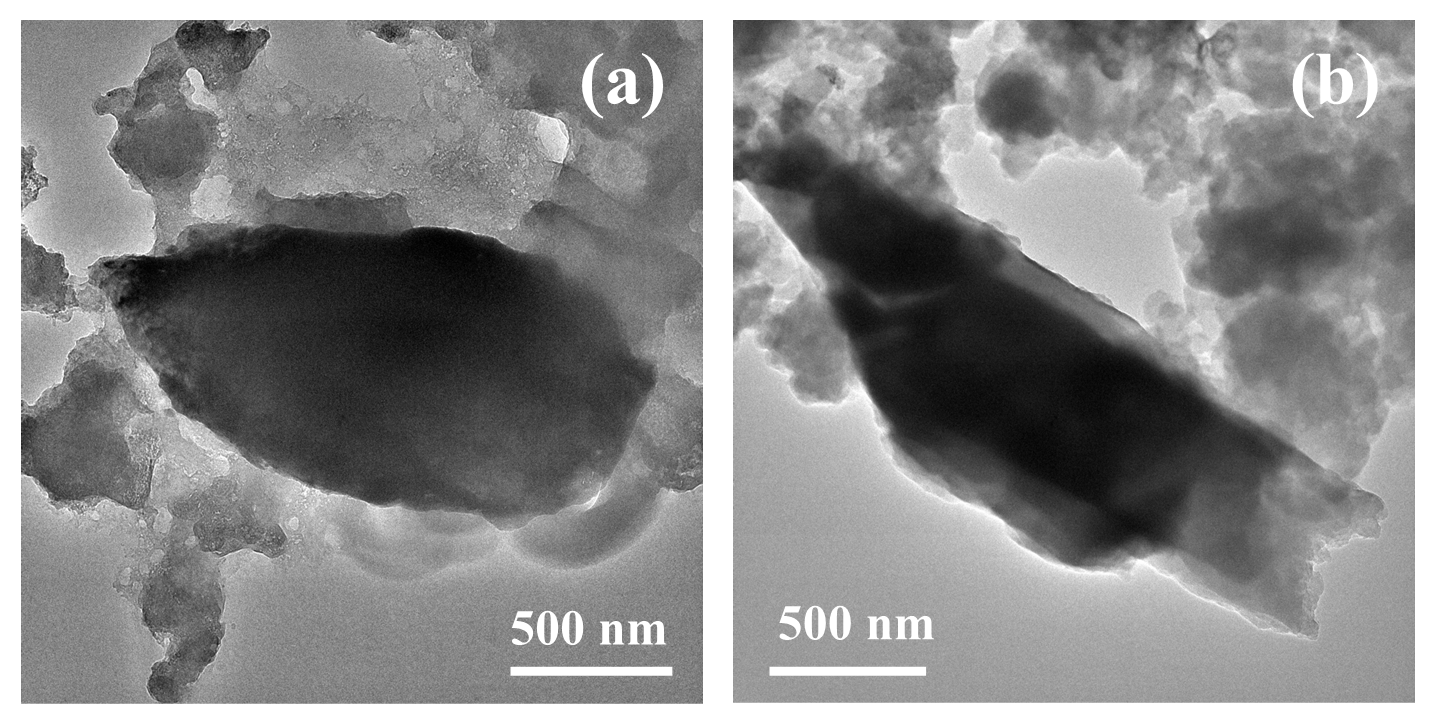


**Figure S53.** TEM images of BP@C@LPO electrode in (a) LFDE and (b) LFDF after 200 cycles.


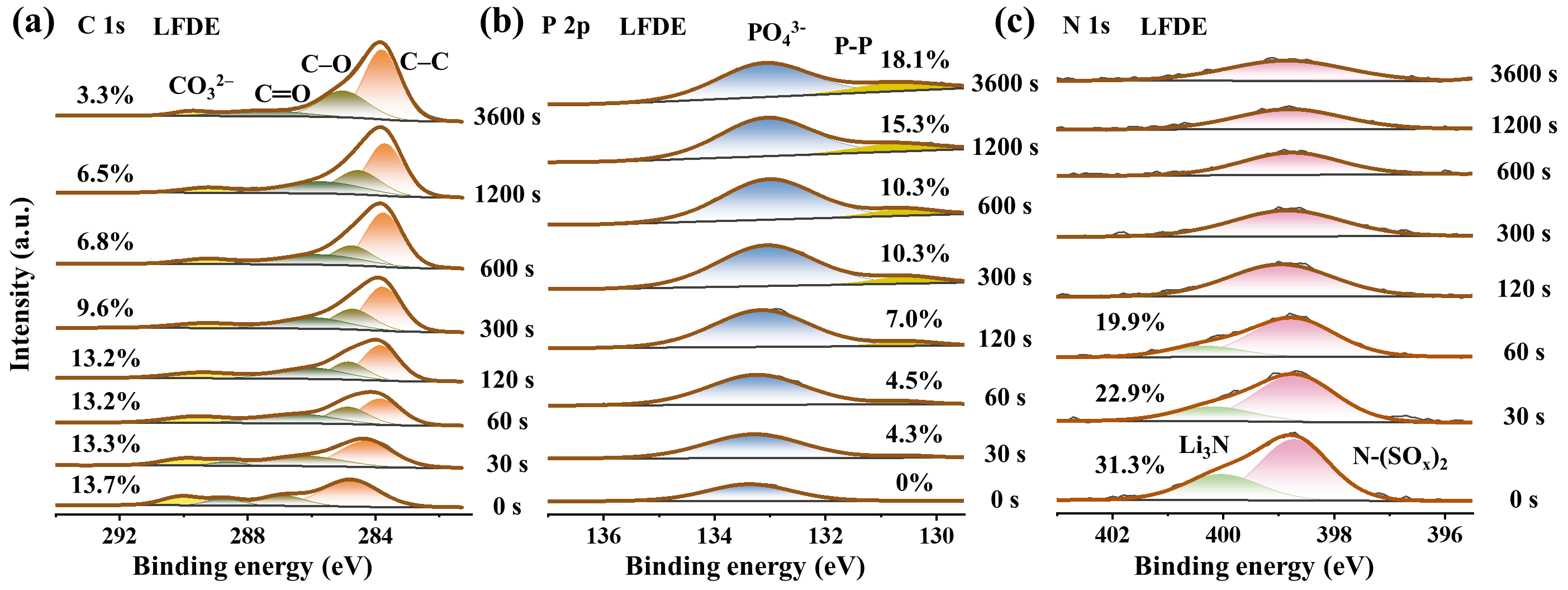


**Figure S54.** (a) C 1s, P 2p and N 1s spectra at different etching times of BP@C@LPO using LFDE after 200 cycles.


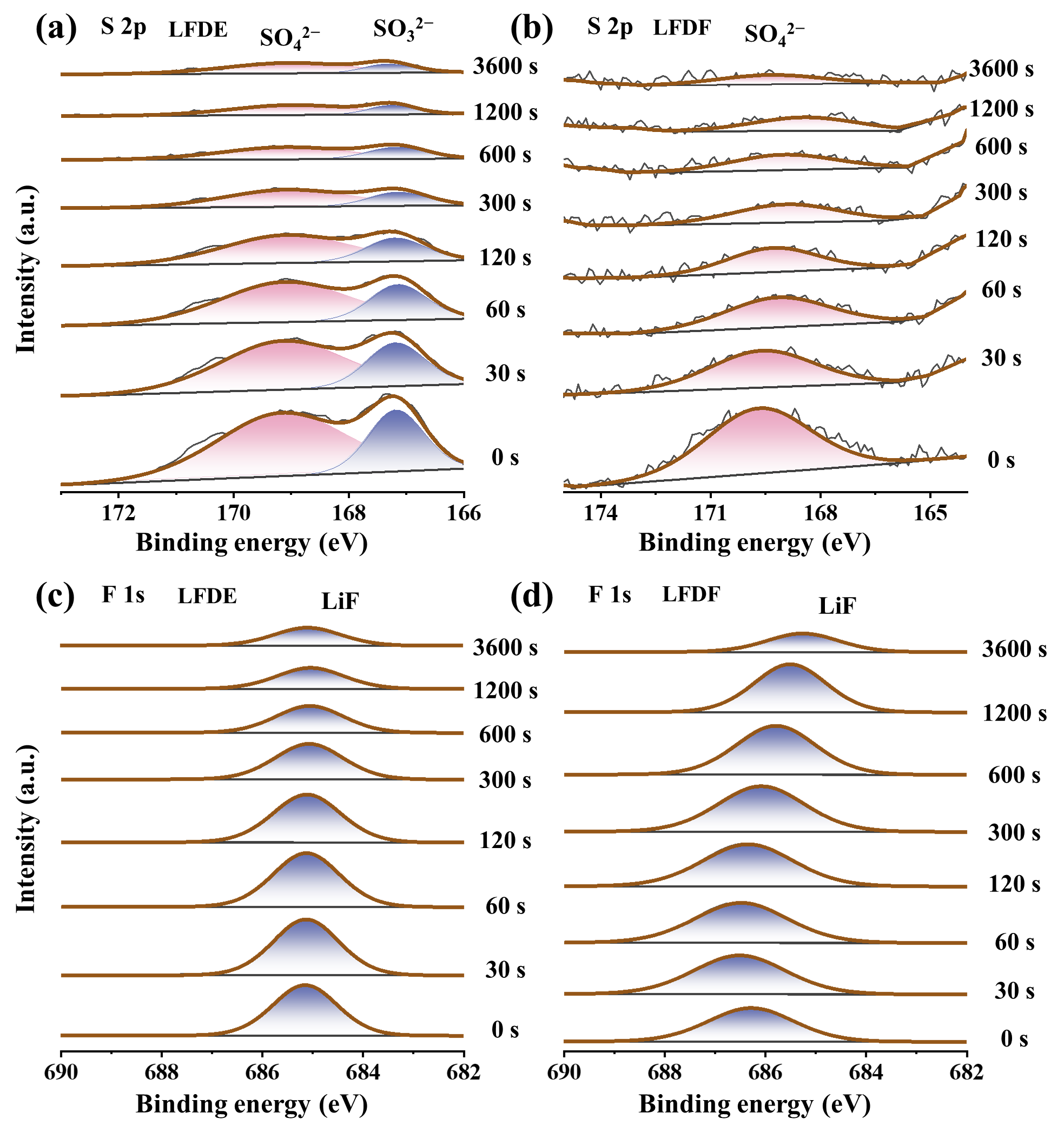


**Figure S55.** (a) C 1s, P 2p and N 1s spectra at different etching times of BP@C@LPO using LFDE after 200 cycles.

**Supplementary Table 1**: Dielectric constant (ε) and dipole moment (μ) properties of the solvents. ^[14]^

| Solvent | ε | μ |
| --- | --- | --- |
| DMC | 3.1 | 0.93 |
| EMC | 3 | 0.99 |
| DEC | 2.8 | 1.07 |
| DOL | 7.1 | 1.19 |
| TEP | 13.1 | 2.86 |
| EC | 89.8 | 4.51 |
| FEC | 78.4 | 4.97 |

**Supplementary Table 2**: The coordination number (CN) value of solvents in different electrolyte from Raman fitting results.

| Electrolyte | CN of solvent |
| --- | --- |
| LDMC | 2.05 |
| LEMC | 1.46 |
| LDEC | 0.69 |
| LFF | 3.1 |
| LFE | 3.12 |
| LFT | 2.81 |
| LFDD | 2.36 |
| LFDT | 2.5 |
| LFDE | 3.49 |
| LFDF | 3.28 |
| LFD3F | 4.16 |
| LFD5F | 4.23 |

**Supplementary Table 3**: Comparison of electrochemical performance between BP@C@ZPO and other pervious works about P-based anodes for LIBs. Note that the current densities and specific capacities are calculated based on the mass of P.

| Materials | Current density (A/g) | Capacity | Cycle number | Retention | Reference |
| --- | --- | --- | --- | --- | --- |
| BP@C@LPO | 2.6 | 2615.2 | 1000 | 91.7% | This work |
|  | 10.4 | 2210.7 | 1500 | 96.7% |  |
| 3D Hoya-like DCNM@RP anode | 5.2 | 1360.7 | 1800 | 99.8% | ^[15]^ |
| RP-PC | 1 | 2500 | 1100 | 70.4% | ^[16]^ |
| BP@CNTs hybrids | 0.5 | 914.1 | 650 | 82.8% | ^[17]^ |
| RP-porous carbon | 0.52 | 2000 | 500 | 81.3% | ^[18]^ |
| Fluffy carboncoated red phosphorus | 2.6 A/g | 1230 | 1000 | 96% | ^[19]^ |
| RP/ hierarchical micro mesoporous carbon nanospheres | 2 | 1479.8 | 1000 | 81.2% | ^[20]^ |
| RP-Ketjen Black | 1 | 1450 | 300 | 80% | ^[21]^ |
| Self-Standing Hierarchical P/CNTs@rGO | 2.6 | 1500 | 500 | 73.9% | ^[22]^ |
| P@HPCF | 1 | 2250 | 100 | 57.8% | ^[23]^ |
| Hollow RP Nanospheres | 2.6 | 1500 | 600 | 69.9% | ^[24]^ |
| BP/RGO sandwiched film | 0.1 | 2250 | 200 | 62% | ^[25]^ |
| Iodine-Doped RP | 0.2 | 1868 | 150 | 83.6% | ^[26]^ |
| RP-carbon nanotubes | 0.2 | 2000 | 100 | 84.2% | ^[27]^ |
| RP-Graphene Nanosheet Hybrids | 0.26 | 2110 | 300 | 60% | ^[28]^ |
| BP-Graphite | 0.52 | 2490 | 100 | 74.3% | ^[29]^ |
| RP-porous carbon | 0.1 | 2400 | 55 | 87% | ^[30]^ |

**Supplementary Table 4:** Comparison of electrochemical performance between WSEs in this work and other electrolyte in Phosphorus-based anodes for LIBs. Note that the current densities and specific capacities are calculated based on the mass of P phosphorus excepts for the special note.

| Electrolyte | Electrode | Current density (A g^-1^) | Capacity (mAh g^-1^) | Cycle number | Retention | Reference |
| --- | --- | --- | --- | --- | --- | --- |
| 1 M LiFSI DMC:FEC (9:1 by volume ) | BP@C@LPO | 2.6 | 2615.2 | 1000 | 91.7% | This work |
|  |  | 10.4 | 2210.7 | 1500 | 96.7% |  |
| 1 M LiPF6 DEC)/EC (1:1 by volume) | BP@CNTs hybrids | 0.5 | 914.1 | 650 | 82.8% | ^[17]^ |
| 1 M LiPF6 EC/DEC (1:1 by volume) with 5 wt. % FEC | 3D Hoya-like DCNM@RP anode | 5.2 | 1360.7 | 1800 | 99.8% | ^[15]^ |
| 1 M LiPF6 EC/EMC = 1:1, v/v) with 1% VC + 2% 1,3-PS | RP-PC | 1 | 2500 | 1100 | 70.4% | ^[16]^ |
| 1 M LiPF6 EC/DMC (1:1, v/v) | Fluffy carboncoated red phosphorus | 2.6 | 1230 | 1000 | 96% | ^[19]^ |
| 1M LiFSI TEP:FEC (7:3 by volume) | RP/CNT | 1 | 1300 (based on composite) | 100 | 57% | ^[31]^ |
| 4.5M LiFSI DME+ 3 wt.% FEC | RP/CNT | 3 | 1127 (based on composite) | 450 | 62.2% | ^[32]^ |

**References**

[1] a)D. M. Seo, O. Borodin, S.-D. Han, Q. Ly, P. D. Boyle, W. A. Henderson, *J. Electrochem. Soc.* **2012**, *159*, A553; b)D. M. Seo, O. Borodin, S.-D. Han, P. D. Boyle, W. A. Henderson, *J. Electrochem. Soc.* **2012**, *159*, A1489.

[2] D. Luo, H. Xie, F. Tan, X. Ding, J. Cui, X. Xie, C. Liu, Z. Lin, *Angew. Chem. Int. Ed.* **2022**, *61*, 202203698.

[3] J. Yan, H. Li, K. Wang, Q. Jin, C. Lai, R. Wang, S. Cao, J. Han, Z. Zhang, J. Su, K. Jiang, *Adv. Energy Mater.* **2021**, *11*, 2003911.

[4] M. He, L. Zhu, G. Ye, Y. An, X. Hong, Y. Ma, Z. Xiao, Y. Jia, Q. Pang, *Angew. Chem. Int. Ed.*  **2024**, *633*, 202415053..

[5] W. Cai, C. Yan, Y.-X. Yao, L. Xu, R. Xu, L.-L. Jiang, J.-Q. Huang, Q. Zhang, *Small Struct.* ***2020****, 1,* 2000010.

[6] J. Li, Y. Zheng, K. S. Hui, K. Wang, C. Zha, D. A. Dinh, J. Tu, Z. Shao, K. N. Hui, *Energy Storage Mater.* **2023**, *61*, 102852.

[7] W. Zou, J. Zhang, M. Liu, J. Li, Z. Ren, W. Zhao, Y. Zhang, Y. Shen, Y. Tang, *Adv. Mater.* **2024**, *36*, 2400537.

[8] Y. Lee, B. Ma, P. Bai, *Energy & Environ. Sci.* **2020**, *13*, 3504.

[9] J. Zhang, W. Huang, L. Li, C. Chang, K. Yang, L. Gao, X. Pu, *Adv. Mater.* **2023**, *35*, 2300073.

[10] F. Qiu, S. Ren, X. Zhang, P. He, H. Zhou, *Sci Bull.* **2021**, *66*, 897.

[11] X. Yue, J. Zhang, Y. Dong, Y. Chen, Z. Shi, X. Xu, X. Li, Z. Liang, *Angew. Chem. Int. Ed.* **2023**, *62,* 202306963.

[12] a)Z. Yan, Z. Huang, H. Zhou, X. Yang, S. Li, W. Zhang, F. Wang, Y. Kuang, *J. Energy Chem.* **2021**, *54*, 571; b)L. Gu, J. Han, M. Chen, W. Zhou, X. Wang, M. Xu, H. Lin, H. Liu, H. Chen, J. Chen, Q. Zhang, X. Han, *Energy Storage Mater.* **2022**, *52*, 547.

[13] Z. Liu, X. Che, W. Wang, G. Huang, W. Huang, C. Liu, Q. Liu, Y. Zhu, Z. Lin, D. Luo, *Adv. Funct. Mater.* **2024**, *34,* 2404044.

[14] a)M. Zhou, P. Bai, X. Ji, J. Yang, C. Wang, Y. Xu, *Adv. Mater.* **2021**, *33*, 2003741; b)Y. Wu, Q. Hu, H. Liang, A. Wang, H. Xu, L. Wang, X. He, *Adv. Energy Mater.* **2023**, *13,* 2300259.

[15] J. Xiao, S. Lin, N. Zhang, X. Hu, *ACS Nano* **2023**, *17*, 1597−1609.

[16] S. Zhang, C. Liu, H. Wang, H. Wang, J. Sun, Y. Zhang, X. Han, Y. Cao, S. Liu, J. Sun, *ACS Nano* **2021**, *15*, 3365.

[17] Y. Zhang, L. Wang, H. Xu, J. Cao, D. Chen, W. Han, *Adv. Funct. Mater.* **2020**, *30*, 1909372.

[18] Y. Sun, L. Wang, Y. Li, Y. Li, H. R. Lee, A. Pei, X. He, Y. Cui, *Joule* **2019**, *3*, 1080.

[19] H. Liu, S. Zhang, Q. Zhu, B. Cao, P. Zhang, N. Sun, B. Xu, F. Wu, R. Chen, *J. Mater. Chem. A* **2019**, *7*, 11205.

[20] B. Liu, Q. Zhang, L. Li, Z. Jin, C. Wang, L. Zhang, Z. M. Su, *ACS Nano* **2019**, *13*, 13513.

[21] X. Li, G. Chen, Z. Le, X. Li, P. Nie, X. Liu, P. Xu, H. B. Wu, Z. Liu, Y. Lu, *Nano Energy* **2019**, *59*, 464.

[22] J. Zhou, Z. Jiang, S. Niu, S. Zhu, J. Zhou, Y. Zhu, J. Liang, D. Han, K. Xu, L. Zhu, X. Liu, G. Wang, Y. Qian, *Chem* **2018**, *4*, 372.

[23] T. Xu, D. Li, S. Chen, Y. Sun, H. Zhang, Y. Xia, D. Yang, *Chem. Eng. J.* **2018**, *345*, 604.

[24] J. Zhou, X. Liu, W. Cai, Y. Zhu, J. Liang, K. Zhang, Y. Lan, Z. Jiang, G. Wang, Y. Qian, *Adv. Mater.* **2017**, *29,* 1700214.

[25] H. Liu, Y. Zou, L. Tao, Z. Ma, D. Liu, P. Zhou, H. Liu, S. Wang, *Small* **2017**, *13,* 1700758.

[26] W. C. Chang, K. W. Tseng, H. Y. Tuan, *Nano Lett.* **2017**, *17*, 1240.

[27] Y. Zhang, X. Rui, Y. Tang, Y. Liu, J. Wei, S. Chen, W. R. Leow, W. Li, Y. Liu, J. Deng, B. Ma, Q. Yan, X. Chen, *Adv. Energy Mater.* **2016**, *6*, 1502409.

[28] Z. Yu, J. Song, M. L. Gordin, R. Yi, D. Tang, D. Wang, *Adv Sci.* **2015**, *2*, 1400020.

[29] J. Sun, G. Zheng, H. W. Lee, N. Liu, H. Wang, H. Yao, W. Yang, Y. Cui, *Nano Lett.* **2014**, *14*, 4573.

[30] L. Wang, X. He, J. Li, W. Sun, J. Gao, J. Guo, C. Jiang, *Angew. Chem. Int. Ed.* **2012**, *51*, 9034.

[31] X. Han, J. Sun, *J. Power Sources* **2020**, *474*, 228664.

[32] X. Han, J. Sun, *Chem. Commun.* **2020**, *56*, 6047.
